# The global, regional, and national burden of pancreatitis in 195 countries and territories, 1990–2017: a systematic analysis for the Global Burden of Disease Study 2017

**DOI:** 10.1186/s12916-020-01859-5

**Published:** 2020-12-10

**Authors:** Guoqing Ouyang, Guangdong Pan, Qiang Liu, Yongrong Wu, Zhen Liu, Wuchang Lu, Shuai Li, Zheng Zhou, Yu Wen

**Affiliations:** 1 Department of Hepatobiliary Surgery, Liuzhou People’s Hospital, Liuzhou, Guangxi China; 2 Department of General Surgery, The Second Xiangya Hospital, Central South University, Changsha, Hunan China

**Keywords:** Pancreatitis, Global Burden of Disease, Prevalence, Incidence, Years lived with disability

## Abstract

**Background:**

Pancreatitis is a critical public health problem, and the burden of pancreatitis is increasing. We report the rates and trends of the prevalence, incidence, and years lived with disability (YLDs) for pancreatitis at the global, regional, and national levels in 195 countries and territories from 1990 to 2017, stratified by sex, age, and sociodemographic index (SDI).

**Methods:**

Data on pancreatitis were available from the Global Burden of Diseases, Injuries, and Risk Factors Study (GBD) 2017. Numbers and age-standardized prevalence, incidence, and YLDs’ rates per 100,000 population were estimated through a systematic analysis of modeled data from the 2017 GBD study. Both acute and chronic pancreatitis are being modeled separately in the GBD 2017; however, our data show acute and chronic pancreatitis together. Estimates were reported with uncertainty intervals (UIs).

**Results:**

Globally, in 2017, the age-standardized rates were 76.2 (95% UIs 68.9 to 83.4), 20.6 (19.2 to 22.1), and 4.5 (2.3 to 7.6) per 100,000 population for the point prevalence, incidence, and YLDs, respectively. From 1990 to 2017, the percent changes in the age-standardized prevalence and YLDs rates increased, whereas the age-standardized incidence rate decreased. The global prevalence increased with age up to 60–64 years and 44–49 years in females and males, respectively, and then decreased, with no significant difference between females and males. The global prevalence rate increased with age, peaking in the 95+ age group, with no difference between sexes. Generally, positive correlation between age-standardized YLDs and SDIs at the regional and national levels was observed. Slovakia (297.7 [273.4 to 325.3]), Belgium (274.3 [242.6 to 306.5]), and Poland (266.7 [248.2 to 284.4]) had the highest age-standardized prevalence rates in 2017. Taiwan (Province of China) (104.2% [94.8 to 115.2%]), Maldives (72.4% [66.5 to 79.2%]), and Iceland (64.8% [57.2 to 72.9%]) had the largest increases in age-standardized prevalence rates from 1990 to 2017.

**Conclusions:**

Pancreatitis is a major public health issue worldwide. The age-standardized prevalence and YLDs rates increased, but the age-standardized incidence rate decreased from 1990 to 2017. Improving the quality of pancreatitis health data in all regions and countries is strongly recommended for better monitoring the burden of pancreatitis.

## Background

Despite increasing medical knowledge and new effective treatments, pancreatitis remains a critical public health problem [1, 2]. The incidence of acute pancreatitis ranges from 13 to 45 per 100,000 population-years and that of chronic pancreatitis ranges from 5 to 12 per 100,000 population-years [3]. Recently, the burden of pancreatitis has been demonstrated in several studies considering only regional and/or national factors; however, the burden of pancreatitis has not been analyzed across all countries [4–6].

In 2018, the WHO disclosed the latest global-, regional-, and country-level estimates of cause-specific years of life lost (YLLs), years lived with disability (YLDs), and disability-adjusted life year (DALYs) for pancreatitis by age and sex [7]; however, no article addressing these data has been published. A systematic review, meta-analysis, and meta-regression analysis of population-based cohort studies reported global and regional burden of acute and chronic pancreatitis in terms of incidence and death [2], but the prevalence, YLDs, and national-level information were not provided. In addition, the association between the burden of pancreatitis and the sociodemographic index (SDI) of countries was not analyzed in that study. To date, no study has reported the annual trends of the prevalence, incidence, and YLDs of pancreatitis over time.

In this study, utilizing data reported in the Global Burden of Diseases, Injuries, and Risk Factors Study (GBD) 2017, we first conducted a comprehensive and comparable analysis of the global-, regional-, and national-level incidence, prevalence, and YLDs of pancreatitis in terms of numbers and age-standardized rates (ASRs) from 1990 to 2017, stratified by sex, age, and SDI. Accurate information about the burden of pancreatitis from different regions might be valuable for policy makers to decrease the burden of pancreatitis.

## Methods

### Overview

The GBD 2017 systematically analyzed 354 diseases and injuries, 282 causes of death, and 84 risk factors for 195 countries and territories, 21 regions, and 7 super-regions from 1990 to 2017 [8]. All the data analyzed in this study are available in the GBD 2017, which was conducted by the Institute for Health Metrics and Evaluation (IHME). The data analyzed in our study, including incidence, prevalence, and YLDs, were obtained from the Global Health Data Exchange query tool (http://ghdx.healthdata.org/gbd-results-tool). The general methodology of the GBD 2017 was applied, with the latest updates described in previous GBD 2017 publications [8–11]. This study adhered to the Guidelines for Accurate and Transparent Health Estimates Reporting (GATHER) statement [12].

### Case definition and data sources

In GBD 2017, pancreatitis was defined as inflammation of the pancreas. Acute pancreatitis includes active inflammation and injury to the pancreas, leading to severe upper abdominal pain and nausea, vomiting, inappropriate release of pancreatic juice, or a systemic inflammatory response syndrome with fever, low blood pressure, and in some cases, failure of one or more organs. Chronic pancreatitis was defined as permanent damage to the pancreas from long-term or recurrent inflammation. It can present as chronic or episodic abdominal pain, nausea, and ultimately failure of the pancreas to produce and release digestive enzymes and hormones, leading to chronic diarrhea, poor absorption of foods and nutrients, and diabetes. Patients with chronic pancreatitis may experience recurrent episodes of acute pancreatitis [8]. In a previous cycle of the GBD study, the IHME modeled chronic and acute pancreatitis together, but in GBD 2017, they separated these two diseases.

The common database of acute and chronic pancreatitis included scientific literature and hospital discharges data (from numerous countries), and insurance claim data for inpatient encounters (from the USA and Taiwan). In addition, the chronic database also included outpatient facility data in the USA and Sweden and data from insurance claims for outpatient encounters. The exclusion criteria were as follows: subpopulations that clearly do not represent the national population, self-reported data, and reviews rather than original studies. Studies were added to either the acute or chronic database if they employed appropriate International Classification of Diseases (ICD) codes or a combination of clinical, biochemical, and radiographic criteria. Details on data adjustment are shown in Additional file 1: Section 1 [8].

Data acquired in the GBD Study 2017 for acute pancreatitis included incidence (site-years = 1362) and prevalence (site-years = 16), and for chronic pancreatitis included incidence (site-years = 14) and prevalence (site-years = 1401); these data were employed to calculate the study estimates [8]. A site-year, the unique combination of a calendar year and location, was defined as a country or other subnational geographical unit contributing data in a given year. The numbers of countries with data for estimating the incidence (*n* = 42) and prevalence (*n* = 5) of acute pancreatitis and incidence (*n* = 6) and prevalence (*n* = 35) of chronic pancreatitis varied. Sixteen and 4 out of 21 GBD regions provided data for estimating the incidence and prevalence of acute pancreatitis, respectively. Four and 13 out of 21 GBD regions provided data for estimating the incidence and prevalence of chronic pancreatitis, respectively. The data sources used in estimating the burden of pancreatitis in different countries can be found with the GBD 2017 data input source tool (http://ghdx.healthdata.org/gbd-2017/data-input-sources) [8].

### Data processing and disease modeling

DisMod-MR 2.1, a Bayesian meta-regression tool, was used to analyze the incidence and prevalence data for both acute and chronic pancreatitis by pooling the available heterogeneous data. DisMod-MR 2.1 is able to conduct age-integration; however, its performance decreased while integrating across wide age groups (e.g., all ages). To address this issue, the data, run by the DisMod-MR 2.1 model, was disaggregated by age to calculate countries’ age-pattern and then applied the calculated age-pattern to split aggregated all age data (Additional file 1:Section 2) [8, 13].

For acute pancreatitis, the reference incidence data were adjusted with Taiwan claims data and hospital discharge data. Study-level covariates of interest were extracted from the literature data and US claims data for patients with a first attack of pancreatitis; those who experienced acute pancreatitis but had preexisting chronic pancreatitis were excluded. The value prior to remission, which was set to 0 for all age groups, in the DisMod-MR 2.1 model ranged from 8 to 9 (a duration from approximately 6 weeks) for all ages. The location-level covariates consisted of per capita alcohol consumption, the log-normalized age-standardized death rate due to pancreatitis (both for incidence), and the Healthcare Access and Quality index (for the excess mortality rate) (Additional file 1:Table S1) [8].

For chronic pancreatitis, the IHME adjusted the reference prevalence data with hospital discharge data and Taiwan claims data. Study-level covariates of interest were extracted from the literature data, US claims data, and outpatient data from the USA and Sweden. DisMod-MR 2.1 was employed to extract cause-specific mortality rate (CSMR) data from the CODEm and CODcorrect analyses and match with prevalence and incidence data points for the same geography. The prior value of remission was set to 0. The log-transformed age-standardized scaled exposure variable (scalar covariate) for pancreatitis prevalence and the Healthcare Access and Quality index were included as location covariates (Additional file 1: Table S2) [8].

### Severity and YLDs

ICD-10 codes (K85-K86.9) and ICD-9 codes (577–577.9) were used to identify pancreatitis cases. The severity levels of five sequelae were estimated, with disability weights (DWs) considered as a weight factor ranging from 0 to 0.324 (see online supplementary Table S3) [8].

The pancreatitis prevalence and acute episode data were assigned a single, combined DW for severe infection and severe abdominal pain symptoms. All the prevalent cases in the chronic pancreatitis disease model were assigned to symptomatic and asymptomatic groups using proportions found in a previous study. Medical Expenditure Panel Surveys (MEPS) were used to identify the proportion of each of the severity splits for pancreatitis. Severity splits are typically divided into symptomatic and asymptomatic. The proportions established by the MEPS were used to split the symptomatic group into three subgroups: mild, moderate, and severe. The prevalence of severity in each group was multiplied by a severity-specific DW to estimate the YLDs [8, 14].

### Compilation of results

YLLs were multiplied by the difference between the standard life expectancy for a given age and the sum of deaths in each age group [9]. DALYs were calculated by summing the YLLs and YLDs [8, 14]. Uncertainty was accounted for by performing 1000 ordered draws at each computational step, combining the uncertainty from various sources, such as input data, corrections for measurement errors, and estimates of residual non-sampling errors. The 95% uncertainty intervals (UIs) were determined based on the 2.5th and 97.5th percentiles across the ordered draws. The flowcharts of estimation for acute pancreatitis and chronic pancreatitis are shown in Additional file 1: Figure 2 [8].

Smoothing spline models were employed to determine the shape of the correlation curve between pancreatitis burden in terms of YLDs and SDIs for 21 regions and 195 countries and territories [15]. The SDI is a value ranging from 0 (worst) to 1.0 (best) and was calculated from the total fertility rate among those under 25 years old, mean education level for the population over 15 years old, and lag-distributed income per capita (LDI) [11]. All statistics were generated by R software version 3.6.3 and visualized using the ggplot2 3.3.0 package [16]. The differences between sexes were compared with an unpaired *t* test. A *P* value < 0.05 was considered statistically significant.

## Results

### Prevalence of pancreatitis

Globally, the number of prevalent cases of pancreatitis was 3,038,787 (95% UI 2,768,128 to 3,307,165) in 1990 and 6,115,833 (95% UI 5,533,925 to 6,704,070) in 2017, with an age-standardized prevalence rate of 67.2(61.3 to 73.1) in 1990 and 76.2 (68.9 to 83.4) in 2017 per 100,000 population; this rate increased by 13.3% (10.3 to 16.4%) from 1990 to 2017 (Table 1).

**Table 1:** Prevalent cases, incident cases, and years lived with disability (YLD) for pancreatitis in 2017 for both sexes and percentage change of age-standardized rates (ASR) per 100,000 population from 1990 to 2017 by Global Burden of Disease regions

	Prevalence (95% uncertainty interval)			Incidence (95% uncertainty interval)			YLDs (95% uncertainty interval)		
	Counts	ASR per 100,000 population (95% UI)	Percentage change in ASRs per 100,000 population (95% UI)	Counts	ASR per 100,000 population (95% UI)	Percentage change in ASRs per 100,000 population (95% UI)	Numbers	ASR per 100,000 population (95% UI)	Percentage change in ASRs per 100,000 population (95% UI)
Global	6,115,833 (5,533,925 to 6,704,070)	76.2 (68.9 to 83.4)	13.3 (10.3 to 16.4)	1,644,222 (1,525,569 to 1,769,526)	20.6 (19.2 to 22.1)	− 6 (− 7.6 to − 4.2)	364,447 (186,273 to 612,755)	4.5 (2.3 to 7.6)	9.2 (5.5 to 12.4)
Andean Latin America	39,623 (35,598 to 43,492)	70.2 (63.1 to 77.1)	28.2 (22.3 to 33.8)	11,537 (10,685 to 12,469)	20.0 (18.5 to 21.6)	− 3.7 (− 5.9 to − 1)	2370 (1211 to 3990)	4.2 (2.1 to 7)	22.5 (9.6 to 35.4)
Australasia	25,152 (22,429 to 27,862)	64.3 (57.3 to 71.7)	26.1 (21.2 to 30.8)	9540 (8740 to 10,416)	25.4 (23.2 to 27.6)	− 4.8 (− 6.9 to − 2.7)	1606 (850 to 2679)	4.2 (2.2 to 6.9)	17.8 (2.4 to 31.7)
Caribbean	43,323 (38,842 to 47,732)	86.4 (77.4 to 95.1)	40.8 (36.5 to 45)	8371 (7750 to 9029)	16.9 (15.6 to 18.2)	7.1 (5.2 to 9.3)	2438 (1192 to 4177)	4.9 (2.4 to 8.3)	35.5 (24.9 to 45.4)
Central Asia	111,530 (99,718 to 124,267)	129.5 (115.9 to 143.9)	13.5 (8.8 to 17.9)	11,424 (10,552 to 12,355)	12.9 (12 to 13.9)	3.4 (1.1 to 5.7)	6067 (2856 to 10,616)	7 (3.3 to 12.2)	13.1 (3.7 to 23)
Central Europe	383,787 (352,525 to 414,563)	222.1 (205 to 239.8)	21.4 (16.8 to 26.3)	68,446 (64,051 to 73,101)	42.8 (40 to 45.6)	2. 1(− 0.6 to 5.5)	21,277 (10,479 to 36,432)	12.5 (6.1 to 21.4)	19 (11.5 to 26.6)
Central Latin America	188,560 (169,971 to 206,572)	78.3 (70.5 to 85.8)	21.4 (17 to 25.8)	60,554 (55,680 to 65,707)	24.4 (22.5 to 26.3)	3.5 (2 to 5.1)	11,472 (59 68 to 19,250)	4.7 (2.4 to 7.9)	17.9 (11.9 to 23.7)
Central sub-Saharan Africa	15,214 (13,313 to 17,228)	18.6 (16.3 to 20.8)	10.3 (7.2 to 13.2)	2556 (2303 to 2831)	3.1 (2.8 to 3.4)	2.8 (0.5 to 5.1)	907 (417 to 1622)	1.1 (0.5 to 2)	9.9 (7 to 12.7)
East Asia	1,159,508 (1,034,974 to 1,293,409)	59 (52.9 to 65.5)	34.9 (31.2 to 38.9)	429,342 (393,662 to 468,409)	22.7 (20.9 to 24.6)	5.1 (3.1 to 7.1)	73,112 (39,225 to 120,354)	3.7 (2 to 6.2)	26.7 (20.4 to 31.9)
Eastern Europe	614,456 (558,090 to 677,548)	213.8 (194.5 to 235.3)	51.8 (46.5 to 57)	135,287 (124,571 to 146,178)	50.0 (46.1 to 53.7)	22 (20.5 to 23.5)	34,940 (17,606 to 60,267)	12.3 (6.1 to 21.2)	47.5 (40.3 to 54)
Eastern sub-Saharan Africa	46,949 (41,160 to 52,900)	18.7 (16.4 to 20.9)	15.9 (13.4 to 18.6)	7974 (7155 to 8856)	3.0 (2.8 to 3.3)	5.9 (4.1 to 8)	2799 (1291 to 5030)	1.1 (0.5 to 2)	15.3 (12.8 to 17.9)
High-income Asia Pacific	424,150 (383,580 to 470,105)	154.4 (139.8 to 171)	3.1 (− 1.3 to 6.9)	72,075 (66,885 to 77,347)	29.7 (27.7 to 31.8)	2.1 (− 0.1 to 4.6)	24,456 (12,340 to 41,094)	9.1 (4.7 to 15.5)	3 (− 2.7 to 8.3)
High-income North America	445,619 (404,471 to 484,509)	93 (84.6 to 100.6)	7 (2.5 to 11.4)	284,791 (266,555 to 303,177)	60.2 (56.5 to 63.9)	− 5.8 (− 9.7 to − 1.2)	31,290 (18,049 to 49,920)	6.6 (3.9 to 10.5)	1.7 (− 4 to 6.7)
North Africa and Middle East	249,481 (219,520 to 279,783)	49 (43.2 to 54.4)	27.2 (24.3 to 30.1)	53,000 (48,513 to 57,892)	10.0 (9.2 to 10.8)	6.5 (5.3 to 7.8)	14,629 (7146 to 25,120)	2.8 (1.4 to 4.8)	22.8 (15.6 to 29.4)
Oceania	2706 (2374 to 3035)	31 (27.4 to 34.6)	41 (36.6 to 45.3)	1064 (966 to 1174)	11.3 (10.4 to 12.3)	1.6 (0.1 to 3.3)	173 (90 to 294)	1.9 (1 to 3.3)	30.3 (21.6 to 38.4)
South Asia	824,315 (728,591 to 926,570)	50.4 (44.7 to 56.2)	51.6 (48.7 to 54.4)	180,789 (165,583 to 197,571)	10.9 (10 to 11.8)	23.4 (21.8 to 25)	48,453 (23,785 to 85,613)	2.9 (1.4 to 5.1)	41.4 (35.8 to 47.3)
Southeast Asia	314,005 (277,968 to 350,287)	49.3 (43.9 to 54.8)	37.7 (33.6 to 41.6)	89,193 (82,195 to 96,841)	13.8 (12.7 to 14.9)	1.3 (0.2 to 2.5)	18,788 (9616 to 31,897)	2.9 (1.5 to 4.9)	30 (22.4 to 36.8)
Southern Latin America	30,741 (27,476 to 34,098)	40.8 (36.4 to 45.4)	7.5 (2.4 to 12.8)	15,022 (13,732 to 16,360)	20.1 (18.4 to 21.9)	− 8.8 (− 10.5 to − 6.7)	2090 (1145 to 3364)	2.8 (1.5 to 4.5)	3.3 (− 8.6 to 16.3)
Southern sub-Saharan Africa	12,884 (11,300 to 14,458)	18.8 (16.5 to 21)	5.4 (2.6 to 8.1)	2054 (1859 to 2253)	3.0 (2.7 to 3.2)	− 1.2 (− 2.9 to 0.5)	768 (355 to 1375)	1.1 (0.5 to 2)	5.2 (2.4 to 7.8)
Tropical Latin America	387,362 (352,794 to 422,360)	167 (152.3 to 182.1)	− 8.1 (− 11.2 to − 4.9)	47,034 (44,043 to 50,327)	20.2 (18.9 to 21.6)	1.2 (− 1.3 to 3.9)	20,701 (9974 to 35,370)	8.9 (4.3 to 15.2)	− 7.8 (− 12.4 to − 2.8)
Western Europe	716,616 (644,467 to 791,137)	111.8 (100.5 to 124.4)	27.3 (21.9 to 32.4)	140,633 (130,519 to 151,415)	23.3 (21.6 to 25.1)	4.2 (2.3 to 6.7)	41,460 (21,090 to 70,249)	6.6 (3.3 to 11.3)	23.8 (16.1 to 31.4)
Western sub-Saharan Africa	79,851 (69,618 to 90,046)	29 (25.3 to 32.5)	11.8 (8.1 to 15.3)	13,537 (12,306 to 14,940)	4.8 (4.4 to 5.2)	7.5 (5 to 10.1)	4652 (2184 to 8343)	1.7 (0.8 to 3)	9.2 (1.9 to 18)

**Fig. 1:**
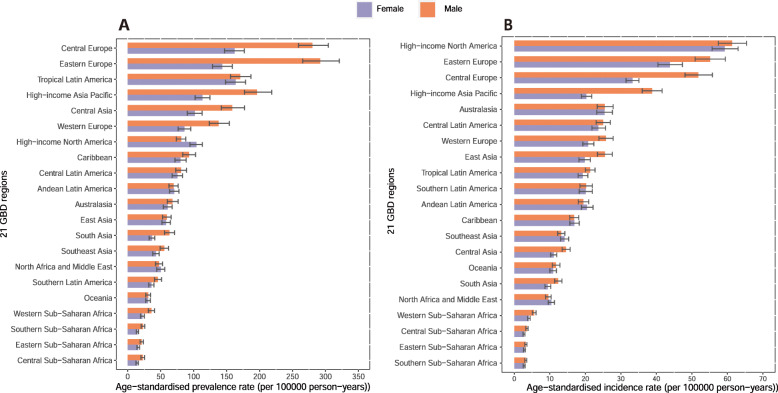
The age-standardized prevalence (**a**) and incidence rates (**b**) of pancreatitis in 2017 for 21 GBD regions, by sex

At the regional level, the highest age-standardized prevalence rates of pancreatitis per 100,000 population were observed in Central Europe (222.1 [205.0 to 239.8]), Eastern Europe (213.8 [194.5 to 235.3]), and Tropical Latin America (167.0 [152.3 to 182.1]). In contrast, the lowest age-standardized prevalence rates were observed in southern sub-Saharan Africa (18.9 [16.5 to 21.0]), eastern sub-Saharan Africa (18.7 [16.4 to 20.9]), and central sub-Saharan Africa (18.6 [16.3 to 20.8]) (Table 1, Fig. 1a). The percent changes in the age-standardized prevalence rates from 1990 to 2017 were different across the 21 GBD regions (Additional file 2: Fig. S1). All GBD 2017 regions except Tropical Latin America (− 8.1% [− 4.9 to − 11.2%]) showed increasing trends in the age-standardized prevalence rate between 1990 and 2017. The highest percent changes in the age-standardized prevalence rates were observed in Eastern Europe (51.8% [46.5 to 57.0%]), South Asia (51.6% [48.7 to 54.4%]), and Oceania (41.0% [36.5 to 45.0%]). We also found that the contribution to the number of prevalent cases varied across the 21 GBD regions. The highest number of prevalent cases was found in East Asia, South Asia, and Western Europe (Table 1, Additional file 2: Fig. S2).

**Fig. 2:**
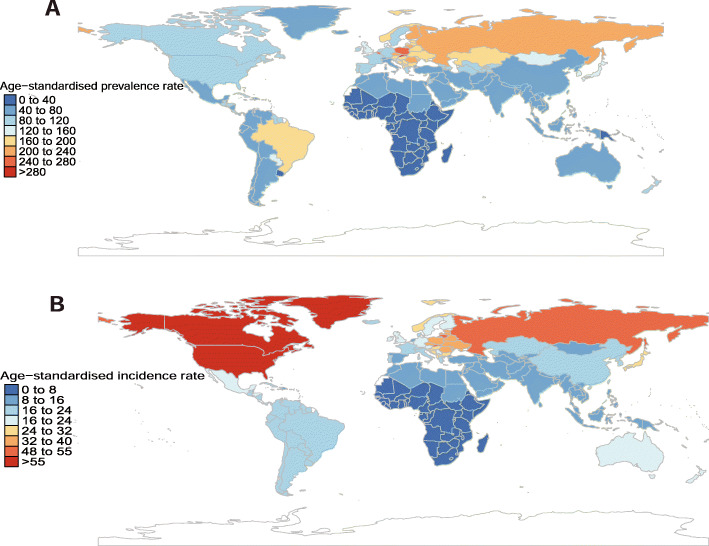
Age-standardized prevalence (**a**) and incidence rates (**b**) of pancreatitis per 100,000 population in 2017, by country and territory

The national estimated age-standardized prevalence rates of pancreatitis ranged from 16.9 to 297.7 cases per 100,000 population in 2017. The countries with the highest age-standardized prevalence estimates were Slovakia (297.7 [273.4 to 325.3]), Belgium (274.3 (242.6 to 306.5]), and Poland (266.7 (248.2 to 284.4]). In contrast, the countries with the lowest age-standardized prevalence estimates were the Central African Republic (16.9 [14.9 to 18.9]), Somalia (17.5 [15.4 to 19.5]), and Burundi (17.6 [15.3 to 19.7]) (Additional file 1: Table S4 and Fig. 2a). The percent changes in age-standardized prevalence rate estimates varied among countries and territories between 1990 and 2017, with the largest increase in Taiwan (Province of China) (104.2% [94.8 to 115.2%]), the Maldives (72.4% [66.5 to 79.2%]), and Iceland (64.8% [57.2 to 72.9%]). In contrast, Moldova (− 15.8% [− 20.7 to − 10.7%]), Austria (− 13.1% [− 19.2 to − 6.8%]), and Brazil (− 8.6% [− 11.8 to − 5.4%]) had the largest decreases from 1990 to 2017 (Additional file 1: Table S4).

### Incidence of pancreatitis

Globally, there were an estimated 1,010,993 (923,565 to 1,101,077) new cases of pancreatitis in 1990 and 1,644,222 (1,525,569 to 1,769,526) new cases of pancreatitis in 2017, with an age-standardized incidence rate of 21.9 (20.1 to 23.8) in 1990 and 20.6 (19.2 to 22.1) in 2017 per 100,000 population; this rate decreased by 6.0% (4.2 to 7.6%) from 1990 to 2017 (Table 1).

At regional level, the highest age-standardized incidence rate of pancreatitis per 100,000 persons were observed in high-income North America (60.2 [56.5 to 63.9]), Eastern Europe (50.0 [46.1 to 53.7]), and Central Europe (42.8 [40.0 to 45.6]). In addition, southern sub-Saharan Africa (3.0 [2.7 to 3.2]), eastern sub-Saharan Africa (3.0 [2.8 to 3.3]), and central sub-Saharan Africa (3.1 [2.8 to 3.4]) exhibited the lowest age-standardized incidence rates (Table 1, Fig. 1b). From 1990 to 2017, the highest increase in the age-standardized incidence rates was in South Asia (23.4% [21.9 to 25.0%]), Eastern Europe (22.0% [20.5 to 23.5%]), and western sub-Saharan Africa (7.5% [5.0 to 10.1%]) Asia (Additional file 2: Fig. S3). In addition, the highest number of incident cases was found in East Asia, high-income North America, and South Asia (Table 1, Additional file 2: Fig. S4).

The national estimated age-standardized incidence rate of pancreatitis in 2017 ranged from 2.80 to 60.3 cases per 100,000 population. The USA (60.34 [56.8 to 64.0]), Canada (59.0 [53.5 to 64.4]), and Greenland (56.6 [51.6 to 61.4]) had the highest age-standardized incidence rates in 2017, whereas Djibouti (2.8 [2.5 to 3.1]), Madagascar (2.8 [2.5 to 3.1]), and South Sudan (2.9 [2.6 to 3.2]) had the lowest incidence rates in 2017 (Additional file 1: Table S5 and Fig. 2b). From 1990 to 2017, the largest increases in the age-standardized incidence rates were found in Lithuania (31.9% [27.6 to 36.7%]), Georgia (30.6% [26.8 to 34.6%]), and India (28.7% [26.9 to 30.6%]), whereas the largest decreases in the age-standardized incidence rates were found in Slovenia (− 14.8% [− 18.0 to − 11.5%]), Hungary (− 13.9% [− 16.3 to − 11.5%]), and Argentina (− 12.2% [14.1 to − 10.2%]) (Additional file 1: Table S5).

### YLDs of pancreatitis

The global estimated number of YLDs of pancreatitis in 1990 was 189,382 (99,346 to 317,452) and in 2017 was 364,447 (186,273 to 612,755), with an age-standardized YLDs rate of 4.2 (2.2 to 6.9) in 1990 and 4.5 (2.3 to 7.6) in 2017 per 100,000 population; this rate increased by 9.2% (5.5 to 12.4%) from 1990 to 2017 (Table 1).

Central Europe (12.5 [6.1 to 21.4]), Eastern Europe (12.28 [6.1 to 21.2]), and high-income Asia Pacific (9.1 [4.7 to 15.5]) were found to have the highest age-standardized YLDs’ rate of pancreatitis per 100,000 population in 2017, whereas central sub-Saharan Africa (1.1 [0.5 to 2.0]), eastern sub-Saharan Africa (1.11 [0.5 to 2.0]), and southern sub-Saharan Africa (1.1 [0.5 to 2.0]) had the lowest age-standardized YLDs rates per 100,000 population (Additional file 2: Fig.S5). In addition, Eastern Europe (47.5% [40.3 to 54.0%]), South Asia (41.4% [35.8 to 47.3%]), and the Caribbean (35.5% [25.0 to 45.4%]) had the largest increases in the age-standardized YLDs’ rates between 1990 and 2017(Additional file 2: Fig.S6).

At national level, the age-standardized YLDs’ rate ranged from 1.0 to 16.6 cases per 100,000 population. The countries with the highest age-standardized YLDs rates were the same as those with the highest the age-standardized prevalence rates (Additional file 1: Table S6 and Additional file 2: Fig. S7). The largest increase in the age-standardized YLDs rate was found in Taiwan (Province of China) (77.4% [48.5 to 104.7%]), followed by the Maldives (57.6% [33.0 to 84.4%]) and Belgium (52.9% [28.9 to 77.3%]). In contrast, Moldova (− 14.5% [− 26.3 to − 1.5%]), Austria (− 12.0% [− 24.9 to − 2.0%]), and Brazil (− 8.3% [− 13.0 to − 3.3%]) had the largest decreases in YLDs rates (Additional file 1: Table S6).

### Age and sex patterns

**Fig. 3:**
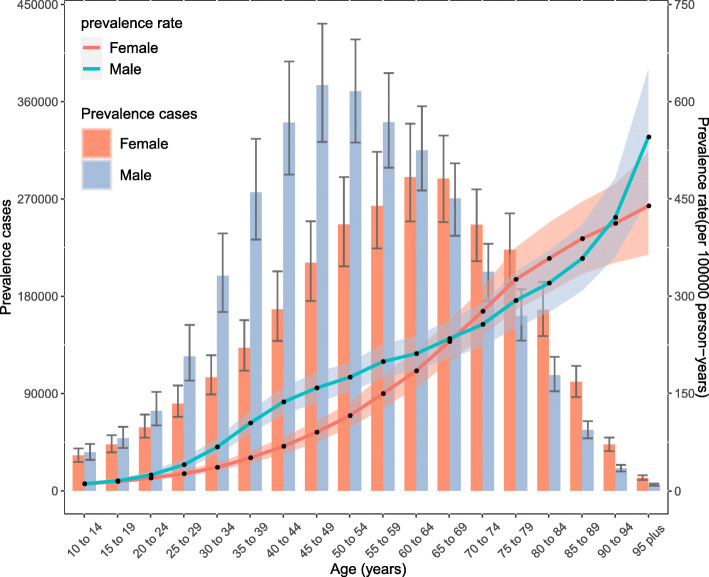
Global cases and age-standardized rates of prevalence of pancreatitis per 100,000 population by age and sex, 2017. Shading indicates the upper and lower limits of the 95% uncertainty intervals (95% UIs)

No statistically significant differences in the incidence, prevalence, or YLDs were observed between women and men in all age groups. The prevalence rate increased with age, peaking in the 95 plus age group in both females and males in 2017. However, the number of prevalent cases increased with age, reaching its highest level in the 60–64 and 45–49 age groups for females and males, respectively, after which the trend decreased with increasing age. The number of prevalent cases was higher in males than in females at the age of 60–64 years, after which the number in males was lower than that in females. The number of prevalent cases was lower in both sexes below 20 years age and above 90 years age (Fig. 3). The incidence rate increased with age, peaking in the 95 plus age group for both females and males in 2017. The number of incident cases peaked in the 60–64 age group in females, whereas the peak in males occurred in the 40–44 age group. The numbers of incident cases were also higher in males younger than 55 years than in females, whereas the numbers of incident cases were lower in males than females in the 55 years and older age group. The lowest incident case was found in patients younger than 20 years and older than 90 years (Additional file 2: Fig.S8). The patterns of the YLDs rates and numbers by sex and age group were similar to the prevalence patterns (Additional file 2: Fig. S9).

### Burden of pancreatitis by SDI

**Fig. 4:**
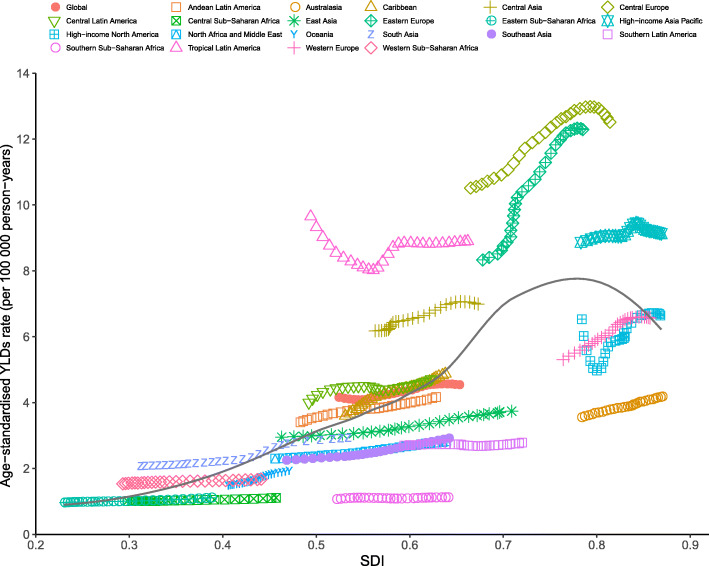
Age-standardized YLDs rates for pancreatitis for 21 GBD regions by SDI, 1990–2017. Expected values based on Socio-demographic Index and disease rates in all locations are shown as the black line. YLDs = years lived with disability. GBD, Global Burden of Diseases, Injuries, and Risk Factors Study; SDI, sociodemographic index

Generally, a positive correlation between the age-standardized YLDs rates of pancreatitis and the SDIs at the global level and across all GBD regions from 1990 to 2017 was detected. At the global level, the observed burden of pancreatitis was higher than the expected level in patients from regions with lower SDIs; however, this was the opposite in patients from regions with higher SDIs. At the regional level, observed burden estimates of pancreatitis in high-income Asia, Central Europe, Eastern Europe, Tropical Latin American, and Central Asia were higher than the expected level based on the SDIs from 1990 to 2017. However, this was not the case for most of the remaining regions (Fig. 4).

**Fig. 5:**
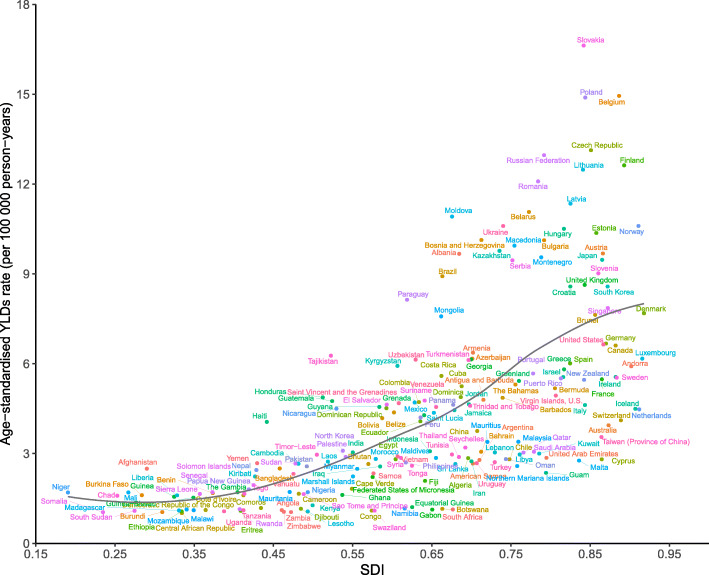
Age-standardized YLDs rates for pancreatitis for 195 countries and territories by SDI, 2017. Expected values based on Socio-demographic Index and disease rates in all locations are shown as the black line. YLDs, years lived with disability; SDI, sociodemographic index

At the national level, there was also a generally positive correlation between age-standardized YLDs rates and SDIs for pancreatitis in 2017. The burden of pancreatitis in Slovakia, Belgium, Poland, the Czech Republic, the Russian Federation, Finland, and many other countries or territories were much higher than the expected levels, whereas in the Central African Republic, Somalia, and Zambia, the burden were much lower than the expected levels based on the SDIs (Fig. 5). Positive correlations between the SDI and age-standardized incidence and prevalence rates of pancreatitis were also observed (see online Additional file 2: Fig. S10 and Fig. S11).

## Discussion

In this study, we reported the prevalence, incidence, and YLDs numbers and ASRs for pancreatitis in 195 countries and territories over a 28-year period from 1990 to 2017. Globally, there were approximately 6.12 million prevalent cases, 1.64 million incident cases, and 0.06 million YLDs in 2017. The ASRs of prevalence, incidence, and YLDs were 76.2, 20.6, and 4.5 per 100,000 population, respectively.

A previous systematic review reported that the global estimated incidence was 33.7 cases (95% CI 23.3 to 48.8) per 100,000 population-years and 9.6 cases (95% CI 7.9 to 11.8) per 100,000 population-years for acute pancreatitis and chronic pancreatitis, respectively [2]. In our study, the age-standardized incidence rate for pancreatitis was 20.6 per 100,000 population-years in 2017. The GBD 2013 also reported an age-standardized pancreatitis incidence rate of 251.0 per 100,000 population in 2013 [17]. However, the results of these two studies could not be directly compared with our results due to differences in methodologies and data sources among those three studies. For example, GBD 2013 employed DisMod-MR 2.0; however, GBD 2017 used DisMod-MR 2.1, to pool the available data. Moreover, the GBD 2017 modeled acute and chronic pancreatitis separately; in contrast, GBD 2013 modeled them together. Additionally, the incidence rate reported in Xiao’s systematic review was not age-standardized, which might have affected the results if regions or countries had a relatively young population, on average. The regional incidence rate estimates derived by Xiao were 58.2 and 7.71 per 100,000 population-years for acute and chronic pancreatitis, respectively, in the USA, where the rate of pancreatitis was higher than that in other included regions [2]. In our study, the highest age-standardized incidence rate was also found in the USA.

In the GBD 2013, the percent changes in the age-standardized prevalence, incidence, and YLDs rates of pancreatitis increased from 1990 to 2013 [17]. From 1990 to 2017, although the percent change in the age-standardized incidence rate of pancreatitis decreased, the age-standardized prevalence and YLDs showed increasing trends. The percent changes in prevalent cases, incident cases, and YLDs in GBD 2013 and in our study showed increasing trends, suggesting that the global burden of pancreatitis increased with time [17]. Therefore, it is urgent that pancreatitis prevention measures, management, and treatment are prioritized by policy makers. Differences in the regional prevalence and incidence of pancreatitis should be noted. Both Central Europe and Eastern Europe had the highest prevalence and incidence rates among the 21 GBD regions, and cholelithiasis and alcohol consumption may be the main risk factors for the high burden of pancreatitis in Europe [18].

Pancreatitis, especially chronic pancreatitis, has traditionally been regarded as a disease of men, and it has been reported that the frequency is five times higher in men than in women because of the higher intake of alcohol and smoking in men [4, 19, 20]. In contrast, some studies have indicated that the prevalence in females may be higher than that in males [21, 22]. A systematic review reported by Xiao et al. [2] indicated that the incidence of chronic pancreatitis was two times higher in men than in women, whereas there was no difference in acute pancreatitis between men and women. In addition, no statistically significant difference was found between women and men in terms of incidence, prevalence, and YLDs in our study. This result was consistent with that of a population-based study [23] that reported a difference in etiologies of chronic pancreatitis between the two sexes; smoking and alcohol consumption were more common in men, whereas gallstones, autoimmune diseases, endoscopic retrograde cholangiopancreatography (ERCP), and idiopathic causes were more common in women [3, 4]. The distribution of age and sex varied greatly based on etiology. In GBD 2017, the highest burden of pancreatitis among females and males occurred in the 60–64 years and 45–49 years age groups, respectively. Similar to our results, previous studies also reported that both acute and chronic pancreatitis were more common in middle-aged and older people [5, 23, 24]. This implies that more policies should focus on these specific age groups globally. A previous study estimated that more than half of pancreatitis cases could be prevented if there were no smokers in the general population, nearly 1/4 of cases if there was a normal weight (body mass index [BMI] 18–25 kg/m^2^) in all people in the general population, and nearly 1/5 of cases if there was no alcohol consumption in all individuals [25]. Therefore, policies on how to tackle alcohol consumption, smoking, and weight should be prioritized for these age groups. Although the rate of pediatric pancreatitis has increased in recent years, it is uncommon among people younger than 20 years of age [26]; this was confirmed in our study because the burden in younger people was much lower than that in the middle-aged group. However, this does not mean that pediatric pancreatitis does not need more attention, and additional measures to prevent pediatric pancreatitis are warranted.

The development level of regions and countries is an important factor associated with pancreatitis burden that has not been compared in previous studies [2, 3, 5], and this study produced some important findings. First, positive correlations between YLDs and the SDIs for the 21 GBD regions and 195 countries and territories for pancreatitis from 1990 to 2017 were observed. This means that the burden of pancreatitis was generally higher in countries with higher socioeconomic development levels. Dietary habits, low alcohol consumption, and smoking rates due to shortages of alcohol and tobacco, and high physical exercise levels may be the reasons for the lower burden of pancreatitis in lower SDI countries. Alternatively, this phenomenon could also possibly be attributed to increased levels of physical inactivity, high BMI, and aging in higher SDI countries. However, the high burden of pancreatitis was not constrained to high or low SDI regions and countries, suggesting that pancreatitis is not a health problem exclusive to high income countries. The burden of pancreatitis was higher than the expected levels in some regions and countries, including Central Europe, central sub-Saharan African, high-income Asia Pacific, Tropic Latin American, Central Asia, and countries and territories such as Slovakia, Belgium, and Poland. At the global level, a positive correlation between the YLDs and the SDI level over the past 28 years was observed. However, the global burden of pancreatitis has been lower than the expected level in recent years. Early diagnosis, improved supportive care, and clarity on the optimal timing to conduct effective interventions (surgery, endoscopic, or percutaneous drainage) may contribute to decreasing the pancreatitis burden globally [3, 6]. Second, when estimating the burden of pancreatitis, the observed values and the expected values based on the SDI in each region and country should be combined when considering prevention programs.

Focusing on risk factors has been an important approach in prevention programs. The risk factors for pancreatitis include demographic and socioeconomic factors, race, gallstones, alcohol consumption, tobacco smoking, obesity, autoimmune diseases, genetic or metabolic causes, obstructive causes, and so on [3, 4, 27, 28]. However, some risk factors have not been identified. A previous study reported that the prevalence of pancreatitis was approximately 4 times higher in alcohol-consuming people than in people who did not consume alcohol [28]. Therefore, alcohol consumption, as one of the most important risk factors, should be carefully regulated, and specific prevention measures should be applied by policy makers.

To the best of our knowledge, the present study is the first to comprehensively analyze the relative burden of pancreatitis at the global, regional, and national levels between 1990 and 2017, but several limitations should be considered. First, the quality and quantity of the input data used in the DisMod-MR 2.1 model may influence the accuracy and robustness of the GBD 2017 estimates. As data are absent and sparse in many regions and countries and only a few countries or territories provided actual national data across the world, the burden estimates are heavily dependent on the modeled data instead of typical data from individual population-based studies. Therefore, national-level burden should be interpreted carefully. If possible, more health surveys at the national level are encouraged to acquire more details and representative data from each country. Second, the effects of prevention and management strategies in different regions or countries were not considered, and substantial variations might be found between low- to middle-income countries and high-income countries. Third, in the data estimated from GBD 2017, acute and chronic pancreatitis data were combined, with no differentiation between pancreatitis subtypes in this study. To clarify the true burden of pancreatitis, clear stratification of pancreatitis types, especially according to histology, is recommended in the future.

## Conclusions

Pancreatitis is a major public health issue worldwide, but there is geographical variation in the burden of pancreatitis. Globally, the age-standardized prevalence and YLDs rates increased from 1990 to 2017; however, the age-standardized incidence rate decreased. The highest burden of pancreatitis was observed in middle-aged patients, and no statistically significant difference was found between males and females. Improved awareness of pancreatitis, its risk factors, and the importance of early detection and treatment are warranted to reduce the future burden of this condition. Improving pancreatitis health data in all regions and countries, the monitoring of the pancreatitis burden and the treatment of pancreatitis are strongly recommended.

## Supplementary information

**Additional file 1: Figure 1.** GBD 2017 DisMod-MR 2.1 analytical cascade. **Figure 2.** The flowcharts of estimation for acute pancreatitis and chronic pancreatitis. **Table S1.** Betas and exponentiated values (which can be interpreted as odds ratio) of study-level covariates and location-level covariates of acute pancreatitis. **Table S2.** Betas and exponentiated values (which can be interpreted as odds ratio) of study-level covariates and location-level covariates of chronic pancreatitis. **Table S3.** Sequelae for pancreatitis and associated disability weights from GBD 2017. GBD = Global Burden of Diseases, Injuries, and Risk Factors Study. **Table S4.** Prevalent cases of pancreatitis in 1990 and 2017 for both sexes and percentage change of age-standardized rates (ASR) by location. **Table S5.** Incident cases of pancreatitis in 1990 and 2017 for both sexes and percentage change of age-standardized rates (ASR) by location. **Table S6.** YLDs of pancreatitis in 1990 and 2017 for both sexes and percentage change of age-standardized rates (ASR) by location.

**Additional file 2: Figure S1.** The percentage change in age-standardized point prevalence of pancreatitis from 1990 to 2017 for 21 Global Burden of Disease regions by sex. **Figure S2.** Number of prevalent cases of pancreatitis from 1990 to 2017 for 21 Global Burden of Disease regions. **Figure S3.** The percentage change in age-standardized point incidence of pancreatitis from 1990 to 2017 for 21 Global Burden of Disease regions by sex. **Figure S4.** Number of incident cases of pancreatitis from 1990 to 2017 for 21 Global Burden of Disease regions. **Figure S5.** The age-standardized YLDs of pancreatitis in 2017 for 21 GBD regions, by sex. **Figure S6.** The percentage change in age-standardized point YLDs of pancreatitis from 1990 to 2017 for 21 Global Burden of Disease regions by sex. **Figure S7.** Age-standardized YLDs rates of pancreatitis per 100,000 population in 2017, by country and territory. **Figure S8.** Global cases and age-standardized rates of incidence of pancreatitis per 100,000 population by age and sex, 2017. Shading indicates the upper and lower limits of the 95% uncertainty intervals (95% UIs). **Figure S9.** Global cases and age-standardized rates of YLDs of pancreatitis per 100,000 population by age and sex, 2017. Shading indicates the upper and lower limits of the 95% uncertainty intervals (95% UIs). **Figure S10.** Age-standardized incidence rates for pancreatitis for 195 countries and territories by SDI,2017. Expected values based on Socio-demographic Index and disease rates in all locations are shown as the black line. SDI = Sociodemographic Index. **Figure S11.** Age-standardized prevalence rates for pancreatitis for 195 countries and territories by SDI,2017. Expected values based on Socio-demographic Index and disease rates in all locations are shown as the black line. SDI = Sociodemographic Index.

## Data Availability

The datasets generated for this study can be found in the GBD at http://ghdx.healthdata.org/gbd-results-tool.

## Abbreviations

ASR: Age-standardized rate
BMI: Body mass index
CSMR: Cause-specific mortality rate
DALYs: Disability-adjusted life year
DWs: Disability weights
ERCP: Endoscopic retrograde cholangiopancreatography
GATHER: Guidelines for Accurate and Transparent Health Estimates Reporting
GBD: Global Burden of Diseases, Injuries, and Risk Factors Study
ICD: International Classification of Diseases
IHME: Institute for Health Metrics and Evaluation
LDI: Lag-distributed income per capita
MEPS: Medical Expenditure Panel Surveys
SDI: Sociodemographic index
UIs: Uncertainty intervals
YLDs: Years lived with disability
YLLs: Years of life lost

## References

[1] Hall TC, Garcea G, Webb MA, Al-Leswas D, Metcalfe MS, Dennison AR (2014). The socio-economic impact of chronic pancreatitis: a systematic review. J Eval Clin Pract.

[2] Xiao AY, Tan ML, Wu LM (2016). Global incidence and mortality of pancreatic diseases: a systematic review, meta-analysis, and meta-regression of population-based cohort studies. Lancet Gastroenterol Hepatol.

[3] Yadav D, Lowenfels AB (2013). The epidemiology of pancreatitis and pancreatic cancer. Gastroenterology..

[4] Kleeff J, Whitcomb DC, Shimosegawa T (2017). Chronic pancreatitis. Nat Rev Dis Primers.

[5] Petrov MS, Yadav D (2019). Global epidemiology and holistic prevention of pancreatitis. Nat Rev Gastroenterol Hepatol.

[6] Uc A, Husain SZ (2019). Pancreatitis in children. Gastroenterology..

[7] Global Health Estimates 2016 (2018). Global Health Estimates 2016: disease burden by cause, age, sex, by country and by region, 2000–2016.

[8] GBD 2017 Causes of Death Collaborators (2018). Global, regional, and national incidence, prevalence, and years lived with disability for 354 diseases and injuries for 195 countries and territories, 1990–2017: a systematic analysis for the Global Burden of Disease Study 2017. Lancet.

[9] GBD 2017 Causes of Death Collaborators (2018). Global, regional, and national age-sex-specific mortality for 282 causes of death in 195 countries and territories, 1980–2017: a systematic analysis for the Global Burden of Disease Study 2017. Lancet.

[10] GBD 2017 Risk Factor Collaborators (2018). Global, regional, and national comparative risk assessment of 84 behavioural, environmental and occupational, and metabolic risks or clusters of risks for 195 countries and territories, 1990–2017: a systematic analysis for the Global Burden of Disease Study 2017. Lancet.

[11] GBD 2017 Risk Factor Collaborators (2018). Global, regional, and national age-sex-specific mortality and life expectancy, 1950–2017: a systematic analysis for the Global Burden of Disease Study 2017. Lancet.

[12] Stevens GA, Alkema L, Black RE (2016). Guidelines for accurate and transparent health estimates reporting: the GATHER statement. Lancet..

[13] GBD 2016 Disease and Injury Incidence and Prevalence Collaborators (2017). Global, regional, and national incidence, prevalence, and years lived with disability for 328 diseases and injuries for 195 countries, 1990–2016: a systematic analysis for the Global Burden of Disease Study 2016. Lancet.

[14] Pourshams A, Sepanlou SG, Ikuta KS (2019). The global, regional, and national burden of pancreatic cancer and its attributable risk factors in 195 countries and territories, 1990–2017: a systematic analysis for the Global Burden of Disease Study 2017. Lancet Gastroenterol Hepatol.

[15] Wang Y. Smoothing splines: methods and applications. Chapman and Hall/CRC; 2011.

[16] Ginestet C. ggplot2: Elegant Graphics for Data Analysis. J Royal Statist Soci: Series A. 2011;174(1):245–6.

[17] Global Burden of Disease Study 2013 Collaborators (2015). Global, regional, and national incidence, prevalence, and years lived with disability for 301 acute and chronic diseases and injuries in 188 countries, 1990–2013: a systematic analysis for the Global Burden of Disease Study 2013. Lancet.

[18] Gullo L, Migliori M, Oláh A (2002). Acute pancreatitis in five European countries: etiology and mortality. Pancreas..

[19] Lévy P, Barthet M, Mollard BR, Amouretti M, Marion-Audibert A-M, Dyard F (2006). Estimation of the prevalence and incidence of chronic pancreatitis and its complications. Gastroenterol Clin Biol.

[20] Hirota M, Shimosegawa T, Masamune A (2014). The seventh nationwide epidemiological survey for chronic pancreatitis in Japan: clinical significance of smoking habit in Japanese patients. Pancreatology..

[21] Coté GA, Yadav D, Slivka A (2011). Alcohol and smoking as risk factors in an epidemiology study of patients with chronic pancreatitis. Clin Gastroenterol Hepatol.

[22] Frulloni L, Gabbrielli A, Pezzilli R (2009). Chronic pancreatitis: report from a multicenter Italian survey (PanCroInfAISP) on 893 patients. Dig Liver Dis.

[23] Yadav D, Timmons L, Benson JT, Dierkhising RA (2011). Chari STJAJoG. Incidence, prevalence, and survival of chronic pancreatitis: a population-based study. Am J Gastroenterol.

[24] Pendharkar SA, Mathew J, Petrov MS (2017). Age- and sex-specific prevalence of diabetes associated with diseases of the exocrine pancreas: a population-based study. Dig Liver Dis.

[25] Alsamarrai A, Das SL, Windsor JA, Petrov MS (2014). Factors that affect risk for pancreatic disease in the general population: a systematic review and meta-analysis of prospective cohort studies. Clin Gastroenterol Hepatol.

[26] Morinville VD, Barmada MM, Lowe MEJP (2010). Increasing incidence of acute pancreatitis at an American pediatric tertiary care center: is greater awareness among physicians responsible?. Pancreas..

[27] Machicado JD, Yadav D (2017). Epidemiology of recurrent acute and chronic pancreatitis: similarities and differences. Dig Dis Sci.

[28] Kumar S, Ooi CY, Werlin S (2016). Risk factors associated with pediatric acute recurrent and chronic pancreatitis: lessons from INSPPIRE. JAMA Pediatr.

