# Supplementary material for: The global, regional, and national burden of pancreatitis in 195 countries and territories, 1990–2017: a systematic analysis for the Global Burden of Disease Study 2017
Source: BMC Med. 2020 Dec 10;18:388. doi: 10.1186/s12916-020-01859-5 (PMC7726906; doi:10.1186/s12916-020-01859-5)
Supplement: Supplementary file 1 — Additional file 1: Figure 1. GBD 2017 DisMod-MR 2.1 analytical cascade. Figure 2. The flowcharts of estimation for acute pancreatitis and chronic pancreatitis. Table S1. Betas and exponentiated values (which can be interpreted as odds ratio) of study-level covariates and location-level covariates of acute pancreatitis. Table S2. Betas and exponentiated values (which can be interpreted as odds ratio) of study-level covariates and location-level covariates of chronic pancreatitis. Table S3. Sequelae for pancreatitis and associated disability weights from GBD 2017. GBD = Global Burden of Diseases, Injuries, and Risk Factors Study. Table S4. Prevalent cases of pancreatitis in 1990 and 2017 for both sexes and percentage change of age-standardized rates (ASR) by location. Table S5. Incident cases of pancreatitis in 1990 and 2017 for both sexes and percentage change of age-standardized rates (ASR) by location. Table S6. YLDs of pancreatitis in 1990 and 2017 for both sexes and percentage change of age-standardized rates (ASR) by location. [file 12916_2020_1859_MOESM1_ESM.docx]

**Additional file 1**

**The global, regional, and national burden of pancreatitis in 195 countries and territories, 1990–2017: a systematic analysis for the Global Burden of Disease Study 2017**

Section 1. Data adjustment …………………………………………………………...….2-3

Section 2. DisMod-MR 2.1 estimation …………………………………………….....….3-7

2.1. DisMod-MR 2.1 analytical process……………………………………….3-4

2.2. DisMod-MR 2.1 likelihood estimation…………………………………...4-7

**List of supplemental Table**

Table S1: Betas and exponentiated values (which can be interpreted as odds ratio) of study-level covariates and location-level covariates of acute pancreatitis……………………... 8

Table S2: Betas and exponentiated values (which can be interpreted as odds ratio) of study-level covariates and location-level covariates of chronic pancreatitis……………………8-9

Table S3: Sequelae for pancreatitis and associated disability weights from GBD 2017…9

Table S4: Prevalent cases of pancreatitis in 1990 and 2017 for both sexes and percentage change of age-standardised rates (ASR) by location………………………………….......10-27

Table S5: Incident cases of pancreatitis in 1990 and 2017 for both sexes and percentage change of age-standardised rates (ASR) by location……………………………………...27-41

Table S6: YLDs of pancreatitis in 1990 and 2017 for both sexes and percentage change of age-standardised rates (ASR) by location………………………………………………...41-51

Section 1. Data adjustment

In order to make the data more consistent and suitable for modelling, Institute for Health Metrics and Evaluation (IHME) corrected the claims and hospital data, used a number of other adjustments to extracted nonfatal sources. In the second step of nonfatal estimation, commonly applied adjustments included age-sex splitting, bias correction, adjustments for underreporting of notification data, and computing expected values of excess mortality. Age-sex splitting was commonly utilized to literature data reported by age or sex but not by age and sex. For Global Burden of Diseases, Injuries, and Risk Factors Study(GBD) 2017, IHME split all data reported in age groups with a width greater than 20 years, using age patterns from available survey microdata or regional patterns derived from an initial run of main modelling tool, DisMod-MR 2.1. For most of the bias correction of data for variations in study attributes, the meta-regression component of DisMod-MR 2.1 was applied, such as case definitions and measurement method. DisMod-MR 2.1 calculates a single adjustment that is applied regardless of age, sex, or location. IHME applied bias corrections to the data before entry into DisMod-MR 2.1, if enough data were available to differentiate these adjustments by age, sex, or location, or if detailed survey data were available to make more precise adjustments between different thresholds on a biochemical measure. Age-specific correction factors were derived, due to the relationship varied with age. The correction of notification data for underreporting relied on studies that had examined the gap between true incidence and notified cases.

In GBD 2017, IHME estimated expected values of excess mortality from prevalence or incidence and cause-specific mortality rate (CSMR) data for every cause for which deaths were estimated apart from a few causes with very low mortality rates such as uterine fibroids. IHME matched every prevalence data point (or incidence data for short-duration conditions) with the CSMR value corresponding to the age range, sex, year, and location of the data point. IHME restricted this to data points reporting age-groups spanning 20 years or less. The ratio of CSMR to prevalence (or incidence times a short duration) is conceptually equivalent to an excess mortality rate. To reflect a gradient in excess mortality, we added in all relevant models the log of lag distributed income (LDI) or the Healthcare Access and Quality (HAQ) index as a covariate, with a strong prior that as LDI or HAQ Index increases, excess mortality declines [8]

Section 2. DisMod-MR 2.1 estimation

**2.1. DisMod-MR 2.1 analytical process**

Figure 1 shows the DisMod-MR 2.1 analytical process. IHME divided the sequence of estimation occurs into five levels: global, super-region, region, country and, where applicable, subnational location. The super-region priors are generated at the global level with mixed-effects, nonlinear regression using all available data; the super-region fit, in turn, informs the region fit, and so on down the cascade. The wrapper gives analysts the choice to branch the cascade in terms of time and sex at different levels depending on data density. The default used in most models is to branch by sex after the global fit but to retain all years of data until the lowest level in the cascade.

**2.2. DisMod-MR 2.1 likelihood estimation**

The Gaussian, log-Gaussian, Laplace or Log-Laplace likelihood function in DisMod-MR 2.1 was used to analyzed. The default log-Gaussian equation for the data

likelihood is:

$$-\log\left[ p\left( y_{j} | \Phi\right) \right]=\log\left( \sqrt{2\pi} \right)+\log\left( \delta_{j}+s_{j} \right)+\frac{1}{2\left( \frac{\log\left( a_{j}+\eta_{j} \right)-\log\left( m_{j}+\eta_{j} \right)}{\delta_{j}+s_{j}} \right)^{2}}$$

where, y_j_ is a ‘measurement value’ (i.e., data point);$\Phi$ denotes all model random variables; $\eta_{j}$ is the offset value, eta, for particular ‘integrand’ (prevalence, incidence, remission, excess mortality rate, with-condition mortality rate, cause-specific mortality rate, relative risk or standardized mortality ratio) and $a_{j}$ is the adjusted measurement for data point j, defined by:

$$a_{j}=e^{\left( -u_{j}-c_{j} \right)}y_{j}$$

where u_j_ is the total ‘area effect’ (i.e., the sum of the random effects at three levels of the cascade: super-region, region and country) and c_j_ is the total covariate effect (i.e., the mean combined fixed effects for sex, study covariates, and country level covariates), defined by:

$$c_{j}=\sum_{k=0}^{K\left[ I\left( j \right) \right]-1} \beta_{I\left( j \right),k}{\overset{{}}{X}}_{k,j}$$

with standard deviation

$$s_{j}=\sum_{l=0}^{L\left[ I\left( j \right) \right]-1} \zeta_{I\left( j \right),l}{\overset{{}}{Z}}_{k,j}$$

where k denotes the mean value of each data point in relation to a covariate (also called x-covariate); I(j) denotes a data point for a particular integrand, j; $\beta_{I\left( j \right),k}$ is the multiplier of the k^th^ x-covariate for the i^th^ integrand; ${\overset{{}}{X}}_{k,j}$ is the covariate value corresponding to the data point j for covariate k; l denotes the standard deviation of each data point in relation to a covariate (also called z-covariate); $\zeta_{I(j),l}$ is the multiplier of the I^th^ z-covariate for the i^th^ integrand; and$\delta_{j}$ is the standard deviation for adjusted measurement j, defined by:

$$\delta_{j}=\log\left[ y_{j}+e^{\left( -u_{j}-c_{j} \right)}\eta_{j}+c_{j} \right]-\log\left[ y_{j}+e^{\left( -u_{j}-c_{j} \right)}\eta_{j} \right]$$

Where m_j_ denotes the model for the j^th^ measurement, not counting effects or measurement noise and defined by:

$$m_{j}=\frac{1}{B\left( j \right)-A\left( j \right)}\int_{A\left( j \right)}^{B\left( j \right)} I_{j}\left( a \right)da$$

where A(j) is the lower bound of the age range for a data point; B(j) is the upper bound of the age range for a data point; and Ij denotes the function of age corresponding to the integrand for data point j [8].

**
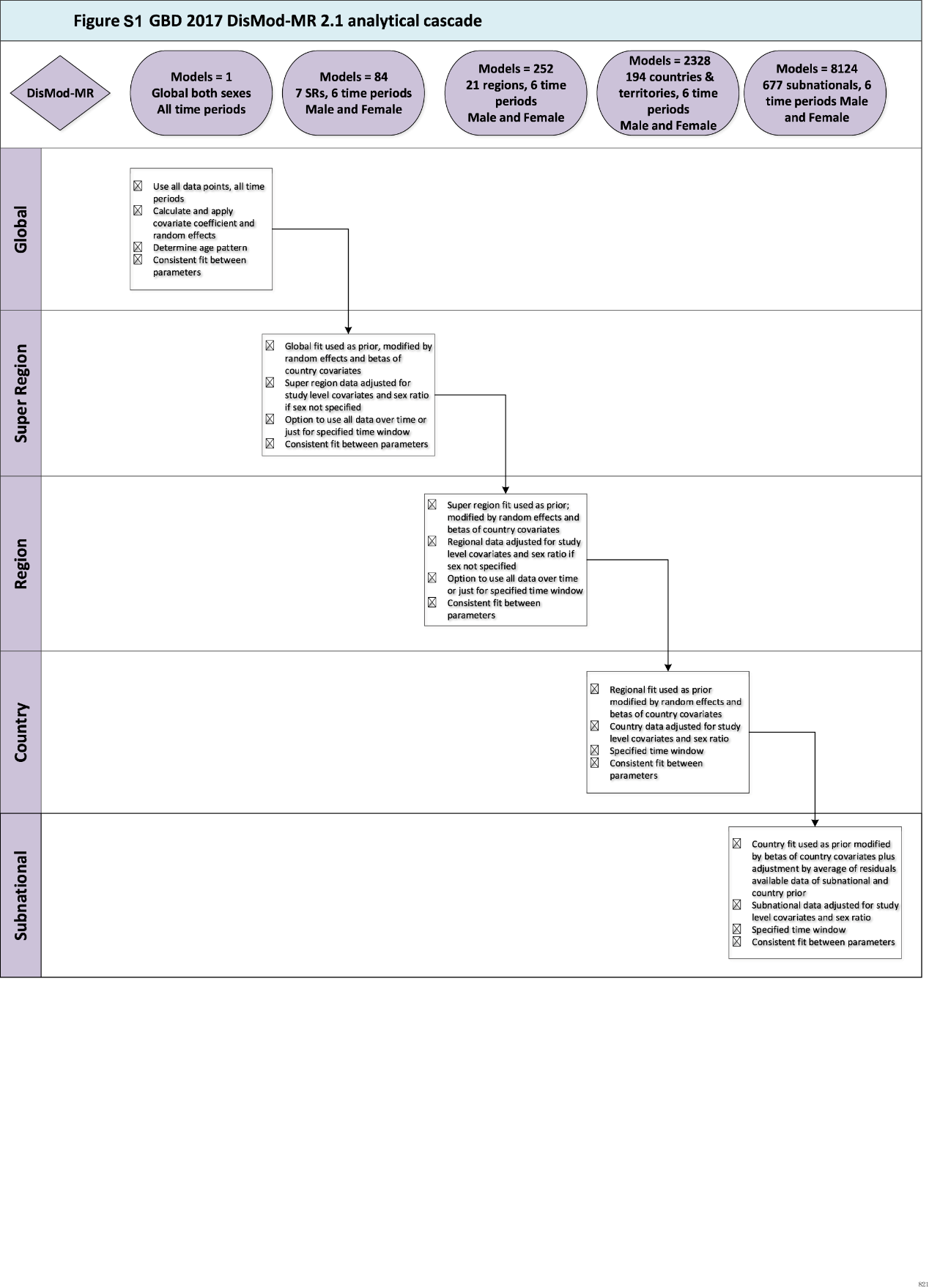
Figure 1:** GBD 2017 DisMod-MR 2.1 analytical cascade [8]


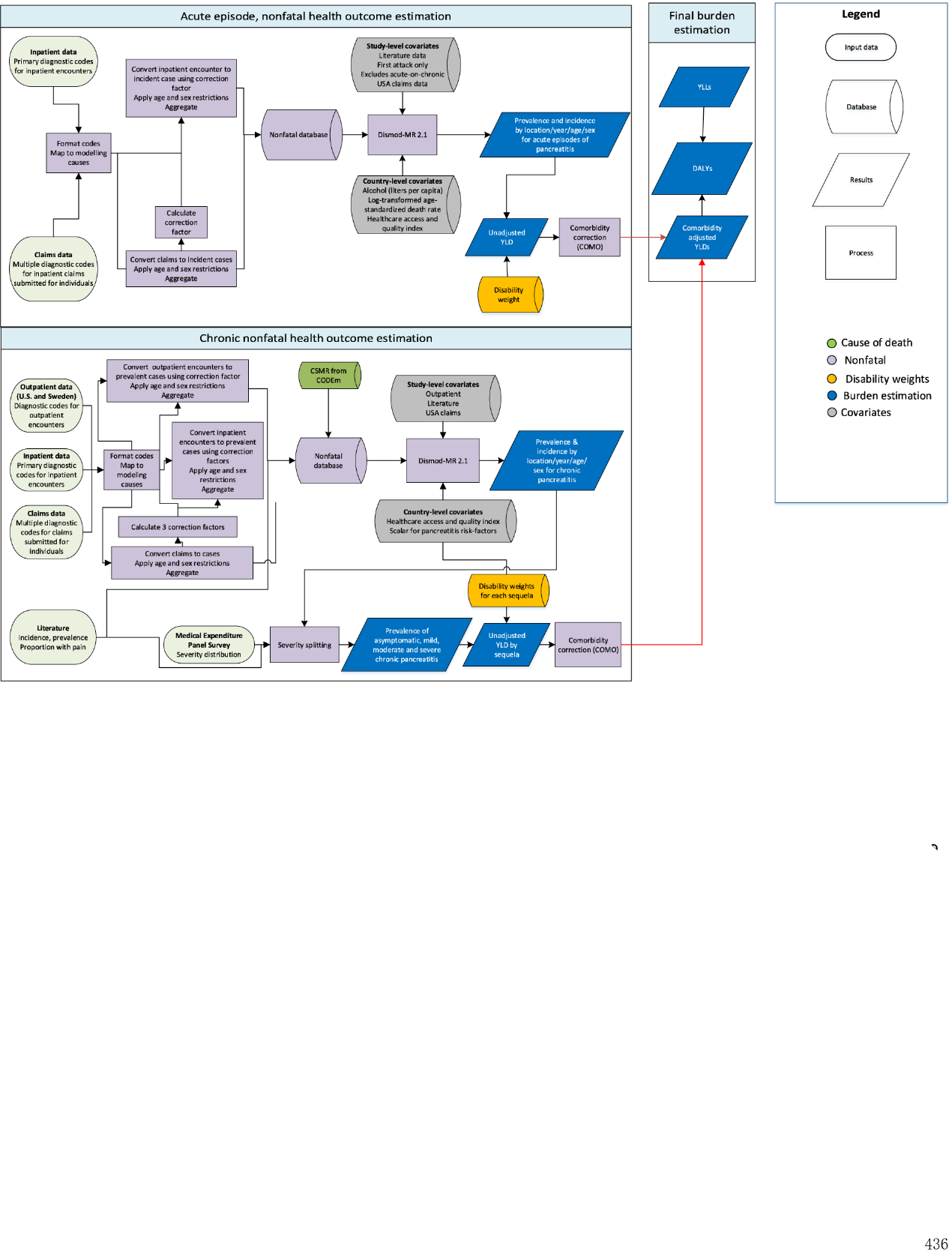


**Figure 2:** The flowcharts of estimation for acute pancreatitis and chronic pancreatitis [8]

Table S1: Betas and exponentiated values (which can be interpreted as odds ratio) of study-level covariates and location-level covariates of acute pancreatitis [8]

| **Covariate type** | **Covariate** | **Parameter** | **beta** | **Exponentiated beta** |
| --- | --- | --- | --- | --- |
| Study-level covariate | Literature | Incidence | -0.096 (-0.32 to 0.17) | 0.91 (0.72 to 1.18) |
| Study-level covariate | First attack only | Incidence | 0.075 (-0.33 to 0.51) | 1.08 (0.72 to 1.66) |
| Study-level covariate | Excludes acute on-chronic | Incidence | 1.73 (1.25 to 2.00) | 5.65 (3.49 to 7.36) |
| Study-level covariate | USA claims data | Incidence | 0.23 (0.21 to 0.25) | 1.26 (1.24 to 1.29) |
| Location covariate | Alcohol (litres per capita) | Incidence | 0.00015 (0.0000090 to 0.00051) | 1.00 (1.00 to 1.00) |
| Location covariate | Natural log of age-standardised death rate due to pancreatitis | Incidence | 0.39 (0.36 to 0.42) | 1.47 (1.44 to 1.52) |
| Location covariate | Healthcare Access and Quality index | Excess mortality rate | -0.0069 (-2 to 2) | 0.99 (0.14 to 7.39) |

**Table S2:** Betas and exponentiated values (which can be interpreted as odds ratio) of study-level covariates and location-level covariates of chronic pancreatitis [8]

| **Covariate type** | **Covariate** | **Parameter** | **beta** | **Exponentiated beta** |
| --- | --- | --- | --- | --- |
| Study-level covariate | Outpatient | Prevalence | -3.41 (-3.47 to -3.35) | 0.033 (0.031 to 0.035) |
| Study-level covariate | Literature | Prevalence | -1.7 (-1.98 to -1.32) | 0.18 (0.14 to 0.27) |
| Study-level covariate | USA claims | Prevalence | -0.65 (-0.68 to -0.61) | 0.52 (0.51 to 0.54) |
| Location covariate | Log-transformed age standardised scaled exposure variable for pancreatitis risk factors | Prevalence | -0.11 (-0.15 to -0.076) | 0.89 (0.86 to 0.93) |
| Location covariate | Healthcare Access and Quality index | Excess mortality rate | -0.038 (-0.039 to -0.038) | 0.96 (0.96 to 0.96) |

Table S3: Sequelae for pancreatitis and associated disability weights from GBD 2017[8]

| Sequela | Lay description | Disability weight (95% UI) |
| --- | --- | --- |
| Acute pancreatitis episodes | This person has severe pain in the belly and feels nauseated. The person has high fevers, pain and feels very weak. This causes great difficulty with daily activities. | *Combined DW: 0.324 (0.220- 0.442) 0.133 (0.088 - 0.190) |
| Asymptomatic chronic  pancreatitis | -- | 0 |
| Mild chronic pancreatitis | This person has some pain in the belly that causes nausea but does not interfere with daily activities. | 0.011 (0.005 - 0.021) |
| Moderate chronic pancreatitis | This person has pain in the belly and feels nauseous. The person has difficulties with daily activities. | 0.114 (0.080 - 0.159) |
| Severe chronic pancreatitis | This person has severe pain in the belly and feels  nauseated. The person is anxious and unable to  carry out daily activities. | 0.324 (0.219 - 0.442) |

GBD=Global Burden of Diseases, Injuries, and Risk Factors Study

| Table S4: Prevalent cases of pancreatitis in 1990 and 2017 for both sexes and percentage change of age-standardised rates (ASR) by location | | | | | |  |
| --- | --- | --- | --- | --- | --- | --- |
|  | 1990 |  | 2017 |  | Percentage change in ASR from 1990 and 2017 |  |
|  | Counts (95% UI) | Rate (95% UI) | Counts (95% UI) | Rate (95% UI) |  |  |
| Global | 3038787(2768128 to 3307165) | 67.2(61.3 to 73) | 6115833(5533925 to 6704070) | 76.1(68.9 to 83.4) | 13.3(10.3 to 16.4) |  |
| High-income North America | 281428(254954 to 305489) | 86.9(78.7 to 94.4) | 445619(404471 to 484509) | 93(84.6 to 100.6) | 7(2.5 to 11.4) |  |
| Canada | 24332(21891 to 26611) | 77.7(69.8 to 85) | 47397(42322 to 52283) | 90.3(80.7 to 99.1) | 16.1(11.7 to 21) |  |
| Greenland | 28(26 to 31) | 58.3(53 to 63.6) | 46(42 to 51) | 71.4(64.1 to 78.1) | 22.4(17.8 to 26.9) |  |
| United States | 257062(232651 to 278730) | 87.8(79.6 to 95.5) | 398168(361388 to 432965) | 93.2(85 to 100.9) | 6.1(1.4 to 10.7) |  |
| Australasia | 11592(10431 to 12793) | 50.9(46 to 56.3) | 25152(22429 to 27862) | 64.3(57.3 to 71.7) | 26.1(21.2 to 30.8) |  |
| Australia | 8939(8053 to 9872) | 47(42.7 to 51.9) | 19871(17633 to 22114) | 60.2(53.3 to 67.5) | 28(22.2 to 33.2) |  |
| New Zealand | 2653(2379 to 2936) | 70.6(63.2 to 78.2) | 5281(4770 to 5765) | 86.3(77.7 to 94.3) | 22.1(17.1 to 27) |  |
| High-income Asia Pacific | 297236(270346 to 326181) | 149.7(136.4 to 164.2) | 424150(383580 to 470105) | 154.4(139.8 to 171) | 3.1(-1.3 to 6.9) |  |
| Brunei | 224(202 to 250) | 114.4(103.8 to 126.7) | 567(509 to 634) | 132.3(118.7 to 147.4) | 15.7(10.9 to 20.9) |  |
| Japan | 235009(213089 to 258344) | 152.8(139.2 to 167.7) | 303997(276270 to 334338) | 157.7(143.6 to 173.4) | 3.2(-1.1 to 7.3) |  |
| Singapore | 3751(3400 to 4147) | 121.9(110.4 to 134.4) | 9209(8241 to 10257) | 136.4(122 to 151.6) | 11.9(6 to 18.2) |  |
| South Korea | 58252(53041 to 64128) | 143.2(130.8 to 157.7) | 110376(97545 to 124070) | 150(133.7 to 168.6) | 4.7(-2.2 to 10.9) |  |
| Western Europe | 429060(389971 to 471303) | 87.9(79.9 to 96.7) | 716616(644467 to 791137) | 111.8(100.5 to 124.4) | 27.3(21.9 to 32.4) |  |
| Andorra | 48(43 to 54) | 77.4(68.7 to 86.7) | 120(106 to 136) | 100.1(88.8 to 113.2) | 29.3(23.6 to 35.1) |  |
| Austria | 18970(17200 to 20665) | 193.7(176 to 211.2) | 21971(19843 to 24321) | 168.1(151.8 to 186.9) | -13.2(-19.2 to -6.8) |  |
| Belgium | 22191(19995 to 24669) | 174.3(157.2 to 194.3) | 43362(38579 to 48594) | 274.3(242.6 to 306.5) | 57.4(46.6 to 69.3) |  |
| Cyprus | 254(227 to 284) | 30.8(27.5 to 34.3) | 702(625 to 786) | 41.6(37.1 to 46.6) | 35.2(30.1 to 39.9) |  |
| Denmark | 6426(5858 to 7045) | 96.2(87.8 to 105.4) | 11500(10341 to 12734) | 139(125.4 to 154.9) | 44.5(36.6 to 52.7) |  |
| Finland | 9747(8881 to 10707) | 154(140.4 to 169.3) | 17573(15900 to 19481) | 225.6(204.2 to 250.4) | 46.5(36.1 to 58.2) |  |
| France | 53732(48982 to 59424) | 76.5(69.7 to 84.7) | 86983(77480 to 96918) | 92.7(82.7 to 103.7) | 21.2(13.2 to 29.6) |  |
| Germany | 83186(75695 to 91553) | 78.1(71.1 to 86.1) | 142815(128277 to 157981) | 110.7(99.6 to 122.6) | 41.7(32.9 to 51.3) |  |
| Greece | 9487(8484 to 10574) | 71.3(63.8 to 79.6) | 16238(14465 to 18168) | 99(87.9 to 112.2) | 38.8(32.5 to 46.1) |  |
| Iceland | 115(104 to 127) | 42.9(38.6 to 47.3) | 308(275 to 344) | 70.6(63.1 to 79.3) | 64.8(57.2 to 72.9) |  |
| Ireland | 2336(2097 to 2601) | 62.3(55.7 to 69.7) | 5626(4965 to 6327) | 90(79.4 to 101.2) | 44.5(37.5 to 52) |  |
| Israel | 3041(2715 to 3413) | 64.3(57.4 to 72.2) | 9187(8162 to 10355) | 94.2(83.5 to 106.6) | 46.4(40 to 53.6) |  |
| Italy | 50836(45749 to 56134) | 67.7(61 to 74.9) | 74265(66658 to 81929) | 74.9(67.1 to 82.7) | 10.6(4.1 to 17) |  |
| Luxembourg | 425(380 to 472) | 85.8(77 to 95.3) | 863(771 to 963) | 105(94 to 116.9) | 22.5(14.6 to 29.4) |  |
| Malta | 147(132 to 164) | 34.5(31.1 to 38.4) | 284(254 to 316) | 42.8(38.2 to 48) | 24.1(18.5 to 29.6) |  |
| Netherlands | 11005(9820 to 12374) | 60.5(54.1 to 68.1) | 19759(17388 to 22405) | 80.3(70.3 to 91) | 32.8(23.6 to 42.9) |  |
| Norway | 8228(7389 to 9116) | 155.9(139.8 to 172.9) | 13249(11903 to 14669) | 180.4(162.1 to 199.1) | 15.7(7.9 to 23.4) |  |
| Portugal | 9475(8610 to 10449) | 77.6(70.7 to 85.7) | 16319(14483 to 18289) | 97.4(86.9 to 109.3) | 25.5(17 to 33.6) |  |
| Spain | 42001(38097 to 46068) | 87.9(79.7 to 96.7) | 76164(67410 to 84977) | 106.9(94.6 to 119.5) | 21.6(12.8 to 30.9) |  |
| Sweden | 8609(7793 to 9507) | 75.2(68 to 83.3) | 12938(11502 to 14448) | 89.7(79.5 to 101.2) | 19.4(13.3 to 25.9) |  |
| Switzerland | 4938(4411 to 5488) | 54.7(48.8 to 60.8) | 8440(7590 to 9413) | 65.4(58.5 to 73.1) | 19.6(13.5 to 26.1) |  |
| United Kingdom | 83448(75344 to 92043) | 114.1(102.8 to 126) | 137209(123978 to 151021) | 147.3(133.5 to 162.9) | 29.1(24.5 to 33.6) |  |
| Southern Latin America | 18146(16575 to 19876) | 37.9(34.7 to 41.5) | 30741(27476 to 34098) | 40.8(36.4 to 45.4) | 7.5(2.4 to 12.8) |  |
| Argentina | 12635(11543 to 13875) | 38.6(35.2 to 42.4) | 20240(18015 to 22608) | 41.1(36.6 to 45.9) | 6.6(1.4 to 12.4) |  |
| Chile | 4394(4003 to 4802) | 37.7(34.4 to 41.2) | 8814(7899 to 9803) | 40.4(36.2 to 44.7) | 6.9(0.1 to 13.7) |  |
| Uruguay | 1117(1013 to 1216) | 32(29 to 35) | 1686(1506 to 1870) | 39.3(34.9 to 43.8) | 22.9(17.5 to 28.7) |  |
| Eastern Europe | 370842(339703 to 403096) | 140.9(129.5 to 152.8) | 614456(558090 to 677548) | 213.8(194.5 to 235.3) | 51.8(46.5 to 57) |  |
| Belarus | 16685(15155 to 18277) | 137.8(125.7 to 150.6) | 25545(22909 to 28486) | 191.8(172.3 to 213.8) | 39.2(31.2 to 47.4) |  |
| Estonia | 2727(2489 to 2963) | 145.4(133.1 to 157.6) | 3409(3035 to 3790) | 178.5(159.8 to 198.3) | 22.8(15.3 to 29.8) |  |
| Latvia | 4942(4494 to 5390) | 150.9(137.5 to 164.3) | 5582(5011 to 6215) | 195.5(176 to 217.6) | 29.6(22.6 to 37.4) |  |
| Lithuania | 5928(5383 to 6507) | 138(125.6 to 151.2) | 8953(8052 to 9908) | 215.1(195.3 to 238.1) | 55.9(48.2 to 64.3) |  |
| Moldova | 10270(9520 to 11100) | 222.3(206.2 to 240.4) | 9245(8429 to 10173) | 187.1(171.2 to 206) | -15.8(-20.7 to -10.7) |  |
| Russian Federation | 249777(229690 to 272076) | 144(132.6 to 156.5) | 449934(408229 to 495540) | 226.9(206.1 to 249.7) | 57.5(52 to 63.1) |  |
| Ukraine | 80512(73504 to 88226) | 126.2(115.4 to 138) | 111787(101792 to 123138) | 181(165.5 to 198.9) | 43.5(36.9 to 50.1) |  |
| Central Europe | 263475(243193 to 285727) | 183(169.4 to 197.9) | 383787(352525 to 414563) | 222.1(205 to 239.8) | 21.4(16.8 to 26.3) |  |
| Albania | 3106(2792 to 3418) | 119.1(106.4 to 131.1) | 6118(5423 to 6831) | 168.3(149.8 to 187.8) | 41.3(34.7 to 48.2) |  |
| Bosnia and Herzegovina | 5773(5145 to 6382) | 125.2(112.5 to 138) | 9037(7974 to 10079) | 178.1(158.9 to 198.8) | 42.2(36.5 to 48.2) |  |
| Bulgaria | 14588(13208 to 16036) | 126.6(115.6 to 139) | 20102(17969 to 22280) | 177.3(159.2 to 197.2) | 40.1(33.1 to 46.6) |  |
| Croatia | 9241(8405 to 10134) | 149.7(137 to 163.7) | 9961(9057 to 10912) | 146(133 to 159.5) | -2.5(-9.2 to 3.5) |  |
| Czech Republic | 25649(23594 to 27959) | 200.9(185.5 to 218.3) | 38802(35216 to 42345) | 234.1(212.7 to 255.7) | 16.5(8.2 to 23.9) |  |
| Hungary | 27135(24995 to 29297) | 201.7(186 to 216.8) | 28190(25375 to 31130) | 185.9(167.5 to 204.8) | -7.9(-15.3 to 0) |  |
| Macedonia | 2563(2313 to 2824) | 126.7(114.4 to 139.4) | 5175(4608 to 5761) | 173.1(154.8 to 192.3) | 36.6(30 to 44.5) |  |
| Montenegro | 870(793 to 954) | 133.9(122.3 to 146.7) | 1431(1270 to 1598) | 166.5(148.6 to 185.3) | 24.3(18.1 to 30.6) |  |
| Poland | 94423(86672 to 103227) | 215.8(198.4 to 235.5) | 151275(140260 to 161580) | 266.7(248.2 to 284.4) | 23.6(16.6 to 30.7) |  |
| Romania | 44843(41601 to 48703) | 166.9(155 to 181.2) | 63140(57685 to 68849) | 212.8(194.8 to 231.9) | 27.5(21.1 to 34) |  |
| Serbia | 16524(15067 to 18149) | 145.6(132.6 to 159.4) | 22031(19884 to 24335) | 168.5(152.3 to 185.6) | 15.7(9.6 to 21.4) |  |
| Slovakia | 14796(13448 to 16257) | 254.5(231.8 to 279.5) | 23340(21317 to 25470) | 297.7(273.4 to 325.3) | 17(11 to 22.7) |  |
| Slovenia | 3964(3623 to 4303) | 165.7(151.8 to 179.4) | 5185(4669 to 5727) | 156.3(141.3 to 172.2) | -5.7(-12 to 0.9) |  |
| Central Asia | 62308(56869 to 68014) | 114.1(104.2 to 124.6) | 111530(99718 to 124267) | 129.5(115.9 to 143.9) | 13.5(8.8 to 17.9) |  |
| Armenia | 2693(2414 to 2971) | 87.3(78.2 to 96.2) | 4437(3934 to 4946) | 117.9(105 to 131.3) | 35.1(30.1 to 40.9) |  |
| Azerbaijan | 4951(4439 to 5460) | 82.3(73.5 to 90.8) | 12174(10744 to 13729) | 114.3(101.3 to 128.4) | 38.9(33.4 to 45.3) |  |
| Georgia | 5264(4690 to 5843) | 87.9(78.5 to 97.2) | 5557(4945 to 6194) | 112.9(100.9 to 125.6) | 28.4(23.6 to 33.5) |  |
| Kazakhstan | 27099(24771 to 29756) | 185(168.7 to 203.7) | 33701(30372 to 37613) | 181.8(164 to 202.8) | -1.7(-8 to 5.3) |  |
| Kyrgyzstan | 3070(2786 to 3353) | 88.4(80.1 to 97) | 5951(5349 to 6596) | 108.7(97.6 to 120.3) | 23(18.2 to 28) |  |
| Mongolia | 1711(1544 to 1892) | 126.1(113.6 to 140.4) | 4172(3674 to 4694) | 140.5(123.7 to 156.3) | 11.5(6.1 to 16.8) |  |
| Tajikistan | 3099(2775 to 3445) | 86.1(76.5 to 95.8) | 8259(7303 to 9324) | 116.5(103.1 to 130.9) | 35.2(30.5 to 40.2) |  |
| Turkmenistan | 2089(1884 to 2302) | 81.6(73.4 to 90.3) | 5182(4617 to 5811) | 112.7(100.8 to 125.5) | 38.1(33.1 to 43.6) |  |
| Uzbekistan | 12333(11156 to 13549) | 84.4(76.2 to 93.3) | 32096(28499 to 35950) | 113.3(100.9 to 126.2) | 34.2(28.8 to 39.5) |  |
| Central Latin America | 70367(64406 to 76877) | 64.4(58.8 to 69.9) | 188560(169971 to 206572) | 78.3(70.5 to 85.8) | 21.4(17 to 25.8) |  |
| Colombia | 13682(12432 to 15034) | 61.1(55.6 to 66.7) | 42534(38122 to 46639) | 79.7(71.3 to 87.4) | 30.4(23.2 to 37.8) |  |
| Costa Rica | 1702(1562 to 1848) | 80.9(73.9 to 88) | 4681(4199 to 5155) | 94.7(84.9 to 104.4) | 17(11.6 to 23) |  |
| El Salvador | 2183(1984 to 2373) | 60.8(55.3 to 66.3) | 4577(4117 to 5053) | 78.4(70.6 to 86.7) | 29(23.5 to 34.4) |  |
| Guatemala | 3405(3137 to 3699) | 68.3(62.9 to 73.9) | 10197(9254 to 11189) | 80.1(72.6 to 87.9) | 17.4(11.1 to 23.6) |  |
| Honduras | 1700(1557 to 1851) | 60.3(54.9 to 65.7) | 5674(5118 to 6244) | 82.2(74.1 to 90.5) | 36.3(30.6 to 42.6) |  |
| Mexico | 37957(34644 to 41453) | 66.9(61 to 72.7) | 91500(82437 to 100130) | 76.7(69.1 to 84) | 14.6(10.4 to 19) |  |
| Nicaragua | 1300(1179 to 1426) | 58.2(52.8 to 63.3) | 3905(3514 to 4285) | 75.6(68 to 83) | 30(25.1 to 35.5) |  |
| Panama | 1056(947 to 1160) | 59.1(53.3 to 64.8) | 3092(2765 to 3403) | 77.5(69.4 to 85.3) | 31.2(26.7 to 36.1) |  |
| Venezuela | 7382(6693 to 8120) | 58.3(52.8 to 63.8) | 22401(19972 to 24665) | 76.9(68.7 to 84.6) | 32(26.2 to 38) |  |
| Andean Latin America | 14076(12949 to 15221) | 54.7(50.2 to 59.2) | 39623(35598 to 43492) | 70.2(63.1 to 77.1) | 28.2(22.3 to 33.8) |  |
| Bolivia | 2008(1823 to 2198) | 49.4(44.8 to 54) | 6746(6037 to 7467) | 71.8(64.4 to 79.5) | 45.4(39.1 to 51.7) |  |
| Ecuador | 4563(4239 to 4899) | 69.2(64.2 to 74.3) | 10282(9305 to 11236) | 67.2(60.9 to 73.4) | -2.9(-8.8 to 3.4) |  |
| Peru | 7504(6859 to 8186) | 49.6(45.3 to 54.1) | 22595(20227 to 24964) | 71.1(63.5 to 78.5) | 43.2(35.5 to 51.1) |  |
| Caribbean | 17321(15656 to 18967) | 61.3(55.4 to 67.1) | 43323(38842 to 47732) | 86.4(77.4 to 95.1) | 40.8(36.5 to 45) |  |
| Antigua and Barbuda | 34(30 to 37) | 62(55.4 to 67.9) | 85(76 to 94) | 85(76.3 to 94) | 37.2(31.8 to 43.3) |  |
| The Bahamas | 134(121 to 147) | 72.7(66 to 80) | 356(320 to 393) | 93.8(84.2 to 103.2) | 29(23.3 to 35.4) |  |
| Barbados | 183(163 to 201) | 63.4(57.1 to 69.4) | 373(333 to 414) | 86(76.9 to 95.2) | 35.6(29.8 to 41.4) |  |
| Belize | 60(54 to 66) | 52.6(47.2 to 57.3) | 231(207 to 255) | 76.9(68.8 to 84.7) | 46.4(41.6 to 51.8) |  |
| Bermuda | 46(41 to 50) | 71.8(64.8 to 78.7) | 101(90 to 112) | 91.3(81.6 to 100.9) | 27.1(20.8 to 33.2) |  |
| Cuba | 6743(6073 to 7428) | 63.6(57.4 to 70) | 15789(14119 to 17482) | 92.7(83.2 to 102.4) | 45.8(40.5 to 51.8) |  |
| Dominica | 44(40 to 48) | 62.2(56.1 to 68.2) | 75(67 to 83) | 85.8(76.8 to 94.9) | 38(32.2 to 43.8) |  |
| Dominican Republic | 2371(2134 to 2604) | 50.3(45.3 to 55.2) | 7679(6888 to 8489) | 79.7(71.5 to 88.1) | 58.3(53.3 to 63.3) |  |
| Grenada | 40(36 to 43) | 54.7(49.4 to 60) | 109(97 to 120) | 75.6(67.5 to 83.4) | 38.3(32.5 to 44.5) |  |
| Guyana | 276(251 to 302) | 56.7(51.4 to 62) | 515(463 to 569) | 80.8(72.5 to 89.1) | 42.6(36.3 to 49.2) |  |
| Haiti | 1819(1638 to 2002) | 46.5(42 to 51) | 5739(5099 to 6362) | 71.6(64.1 to 78.9) | 54.1(48.6 to 60.1) |  |
| Jamaica | 1061(944 to 1165) | 55.2(49.2 to 60.9) | 2301(2057 to 2529) | 78.3(70 to 86.1) | 41.8(36.6 to 47.3) |  |
| Puerto Rico | 3052(2757 to 3354) | 82.5(74.6 to 90.6) | 6139(5433 to 6825) | 98.2(87.5 to 108.2) | 19(13.2 to 24.7) |  |
| Saint Lucia | 51(46 to 56) | 51.2(45.7 to 56.2) | 158(141 to 175) | 77.4(69 to 85.8) | 51.2(45.9 to 56.4) |  |
| Saint Vincent and the Grenadines | 50(45 to 54) | 61.4(55.9 to 67.2) | 109(98 to 121) | 82.6(74 to 91.4) | 34.6(29.4 to 40.1) |  |
| Suriname | 169(153 to 185) | 58.7(53.1 to 64.1) | 490(438 to 541) | 84.4(75.4 to 92.7) | 43.9(37.9 to 50.1) |  |
| Trinidad and Tobago | 543(488 to 594) | 57.2(51.4 to 62.8) | 1377(1227 to 1523) | 81.5(72.7 to 89.8) | 42.3(37 to 48.3) |  |
| Virgin Islands, U.S. | 55(49 to 60) | 60.2(54.2 to 66.4) | 138(122 to 155) | 87.5(78 to 96.7) | 45.3(39.2 to 52) |  |
| Tropical Latin America | 196313(179628 to 214228) | 181.6(165.5 to 198.2) | 387362(352794 to 422360) | 167(152.3 to 182.1) | -8.1(-11.2 to -4.9) |  |
| Brazil | 193285(176760 to 210958) | 183.2(167 to 199.8) | 378808(345005 to 412953) | 167.4(152.7 to 182.5) | -8.6(-11.8 to -5.4) |  |
| Paraguay | 3029(2728 to 3331) | 118.1(106 to 129.9) | 8554(7701 to 9438) | 152.1(136.2 to 167.9) | 28.8(23.4 to 34.8) |  |
| East Asia | 451326(403299 to 500434) | 43.7(39.2 to 48.3) | 1159508(1034974 to 1293409) | 59(52.9 to 65.5) | 34.9(31.2 to 38.9) |  |
| China | 433605(387451 to 480999) | 44.3(39.7 to 49) | 1106325(986735 to 1234501) | 59.3(53.1 to 65.9) | 33.8(30.1 to 37.9) |  |
| North Korea | 5428(4868 to 6011) | 30.6(27.7 to 33.8) | 14985(13299 to 16751) | 48.5(43.1 to 54) | 58.4(52 to 64.3) |  |
| Taiwan (Province of China) | 4774(4293 to 5273) | 27.1(24.5 to 29.8) | 19518(17471 to 21580) | 55.3(49.8 to 61.2) | 104.2(94.8 to 115.2) |  |
| Southeast Asia | 115520(103937 to 126943) | 35.8(32.4 to 39.2) | 314005(277968 to 350287) | 49.3(43.9 to 54.8) | 37.7(33.6 to 41.6) |  |
| Cambodia | 1951(1752 to 2156) | 32.5(29.3 to 36.1) | 5821(5138 to 6489) | 44.9(40 to 50.1) | 38.2(32.2 to 44.6) |  |
| Indonesia | 51188(46046 to 56297) | 40.6(36.7 to 44.5) | 124044(109899 to 138085) | 52.2(46.6 to 58) | 28.5(24.5 to 32.3) |  |
| Laos | 822(740 to 908) | 30.8(27.7 to 34) | 2377(2095 to 2662) | 45.9(40.5 to 51.5) | 48.9(42.2 to 55.5) |  |
| Malaysia | 4632(4161 to 5107) | 40.1(36.2 to 44.2) | 16033(14215 to 17858) | 57.8(51.2 to 64.4) | 44.1(37.7 to 50.1) |  |
| Maldives | 36(32 to 40) | 28.5(25.4 to 31.5) | 190(166 to 212) | 49.1(43.3 to 54.7) | 72.4(66.5 to 79.2) |  |
| Mauritius | 492(449 to 540) | 52.8(48.1 to 57.7) | 945(846 to 1046) | 58.7(52.7 to 64.8) | 11.2(3.4 to 19.1) |  |
| Myanmar | 7989(7169 to 8794) | 27.5(24.7 to 30.2) | 20817(18321 to 23268) | 42.2(37.5 to 46.9) | 53.6(47.2 to 59.9) |  |
| Philippines | 12905(11618 to 14175) | 31.6(28.6 to 34.6) | 38352(33923 to 42669) | 45.7(40.8 to 50.7) | 44.5(38.1 to 50.8) |  |
| Sri Lanka | 3827(3394 to 4245) | 28.5(25.5 to 31.5) | 10954(9641 to 12262) | 45.2(39.9 to 50.4) | 58.4(52.4 to 65.3) |  |
| Seychelles | 24(22 to 26) | 40.1(36.3 to 44.1) | 61(54 to 68) | 54.3(48.2 to 60.5) | 35.4(28.5 to 43.1) |  |
| Thailand | 13971(12517 to 15421) | 31.4(28.4 to 34.5) | 45891(40490 to 51127) | 48.6(43.2 to 53.9) | 54.6(48.7 to 60.4) |  |
| Timor-Leste | 155(138 to 172) | 33.9(30.5 to 37.5) | 456(403 to 510) | 50.6(44.8 to 56.6) | 49.2(42.9 to 55.8) |  |
| Vietnam | 17374(15662 to 19114) | 37.2(33.4 to 40.8) | 47650(41956 to 53450) | 48.7(43.2 to 54.4) | 31.1(25.7 to 36.4) |  |
| Oceania | 906(803 to 1005) | 22(19.7 to 24.3) | 2706(2374 to 3035) | 31(27.4 to 34.6) | 41(36.6 to 45.3) |  |
| American Samoa | 10(9 to 11) | 32.9(29.6 to 36.1) | 21(19 to 23) | 44(39.3 to 49) | 33.9(28.7 to 39.3) |  |
| Federated States of Micronesia | 17(16 to 19) | 27.6(24.9 to 30.5) | 30(26 to 33) | 36.2(32.2 to 40.4) | 31.3(25.6 to 37.1) |  |
| Fiji | 129(115 to 144) | 24.8(22.2 to 27.5) | 276(243 to 309) | 33.7(29.8 to 37.4) | 35.9(31 to 41) |  |
| Guam | 29(26 to 32) | 29(25.8 to 32) | 68(60 to 76) | 38.3(34 to 42.4) | 32(27.8 to 36.6) |  |
| Kiribati | 14(12 to 15) | 27.5(24.8 to 30.3) | 31(27 to 34) | 35.9(31.9 to 40.1) | 30.8(25.3 to 36.8) |  |
| Marshall Islands | 7(6 to 7) | 27.6(24.8 to 30.6) | 15(13 to 17) | 36.4(32.1 to 40.7) | 31.6(26.2 to 37.7) |  |
| Northern Mariana Islands | 10(8 to 11) | 32.1(28.8 to 35.3) | 22(20 to 25) | 42.3(37.6 to 47.3) | 31.9(26.9 to 36.9) |  |
| Papua New Guinea | 507(448 to 563) | 19.1(17 to 21.2) | 1789(1564 to 2012) | 28.7(25.3 to 32.1) | 50.3(44.6 to 55.6) |  |
| Samoa | 30(27 to 33) | 28.5(25.5 to 31.6) | 58(51 to 65) | 37.7(33.4 to 42) | 32.5(27.9 to 37.3) |  |
| Solomon Islands | 44(39 to 48) | 21.4(19.1 to 23.7) | 134(118 to 150) | 31(27.3 to 34.6) | 44.4(39.3 to 50.3) |  |
| Tonga | 21(19 to 23) | 32.6(29.4 to 35.8) | 37(33 to 41) | 42.5(37.9 to 47.4) | 30.5(24.7 to 35.7) |  |
| Vanuatu | 28(25 to 31) | 30.4(27.2 to 33.7) | 77(67 to 86) | 37.6(33.1 to 42.1) | 23.5(18.8 to 29) |  |
| North Africa and Middle East | 88285(78050 to 98750) | 38.5(34 to 42.7) | 249481(219520 to 279783) | 49(43.2 to 54.4) | 27.2(24.3 to 30.1) |  |
| Afghanistan | 2683(2365 to 2998) | 35.2(31 to 39.4) | 7905(6925 to 8946) | 43.8(38.6 to 48.9) | 24.3(20.4 to 29.1) |  |
| Algeria | 6705(5934 to 7462) | 40.7(36 to 45.4) | 18604(16264 to 20886) | 49.4(43.4 to 55.3) | 21.4(17.1 to 25.6) |  |
| Bahrain | 160(142 to 182) | 47.5(42.2 to 52.8) | 795(688 to 905) | 53.6(47.1 to 59.6) | 12.8(8.3 to 17.3) |  |
| Egypt | 14663(12940 to 16430) | 37.9(33.5 to 42.2) | 37005(32352 to 41596) | 49.4(43.3 to 55) | 30.3(25.8 to 34.8) |  |
| Iran | 12385(10947 to 13773) | 33.7(29.7 to 37.4) | 37729(33117 to 42167) | 47(41.5 to 52.4) | 39.5(36.8 to 42.5) |  |
| Iraq | 4015(3558 to 4497) | 37.1(32.7 to 41.3) | 14108(12401 to 15980) | 45.2(39.5 to 50.7) | 21.8(17.6 to 26.1) |  |
| Jordan | 1355(1208 to 1523) | 61.8(54.9 to 68.9) | 6746(5938 to 7559) | 82.3(72.5 to 92) | 33.1(27.9 to 38.4) |  |
| Kuwait | 559(494 to 635) | 45(40.1 to 49.7) | 2068(1810 to 2341) | 51.9(45.8 to 58) | 15.4(11.3 to 19.6) |  |
| Lebanon | 1196(1061 to 1332) | 42.5(37.7 to 47.4) | 3746(3287 to 4196) | 53.4(47 to 59.6) | 25.6(21.3 to 30.5) |  |
| Libya | 1127(1001 to 1258) | 43.1(38.1 to 48.1) | 3102(2709 to 3500) | 52.1(45.8 to 58.2) | 20.8(16.7 to 25.1) |  |
| Morocco | 7148(6325 to 7978) | 39.3(34.6 to 43.8) | 17018(14898 to 19102) | 49.2(43.1 to 55) | 25.2(21 to 29.5) |  |
| Palestine | 517(460 to 577) | 44.4(39.3 to 49.5) | 1663(1463 to 1864) | 50.3(44.3 to 56.1) | 13.3(10.1 to 16.9) |  |
| Oman | 471(417 to 530) | 39.2(34.5 to 43.6) | 1790(1554 to 2047) | 49.7(43.6 to 55.2) | 26.7(23.1 to 31.2) |  |
| Qatar | 145(127 to 166) | 47.5(42.3 to 52.9) | 1222(1059 to 1404) | 53.2(46.8 to 59.5) | 12.2(8 to 16.9) |  |
| Saudi Arabia | 4362(3834 to 4918) | 44.8(39.5 to 50.1) | 15170(13235 to 17206) | 53.6(47.1 to 59.9) | 19.6(15.5 to 23.9) |  |
| Sudan | 4682(4121 to 5233) | 36.3(31.9 to 40.4) | 11870(10411 to 13290) | 46.3(40.7 to 51.7) | 27.6(23.8 to 31.3) |  |
| Syria | 3062(2719 to 3406) | 40.8(36 to 45.5) | 7583(6617 to 8500) | 49.8(43.7 to 55.4) | 21.9(18 to 26.3) |  |
| Tunisia | 2614(2313 to 2925) | 42(37 to 47) | 6452(5657 to 7211) | 51.6(45.4 to 57.4) | 22.8(18.4 to 27) |  |
| Turkey | 17050(15130 to 19071) | 39(34.7 to 43.5) | 40996(36368 to 45536) | 46.4(41.2 to 51.3) | 18.8(14.5 to 23.4) |  |
| United Arab Emirates | 517(454 to 590) | 40.2(35.7 to 44.8) | 4809(4135 to 5617) | 49.7(43.7 to 55.8) | 23.5(19 to 27.6) |  |
| Yemen | 2812(2487 to 3148) | 36.7(32.3 to 40.9) | 8867(7786 to 10000) | 47(41.2 to 52.5) | 28(24.1 to 32.3) |  |
| South Asia | 286138(254650 to 319741) | 33.2(29.6 to 36.8) | 824315(728591 to 926570) | 50.4(44.7 to 56.2) | 51.6(48.7 to 54.4) |  |
| Bangladesh | 24041(21441 to 26914) | 32(28.5 to 35.5) | 61567(54099 to 69147) | 42.6(37.6 to 47.7) | 33.2(28.7 to 38.4) |  |
| Bhutan | 148(133 to 166) | 38.1(34.1 to 42.1) | 385(338 to 435) | 45.4(40.1 to 50.7) | 19.1(14.2 to 23.5) |  |
| India | 228408(202874 to 255611) | 32.8(29.2 to 36.4) | 680196(602335 to 763419) | 52.3(46.4 to 58.4) | 59.3(56.4 to 62.1) |  |
| Nepal | 4900(4386 to 5489) | 34.9(31 to 38.8) | 10839(9540 to 12185) | 41.9(36.9 to 47.2) | 20.2(15.8 to 25) |  |
| Pakistan | 28640(25729 to 31827) | 37.6(33.7 to 41.6) | 71328(63265 to 80129) | 43.8(38.9 to 48.8) | 16.7(12.5 to 21.3) |  |
| Southern Sub-Saharan Africa | 6886(6112 to 7681) | 17.8(15.9 to 19.7) | 12884(11300 to 14458) | 18.8(16.5 to 21) | 5.4(2.6 to 8.1) |  |
| Botswana | 158(140 to 176) | 18.5(16.4 to 20.5) | 373(325 to 421) | 19.4(17 to 21.7) | 4.9(1 to 8.6) |  |
| Lesotho | 228(201 to 254) | 17.7(15.6 to 19.7) | 282(247 to 316) | 17.9(15.6 to 19.9) | 1(-2.8 to 4.8) |  |
| Namibia | 173(154 to 193) | 17.8(15.8 to 19.7) | 363(319 to 407) | 19.6(17.1 to 21.9) | 10.1(6 to 14.2) |  |
| South Africa | 5159(4578 to 5752) | 18(16.1 to 19.9) | 9873(8657 to 11075) | 19(16.7 to 21.2) | 5.1(2.3 to 8) |  |
| Swaziland | 83(73 to 92) | 16.8(15 to 18.7) | 153(134 to 172) | 18.4(16.1 to 20.6) | 9.2(4.7 to 13.5) |  |
| Zimbabwe | 1085(959 to 1219) | 16.8(14.9 to 18.6) | 1841(1611 to 2074) | 17.7(15.5 to 19.8) | 5.4(1.5 to 9.6) |  |
| Western Sub-Saharan Africa | 32266(28600 to 35836) | 25.9(22.9 to 28.7) | 79851(69618 to 90046) | 29(25.3 to 32.5) | 11.8(8.1 to 15.3) |  |
| Benin | 707(625 to 786) | 24.5(21.6 to 27.3) | 2088(1826 to 2356) | 28.9(25.2 to 32.3) | 17.6(13.2 to 21.9) |  |
| Burkina Faso | 1360(1209 to 1508) | 22.8(20.2 to 25.2) | 3663(3192 to 4131) | 27.6(24 to 31) | 20.7(14.8 to 27.1) |  |
| Cameroon | 1765(1573 to 1961) | 27.1(24.1 to 30) | 5135(4468 to 5810) | 28.6(24.9 to 32.3) | 5.7(0 to 11.4) |  |
| Cape Verde | 61(55 to 67) | 26.3(23.4 to 29) | 159(140 to 178) | 32.1(28.2 to 35.9) | 22.3(17.4 to 27.4) |  |
| Chad | 931(829 to 1034) | 24.8(22 to 27.5) | 2319(2036 to 2618) | 27.2(23.9 to 30.5) | 9.8(5.3 to 14.6) |  |
| Cote d'Ivoire | 1766(1560 to 1966) | 23.9(21.1 to 26.4) | 4738(4140 to 5379) | 27.6(24.2 to 30.9) | 15.9(10.9 to 21.1) |  |
| The Gambia | 164(145 to 182) | 28.3(25.2 to 31.3) | 403(352 to 453) | 29.3(25.6 to 32.9) | 3.4(-0.8 to 8.2) |  |
| Ghana | 2438(2152 to 2712) | 25.4(22.6 to 28) | 6222(5401 to 7056) | 28(24.3 to 31.4) | 10.1(4.9 to 15.6) |  |
| Guinea | 992(875 to 1109) | 23.5(20.7 to 26.3) | 2011(1753 to 2279) | 26.5(23.2 to 29.9) | 12.7(8.6 to 17.1) |  |
| Guinea-Bissau | 149(132 to 167) | 24.4(21.7 to 27.2) | 305(266 to 346) | 26(22.7 to 29.2) | 6.4(1.9 to 10.9) |  |
| Liberia | 319(279 to 355) | 22.9(20 to 25.4) | 877(760 to 1001) | 27.3(23.8 to 30.9) | 19.6(15.3 to 24.2) |  |
| Mali | 1309(1152 to 1470) | 23.2(20.4 to 26) | 3536(3079 to 3997) | 29.4(25.5 to 33.2) | 26.6(22.2 to 31.1) |  |
| Mauritania | 370(330 to 410) | 27.2(24.2 to 30.1) | 799(701 to 899) | 29.8(26.1 to 33.5) | 9.7(4.9 to 14.5) |  |
| Niger | 1192(1059 to 1330) | 25.5(22.7 to 28.2) | 3379(2963 to 3800) | 29.1(25.5 to 32.8) | 14(9.1 to 19.8) |  |
| Nigeria | 16395(14495 to 18277) | 27.1(23.7 to 30.1) | 38216(33115 to 43245) | 29.7(25.9 to 33.5) | 9.7(5.2 to 14.3) |  |
| Sao Tome and Principe | 21(19 to 23) | 26.9(23.9 to 29.7) | 42(37 to 48) | 28.3(24.8 to 31.8) | 5.5(0.9 to 10.3) |  |
| Senegal | 1245(1112 to 1381) | 26.9(24 to 29.7) | 2954(2576 to 3327) | 29.6(25.8 to 33.2) | 10.2(5 to 15.6) |  |
| Sierra Leone | 579(508 to 648) | 22.4(19.6 to 24.9) | 1475(1283 to 1667) | 28.5(24.8 to 32.1) | 27.1(22.1 to 32.6) |  |
| Togo | 503(446 to 562) | 23.6(21 to 26.2) | 1531(1328 to 1731) | 28.5(24.8 to 32.1) | 20.9(15.6 to 26.3) |  |
| Eastern Sub-Saharan Africa | 19230(16996 to 21507) | 16.1(14.3 to 17.9) | 46949(41160 to 52900) | 18.7(16.4 to 20.9) | 15.9(13.4 to 18.6) |  |
| Burundi | 520(459 to 582) | 15(13.3 to 16.7) | 1204(1046 to 1365) | 17.6(15.3 to 19.7) | 17.1(12.8 to 21.5) |  |
| Comoros | 54(48 to 61) | 18.4(16.3 to 20.5) | 115(101 to 129) | 19.9(17.5 to 22.2) | 7.9(4 to 11.6) |  |
| Djibouti | 52(46 to 60) | 17.6(15.6 to 19.8) | 171(148 to 194) | 19.5(17.1 to 21.8) | 10.4(6.5 to 14.4) |  |
| Eritrea | 280(247 to 316) | 16.1(14.3 to 17.9) | 711(621 to 810) | 18.2(16.1 to 20.4) | 13.6(9.9 to 17.2) |  |
| Ethiopia | 4818(4245 to 5428) | 14.6(12.9 to 16.3) | 11871(10384 to 13430) | 17.9(15.7 to 20) | 22.2(19.3 to 25.2) |  |
| Kenya | 2610(2305 to 2909) | 19.6(17.4 to 21.7) | 6952(6091 to 7802) | 21.5(18.9 to 23.9) | 9.5(7.1 to 11.9) |  |
| Madagascar | 1224(1076 to 1382) | 15.8(13.9 to 17.6) | 3150(2748 to 3579) | 18.5(16.2 to 20.7) | 17.3(13.8 to 20.7) |  |
| Malawi | 971(859 to 1087) | 15.8(13.9 to 17.5) | 2063(1809 to 2319) | 18.6(16.3 to 20.8) | 18.2(14.1 to 22) |  |
| Mozambique | 1546(1372 to 1729) | 16.4(14.6 to 18.3) | 3459(3025 to 3909) | 19.1(16.7 to 21.4) | 15.9(11.4 to 20.7) |  |
| Rwanda | 766(678 to 856) | 17.2(15.3 to 19.1) | 1635(1437 to 1837) | 19.2(16.9 to 21.5) | 12(7.6 to 16.8) |  |
| Somalia | 721(632 to 815) | 15.5(13.7 to 17.4) | 1861(1630 to 2106) | 17.5(15.4 to 19.5) | 12.9(9.2 to 16.8) |  |
| South Sudan | 637(564 to 716) | 17(15 to 18.9) | 1142(995 to 1296) | 18.3(16.1 to 20.5) | 8(4.1 to 11.7) |  |
| Tanzania | 2600(2299 to 2909) | 16(14.2 to 17.8) | 6569(5741 to 7411) | 18.6(16.3 to 20.9) | 16.5(12.7 to 20.9) |  |
| Uganda | 1628(1436 to 1828) | 15.8(14 to 17.6) | 4100(3595 to 4636) | 18(15.8 to 20.2) | 13.9(10.2 to 17.7) |  |
| Zambia | 793(703 to 891) | 17(15.1 to 19) | 1919(1674 to 2167) | 17.6(15.4 to 19.7) | 3.6(-0.3 to 7.4) |  |
| Central Sub-Saharan Africa | 6065(5352 to 6780) | 16.9(14.9 to 18.8) | 15214(13313 to 17228) | 18.6(16.3 to 20.8) | 10.3(7.2 to 13.2) |  |
| Angola | 1121(986 to 1262) | 16.8(14.8 to 18.7) | 3326(2902 to 3776) | 18.7(16.3 to 20.9) | 11(7.6 to 14.4) |  |
| Central African Republic | 301(267 to 338) | 16.4(14.6 to 18.3) | 572(495 to 644) | 16.9(14.9 to 18.9) | 3.2(-0.8 to 6.9) |  |
| Congo | 265(234 to 298) | 16.4(14.5 to 18.3) | 699(611 to 795) | 18.4(16.2 to 20.6) | 12.1(8.2 to 15.9) |  |
| Democratic Republic of the Congo | 4206(3715 to 4702) | 17(15 to 18.8) | 10186(8918 to 11446) | 18.7(16.4 to 20.9) | 10.3(6.7 to 13.8) |  |
| Equatorial Guinea | 50(44 to 56) | 17.6(15.6 to 19.6) | 169(148 to 193) | 20.4(17.9 to 22.9) | 15.5(11.4 to 19.4) |  |
| Gabon | 122(108 to 135) | 17(15 to 19) | 262(230 to 295) | 19(16.6 to 21.3) | 11.4(7.6 to 15.1) |  |
|  |  |  |  |  |  |  |

| Table S5: Incident cases of pancreatitis in 1990 and 2017 for both sexes and percentage change of age-standardised rates (ASR) by location | | | | | |  |
| --- | --- | --- | --- | --- | --- | --- |
|  | **1990** | | **2017** | | **Percentage change in ASR from 1990 and 2017** |  |
|  | **Counts (95% UI)** | **Rate (95% UI)** | **Counts (95% UI)** | **Rate (95% UI)** |  |  |
| Global | 1010993(923565 to 1101077) | 21.9(20.1 to 23.8) | 1644222(1525569 to 1769526) | 20.6(19.2 to 22.1) | -6(-7.6 to -4.2) |  |
| High-income North America | 204856(185375 to 224158) | 63.9(57.8 to 70.1) | 284791(266555 to 303177) | 60.2(56.5 to 63.9) | -5.8(-9.7 to -1.2) |  |
| Canada | 18650(16877 to 20404) | 59.9(54.4 to 65.7) | 29261(26650 to 32064) | 59(53.5 to 64.4) | -1.5(-3.7 to 0.7) |  |
| Greenland | 29(27 to 33) | 58.3(52.8 to 63.8) | 37(33 to 41) | 56.6(51.6 to 61.4) | -3(-5.4 to -0.4) |  |
| United States | 186172(168365 to 203769) | 64.4(58.2 to 70.5) | 255488(239652 to 271649) | 60.3(56.8 to 64) | -6.2(-10.6 to -1.2) |  |
| Australasia | 6020(5482 to 6574) | 26.7(24.4 to 29.1) | 9540(8740 to 10416) | 25.4(23.2 to 27.6) | -4.8(-6.9 to -2.7) |  |
| Australia | 4936(4488 to 5398) | 26.2(23.9 to 28.6) | 7687(6992 to 8429) | 24.2(22.1 to 26.5) | -7.7(-10 to -5.3) |  |
| New Zealand | 1085(990 to 1181) | 29(26.4 to 31.4) | 1852(1723 to 1988) | 31.8(29.7 to 34) | 9.7(5.9 to 13.9) |  |
| High-income Asia Pacific | 55243(50570 to 60077) | 29.1(26.8 to 31.5) | 72075(66885 to 77347) | 29.7(27.7 to 31.8) | 2.1(-0.1 to 4.6) |  |
| Brunei | 47(43 to 51) | 21.4(19.7 to 23.2) | 94(87 to 103) | 21.7(20 to 23.5) | 1(-1.5 to 3.4) |  |
| Japan | 44321(40495 to 48196) | 30.8(28.2 to 33.3) | 55508(51710 to 59431) | 33.5(31.2 to 35.8) | 8.8(5.8 to 12.3) |  |
| Singapore | 473(437 to 513) | 15.5(14.3 to 16.7) | 1278(1193 to 1370) | 19.5(18.2 to 20.7) | 25.7(18.3 to 34.3) |  |
| South Korea | 10402(9548 to 11333) | 24.9(23 to 26.9) | 15195(13920 to 16573) | 23.2(21.4 to 25) | -7.1(-9.2 to -5) |  |
| Western Europe | 105992(97018 to 115053) | 22.3(20.5 to 24.3) | 140633(130519 to 151415) | 23.3(21.6 to 25.1) | 4.2(2.3 to 6.7) |  |
| Andorra | 13(12 to 15) | 21.4(19.6 to 23.4) | 23(21 to 25) | 20.8(19.1 to 22.8) | -2.7(-5.3 to -0.3) |  |
| Austria | 2914(2728 to 3094) | 30.8(28.9 to 32.8) | 3565(3330 to 3801) | 29.3(27.4 to 31.3) | -4.8(-8.1 to -1.3) |  |
| Belgium | 3003(2768 to 3257) | 24.6(22.7 to 26.6) | 3656(3386 to 3937) | 25.8(23.9 to 27.8) | 4.8(1.3 to 8.8) |  |
| Cyprus | 159(144 to 176) | 19.4(17.6 to 21.4) | 296(270 to 323) | 18(16.5 to 19.6) | -7.2(-10 to -4.8) |  |
| Denmark | 1004(926 to 1090) | 15.6(14.4 to 16.9) | 1251(1159 to 1352) | 16.8(15.6 to 18.1) | 7.4(4.5 to 10.4) |  |
| Finland | 1587(1460 to 1720) | 26.3(24.2 to 28.4) | 2161(2002 to 2318) | 29.5(27.5 to 31.6) | 12.2(8 to 16.6) |  |
| France | 14470(13220 to 15723) | 20.8(19 to 22.7) | 17280(15813 to 18811) | 19.3(17.7 to 21.1) | -7.1(-9.5 to -4.7) |  |
| Germany | 26697(24349 to 29052) | 26(23.9 to 28.3) | 33162(30523 to 35955) | 27.6(25.4 to 29.9) | 6(2.5 to 10.5) |  |
| Greece | 2607(2374 to 2856) | 20.6(18.8 to 22.5) | 3194(2934 to 3481) | 20.8(19.1 to 22.7) | 1(-1.7 to 3.7) |  |
| Iceland | 63(58 to 69) | 23.4(21.2 to 25.6) | 98(90 to 106) | 23.4(21.5 to 25.4) | 0.1(-2.9 to 3.3) |  |
| Ireland | 971(878 to 1063) | 25.9(23.4 to 28.5) | 1563(1420 to 1708) | 26.2(23.8 to 28.5) | 1(-1.7 to 3.6) |  |
| Israel | 931(850 to 1017) | 19.5(17.8 to 21.3) | 1895(1741 to 2069) | 19.7(18.1 to 21.6) | 1.4(-1.1 to 3.9) |  |
| Italy | 16111(14646 to 17621) | 22.5(20.5 to 24.5) | 21170(19523 to 22687) | 21.6(20.2 to 23) | -3.8(-7.5 to 1) |  |
| Luxembourg | 107(98 to 117) | 22.4(20.5 to 24.3) | 180(166 to 195) | 23.3(21.5 to 25.1) | 4.2(0.4 to 9.2) |  |
| Malta | 67(60 to 74) | 16.2(14.7 to 17.8) | 95(88 to 104) | 15.2(14 to 16.6) | -5.8(-8.9 to -3) |  |
| Netherlands | 1748(1585 to 1920) | 9.7(8.8 to 10.7) | 2181(1977 to 2395) | 9.7(8.8 to 10.6) | -0.2(-3.5 to 3) |  |
| Norway | 1721(1582 to 1869) | 33.4(30.7 to 36.3) | 2676(2480 to 2885) | 39.4(36.6 to 42.4) | 17.8(14.7 to 20.9) |  |
| Portugal | 2356(2152 to 2577) | 20(18.3 to 21.9) | 3060(2817 to 3320) | 19.6(18 to 21.1) | -2.2(-5.1 to 0.9) |  |
| Spain | 6897(6324 to 7501) | 14.8(13.6 to 16.1) | 9499(8720 to 10330) | 14.2(13 to 15.4) | -4.2(-6.8 to -1.2) |  |
| Sweden | 2691(2460 to 2948) | 24.3(22.1 to 26.6) | 3532(3227 to 3850) | 25.8(23.6 to 28.2) | 6.5(4.4 to 8.6) |  |
| Switzerland | 1737(1578 to 1910) | 19.7(17.8 to 21.7) | 2492(2310 to 2675) | 20.4(19 to 22) | 3.9(-0.7 to 10) |  |
| United Kingdom | 18036(16553 to 19642) | 25.5(23.4 to 27.7) | 27458(25537 to 29481) | 31.8(29.6 to 34.1) | 24.9(21.9 to 28.2) |  |
| Southern Latin America | 10540(9617 to 11484) | 22.1(20.1 to 24) | 15022(13732 to 16360) | 20.1(18.4 to 21.9) | -8.8(-10.5 to -6.7) |  |
| Argentina | 7279(6650 to 7943) | 22.4(20.5 to 24.4) | 9659(8818 to 10515) | 19.7(18 to 21.4) | -12.2(-14.1 to -10.2) |  |
| Chile | 2571(2339 to 2815) | 21.7(19.8 to 23.7) | 4555(4142 to 5022) | 21.4(19.5 to 23.6) | -1.4(-5.4 to 4.4) |  |
| Uruguay | 689(627 to 755) | 19.8(17.9 to 21.6) | 808(739 to 886) | 18.9(17.2 to 20.6) | -4.7(-6.9 to -2.4) |  |
| Eastern Europe | 105127(96174 to 113936) | 41(37.7 to 44.3) | 135287(124571 to 146178) | 50(46.1 to 53.7) | 22(20.5 to 23.5) |  |
| Belarus | 4652(4245 to 5061) | 39.5(36.2 to 42.9) | 5628(5160 to 6106) | 45.5(41.9 to 49.3) | 15.3(12.5 to 18.2) |  |
| Estonia | 722(660 to 783) | 40(36.7 to 43.3) | 742(682 to 804) | 41.9(38.4 to 45.4) | 5(2.4 to 7.8) |  |
| Latvia | 1223(1118 to 1331) | 38.8(35.6 to 42.1) | 1230(1133 to 1331) | 46.9(43.1 to 50.6) | 20.7(16.5 to 25.9) |  |
| Lithuania | 1719(1569 to 1870) | 41.1(37.6 to 44.6) | 2081(1919 to 2248) | 54.2(50.2 to 58.3) | 31.9(27.7 to 36.7) |  |
| Moldova | 2335(2151 to 2515) | 50.8(47 to 54.6) | 2219(2031 to 2406) | 47.1(43.3 to 50.9) | -7.3(-9.3 to -5.3) |  |
| Russian Federation | 70194(64281 to 76040) | 41.4(38 to 44.7) | 95451(87967 to 103082) | 51.2(47.4 to 55) | 23.7(22.3 to 25.2) |  |
| Ukraine | 24283(22171 to 26453) | 39.5(36.1 to 42.8) | 27936(25645 to 30289) | 47.4(43.6 to 51.1) | 20(17.2 to 22.8) |  |
| Central Europe | 58517(53838 to 63292) | 41.9(38.6 to 45) | 68446(64051 to 73101) | 42.8(40 to 45.6) | 2.1(-0.6 to 5.5) |  |
| Albania | 876(802 to 953) | 32.7(29.9 to 35.6) | 1155(1058 to 1260) | 33.9(31.2 to 36.8) | 3.6(1 to 6.3) |  |
| Bosnia and Herzegovina | 1657(1507 to 1804) | 36.3(33.2 to 39.4) | 1765(1613 to 1920) | 38(35 to 41.2) | 4.7(2 to 7.5) |  |
| Bulgaria | 3795(3468 to 4136) | 34.6(31.7 to 37.5) | 3883(3564 to 4217) | 37.2(34.3 to 40.2) | 7.6(5.1 to 10.3) |  |
| Croatia | 2396(2194 to 2606) | 40.7(37.4 to 44.1) | 2456(2294 to 2615) | 38.7(36.2 to 41.1) | -5(-9.2 to -0.3) |  |
| Czech Republic | 5411(4975 to 5844) | 44.2(40.7 to 47.6) | 6586(6168 to 7009) | 43.7(41 to 46.5) | -1.1(-5.4 to 3.8) |  |
| Hungary | 5626(5184 to 6089) | 43.9(40.6 to 47.3) | 5293(4861 to 5750) | 37.8(34.8 to 40.8) | -13.9(-16.3 to -11.5) |  |
| Macedonia | 706(644 to 770) | 35(32 to 38) | 1020(932 to 1109) | 36(33.1 to 39.1) | 3(0.6 to 5.5) |  |
| Montenegro | 230(210 to 250) | 35.6(32.7 to 38.6) | 287(263 to 312) | 35.6(32.8 to 38.5) | 0(-2.6 to 2.5) |  |
| Poland | 19989(18388 to 21657) | 46.2(42.6 to 49.9) | 25400(23814 to 27094) | 47.8(44.9 to 51) | 3.4(-0.6 to 8) |  |
| Romania | 10469(9587 to 11355) | 40.4(37.3 to 43.6) | 11933(11151 to 12773) | 43.5(40.7 to 46.5) | 7.7(2.6 to 13.7) |  |
| Serbia | 3446(3161 to 3731) | 32(29.4 to 34.5) | 3743(3470 to 4024) | 31(28.8 to 33.2) | -3.1(-6.7 to 0.3) |  |
| Slovakia | 2943(2715 to 3172) | 51.3(47.3 to 55.1) | 3826(3580 to 4091) | 52.9(49.3 to 56.2) | 3.2(-0.2 to 7.5) |  |
| Slovenia | 975(893 to 1052) | 42(38.5 to 45.4) | 1100(1029 to 1176) | 35.8(33.5 to 38.2) | -14.8(-18 to -11.5) |  |
| Central Asia | 7109(6643 to 7630) | 12.5(11.7 to 13.4) | 11424(10552 to 12355) | 12.9(12 to 13.9) | 3.4(1.1 to 5.7) |  |
| Armenia | 306(280 to 332) | 9.7(9 to 10.5) | 411(378 to 447) | 11.3(10.4 to 12.3) | 16.5(13 to 20.1) |  |
| Azerbaijan | 573(527 to 622) | 9.3(8.6 to 10.1) | 1171(1067 to 1284) | 10.9(10 to 11.8) | 17.2(13.9 to 21) |  |
| Georgia | 531(485 to 576) | 9(8.3 to 9.8) | 550(510 to 595) | 11.8(11 to 12.7) | 30.6(26.8 to 34.7) |  |
| Kazakhstan | 2946(2772 to 3154) | 19.4(18.3 to 20.6) | 3308(3081 to 3550) | 17.8(16.7 to 19) | -8(-11.3 to -4.8) |  |
| Kyrgyzstan | 383(355 to 412) | 10.7(9.9 to 11.5) | 669(619 to 722) | 11.8(10.9 to 12.7) | 10.2(7.4 to 13) |  |
| Mongolia | 226(211 to 242) | 15.7(14.6 to 16.7) | 483(442 to 526) | 15.5(14.4 to 16.7) | -0.9(-3.6 to 1.8) |  |
| Tajikistan | 372(343 to 403) | 9.9(9.2 to 10.7) | 873(795 to 953) | 11.5(10.6 to 12.5) | 16.2(13 to 19.7) |  |
| Turkmenistan | 263(243 to 284) | 9.9(9.1 to 10.6) | 562(518 to 610) | 11.8(11 to 12.8) | 20.2(17.2 to 23.3) |  |
| Uzbekistan | 1510(1395 to 1631) | 9.9(9.1 to 10.6) | 3398(3112 to 3704) | 11.5(10.6 to 12.4) | 16.2(13.3 to 19.2) |  |
| Central Latin America | 28203(25818 to 30988) | 23.5(21.6 to 25.6) | 60554(55680 to 65707) | 24.4(22.5 to 26.3) | 3.5(2 to 5.1) |  |
| Colombia | 5563(5075 to 6124) | 22.3(20.4 to 24.2) | 11419(10511 to 12394) | 21.6(19.9 to 23.4) | -3(-5.6 to -0.4) |  |
| Costa Rica | 612(564 to 667) | 26.1(24 to 28.3) | 1261(1163 to 1375) | 25.3(23.4 to 27.6) | -2.8(-5.3 to -0.4) |  |
| El Salvador | 903(831 to 985) | 23.6(21.7 to 25.6) | 1319(1218 to 1436) | 22.4(20.6 to 24.3) | -5.3(-7.6 to -2.9) |  |
| Guatemala | 1379(1275 to 1497) | 26(24 to 28.1) | 3279(3023 to 3573) | 23.8(22 to 25.8) | -8.3(-10.4 to -6.3) |  |
| Honduras | 735(679 to 804) | 24.2(22.3 to 26.3) | 1864(1710 to 2028) | 24.8(22.9 to 26.9) | 2.5(0.2 to 5.1) |  |
| Mexico | 15018(13740 to 16508) | 24.3(22.2 to 26.4) | 32803(30148 to 35592) | 26.5(24.5 to 28.7) | 9.4(7.4 to 11.7) |  |
| Nicaragua | 542(498 to 596) | 22.2(20.4 to 24.3) | 1191(1093 to 1306) | 21.5(19.8 to 23.4) | -3.3(-5.5 to -1) |  |
| Panama | 403(369 to 443) | 21(19.2 to 22.9) | 837(771 to 912) | 21(19.3 to 22.9) | -0.1(-2.5 to 2.5) |  |
| Venezuela | 3047(2776 to 3360) | 21.6(19.8 to 23.5) | 6581(6013 to 7169) | 21.5(19.8 to 23.3) | -0.5(-2.9 to 2) |  |
| Andean Latin America | 5722(5298 to 6205) | 20.7(19.2 to 22.3) | 11537(10685 to 12469) | 20(18.5 to 21.6) | -3.7(-5.9 to -1) |  |
| Bolivia | 928(853 to 1011) | 21.1(19.5 to 22.8) | 2020(1871 to 2189) | 20.4(19 to 22) | -3.3(-5.5 to -0.9) |  |
| Ecuador | 1652(1548 to 1768) | 23.1(21.7 to 24.6) | 3572(3288 to 3892) | 22.7(20.9 to 24.7) | -1.6(-6.8 to 5.5) |  |
| Peru | 3142(2886 to 3419) | 19.6(18 to 21.2) | 5945(5505 to 6423) | 18.5(17.1 to 20) | -5.6(-7.9 to -3.3) |  |
| Caribbean | 4719(4347 to 5131) | 15.8(14.6 to 17.1) | 8371(7750 to 9029) | 16.9(15.6 to 18.2) | 7.1(5.2 to 9.3) |  |
| Antigua and Barbuda | 8(8 to 9) | 15.2(14 to 16.5) | 16(15 to 17) | 15.9(14.7 to 17.2) | 4.7(2 to 7.5) |  |
| The Bahamas | 37(34 to 41) | 18.1(16.7 to 19.5) | 72(67 to 78) | 18.4(17.1 to 19.8) | 1.8(-0.7 to 4.4) |  |
| Barbados | 44(41 to 48) | 15.9(14.7 to 17.3) | 66(61 to 72) | 16.6(15.3 to 17.9) | 4(1.3 to 7) |  |
| Belize | 18(16 to 20) | 14.3(13.1 to 15.5) | 52(48 to 57) | 15.9(14.7 to 17.2) | 11.3(8.6 to 14.1) |  |
| Bermuda | 12(11 to 13) | 17.8(16.3 to 19.2) | 17(16 to 18) | 17.3(16 to 18.7) | -2.7(-5.3 to 0) |  |
| Cuba | 1682(1542 to 1834) | 15.5(14.2 to 16.9) | 2726(2510 to 2952) | 17.5(16.1 to 18.9) | 12.6(9.8 to 15.7) |  |
| Dominica | 11(10 to 12) | 16.4(15 to 17.7) | 14(13 to 15) | 17.3(16 to 18.7) | 5.5(2.9 to 8.4) |  |
| Dominican Republic | 734(673 to 805) | 14.2(13 to 15.4) | 1565(1447 to 1697) | 15.7(14.5 to 16.9) | 10.6(7.7 to 13.4) |  |
| Grenada | 11(10 to 12) | 15(13.8 to 16.3) | 20(18 to 22) | 14.9(13.8 to 16.1) | -0.6(-3.3 to 2.3) |  |
| Guyana | 90(83 to 99) | 16.3(15.1 to 17.6) | 117(108 to 127) | 17.4(16.1 to 18.7) | 6.4(3.9 to 9.2) |  |
| Haiti | 672(619 to 734) | 15.4(14.3 to 16.7) | 1516(1397 to 1649) | 16.8(15.6 to 18.1) | 9.2(6.6 to 12.2) |  |
| Jamaica | 281(259 to 305) | 14.3(13.2 to 15.5) | 443(409 to 480) | 15.1(13.9 to 16.4) | 5.7(2.9 to 8.5) |  |
| Puerto Rico | 713(660 to 768) | 19.4(17.9 to 20.9) | 1005(930 to 1087) | 18.4(17.1 to 19.8) | -5.1(-7.6 to -2.4) |  |
| Saint Lucia | 14(13 to 15) | 13.4(12.3 to 14.5) | 29(27 to 31) | 14.3(13.3 to 15.5) | 6.9(3.9 to 10) |  |
| Saint Vincent and the Grenadines | 14(13 to 15) | 16.2(15 to 17.5) | 22(20 to 23) | 16.8(15.5 to 18.1) | 3.6(1.1 to 5.9) |  |
| Suriname | 50(46 to 54) | 16(14.8 to 17.3) | 103(95 to 111) | 17.5(16.2 to 18.9) | 9.4(6.7 to 12.2) |  |
| Trinidad and Tobago | 152(140 to 167) | 15(13.8 to 16.3) | 264(243 to 285) | 16(14.8 to 17.2) | 6.5(3.6 to 9.3) |  |
| Virgin Islands, U.S. | 15(14 to 16) | 15.2(14.1 to 16.5) | 24(22 to 26) | 16.5(15.2 to 17.8) | 8.1(5.4 to 11.1) |  |
| Tropical Latin America | 23113(21640 to 24775) | 20(18.7 to 21.4) | 47034(44043 to 50327) | 20.2(18.9 to 21.6) | 1.2(-1.3 to 3.9) |  |
| Brazil | 22647(21192 to 24280) | 20(18.8 to 21.4) | 45892(42970 to 49105) | 20.2(18.9 to 21.6) | 0.9(-1.6 to 3.7) |  |
| Paraguay | 467(434 to 502) | 16.9(15.8 to 18.2) | 1142(1064 to 1233) | 19.4(18 to 21) | 14.6(11.2 to 18.3) |  |
| East Asia | 237106(214801 to 260525) | 21.6(19.7 to 23.6) | 429342(393662 to 468409) | 22.7(20.9 to 24.6) | 5.1(3.1 to 7.1) |  |
| China | 226304(204967 to 248606) | 21.7(19.8 to 23.7) | 409296(375371 to 446636) | 22.8(21 to 24.7) | 4.8(2.8 to 6.8) |  |
| North Korea | 3495(3161 to 3834) | 18.8(17.1 to 20.6) | 6002(5483 to 6587) | 19.9(18.2 to 21.7) | 5.4(2.8 to 8.2) |  |
| Taiwan (Province of China) | 3356(3032 to 3721) | 17.9(16.3 to 19.7) | 7127(6501 to 7762) | 21.8(19.9 to 23.7) | 21.4(17.9 to 25.3) |  |
| Southeast Asia | 46619(42752 to 51128) | 13.6(12.6 to 14.7) | 89193(82195 to 96841) | 13.8(12.7 to 14.9) | 1.3(0.2 to 2.5) |  |
| Cambodia | 893(818 to 980) | 13.8(12.8 to 15) | 1901(1746 to 2067) | 14.1(13 to 15.2) | 1.7(-0.7 to 4.1) |  |
| Indonesia | 20447(18737 to 22325) | 15.2(14.1 to 16.4) | 37303(34290 to 40528) | 15.3(14.2 to 16.5) | 0.6(-0.5 to 1.7) |  |
| Laos | 367(336 to 402) | 13.2(12.2 to 14.3) | 770(708 to 841) | 14(12.9 to 15.1) | 5.5(3 to 8.2) |  |
| Malaysia | 1850(1690 to 2035) | 14.5(13.4 to 15.7) | 4331(3966 to 4728) | 15(13.8 to 16.2) | 3.3(1 to 5.8) |  |
| Maldives | 13(12 to 15) | 9.9(9 to 10.8) | 46(41 to 51) | 10.6(9.7 to 11.5) | 6.9(4.2 to 9.6) |  |
| Mauritius | 144(132 to 156) | 14.9(13.7 to 16.1) | 207(190 to 226) | 13.6(12.5 to 14.7) | -8.8(-11 to -6.4) |  |
| Myanmar | 3599(3298 to 3947) | 12(11 to 13.1) | 6191(5690 to 6726) | 12.4(11.4 to 13.4) | 3(0.7 to 5.5) |  |
| Philippines | 5252(4783 to 5803) | 12.1(11 to 13.1) | 11121(10158 to 12146) | 12.8(11.7 to 13.8) | 5.6(2.9 to 8.2) |  |
| Sri Lanka | 1435(1297 to 1584) | 10.2(9.3 to 11.2) | 2533(2302 to 2777) | 10.7(9.7 to 11.6) | 4.5(2.1 to 7.3) |  |
| Seychelles | 9(8 to 9) | 13.7(12.6 to 14.9) | 15(14 to 17) | 13.8(12.7 to 15) | 0.5(-1.9 to 2.9) |  |
| Thailand | 5599(5113 to 6164) | 12(11 to 13.1) | 11466(10496 to 12530) | 12.7(11.7 to 13.8) | 5.9(3.4 to 8.4) |  |
| Timor-Leste | 66(60 to 73) | 13.3(12.2 to 14.4) | 136(126 to 148) | 14.3(13.3 to 15.5) | 7.7(5.2 to 10.2) |  |
| Vietnam | 6883(6310 to 7533) | 13.9(12.7 to 15.1) | 13056(12091 to 14139) | 13.2(12.2 to 14.2) | -5.1(-7.7 to -2.5) |  |
| Oceania | 488(443 to 539) | 11.2(10.2 to 12.2) | 1064(966 to 1174) | 11.3(10.4 to 12.3) | 1.6(0.1 to 3.3) |  |
| American Samoa | 5(4 to 5) | 13.5(12.4 to 14.7) | 7(6 to 7) | 14.1(12.9 to 15.2) | 4.3(2 to 6.7) |  |
| Federated States of Micronesia | 9(8 to 10) | 13.2(12.1 to 14.4) | 11(10 to 12) | 12.9(11.8 to 14) | -2.5(-4.6 to -0.2) |  |
| Fiji | 63(57 to 70) | 11.3(10.3 to 12.4) | 94(86 to 104) | 11.3(10.3 to 12.4) | -0.2(-2.3 to 2.3) |  |
| Guam | 13(12 to 14) | 11.7(10.7 to 12.8) | 21(19 to 23) | 11.8(10.9 to 12.9) | 0.9(-1.3 to 3.3) |  |
| Kiribati | 7(7 to 8) | 13.5(12.5 to 14.7) | 13(12 to 14) | 14(12.9 to 15.2) | 3.2(0.9 to 5.5) |  |
| Marshall Islands | 4(3 to 4) | 12.9(11.8 to 14) | 6(5 to 6) | 12.9(11.8 to 14) | 0.5(-1.9 to 2.8) |  |
| Northern Mariana Islands | 5(4 to 5) | 12.5(11.4 to 13.7) | 7(6 to 7) | 13(11.8 to 14.1) | 3.5(1.1 to 5.9) |  |
| Papua New Guinea | 290(263 to 320) | 10.6(9.7 to 11.6) | 731(661 to 807) | 11(10 to 12) | 3.4(1.1 to 5.8) |  |
| Samoa | 14(13 to 16) | 12.7(11.6 to 13.8) | 21(19 to 23) | 13(11.9 to 14.1) | 2.5(0.2 to 4.9) |  |
| Solomon Islands | 23(21 to 25) | 11(10 to 12) | 52(47 to 58) | 11.3(10.3 to 12.4) | 3.4(1.2 to 5.9) |  |
| Tonga | 10(9 to 10) | 13.9(12.8 to 15.1) | 13(12 to 14) | 14.2(13 to 15.5) | 2.5(0.2 to 5) |  |
| Vanuatu | 14(13 to 16) | 14.1(13 to 15.4) | 31(29 to 34) | 14.4(13.2 to 15.6) | 2.2(0.1 to 4.3) |  |
| North Africa and Middle East | 22546(20579 to 24681) | 9.4(8.6 to 10.2) | 53000(48513 to 57892) | 10(9.2 to 10.8) | 6.5(5.3 to 7.8) |  |
| Afghanistan | 740(678 to 812) | 9.8(9 to 10.7) | 2030(1852 to 2236) | 10.5(9.6 to 11.3) | 6.9(4.6 to 9.3) |  |
| Algeria | 1656(1510 to 1824) | 9.6(8.7 to 10.4) | 3692(3367 to 4048) | 9.6(8.7 to 10.4) | 0.1(-2.1 to 2.5) |  |
| Bahrain | 43(39 to 48) | 11.3(10.3 to 12.3) | 164(147 to 181) | 10.5(9.6 to 11.3) | -7.1(-9.1 to -4.9) |  |
| Egypt | 3735(3410 to 4099) | 9.3(8.6 to 10.2) | 7782(7106 to 8542) | 9.9(9.1 to 10.8) | 6.3(3.7 to 8.8) |  |
| Iran | 3467(3156 to 3832) | 9(8.2 to 9.8) | 8611(7826 to 9451) | 10.4(9.6 to 11.3) | 16.4(15.1 to 17.9) |  |
| Iraq | 993(904 to 1090) | 8.7(7.9 to 9.6) | 2761(2516 to 3036) | 8.4(7.7 to 9.2) | -3.1(-5.5 to -0.6) |  |
| Jordan | 297(271 to 327) | 12.4(11.4 to 13.4) | 1233(1137 to 1343) | 14(12.9 to 15.1) | 12.9(10.1 to 15.6) |  |
| Kuwait | 135(121 to 152) | 9.8(9 to 10.8) | 425(381 to 474) | 9.9(9.1 to 10.8) | 0.9(-1.3 to 3.2) |  |
| Lebanon | 289(264 to 317) | 9.8(8.9 to 10.6) | 732(667 to 806) | 9.8(9 to 10.8) | 0.8(-1.7 to 3.4) |  |
| Libya | 279(255 to 307) | 9.9(9.1 to 10.8) | 636(580 to 699) | 10.1(9.3 to 11) | 1.9(-0.1 to 4.3) |  |
| Morocco | 1808(1649 to 1985) | 9.6(8.7 to 10.4) | 3453(3159 to 3777) | 9.9(9.1 to 10.8) | 3.8(1.5 to 6.1) |  |
| Palestine | 132(121 to 146) | 10.5(9.6 to 11.5) | 365(334 to 401) | 10.3(9.4 to 11.2) | -2.1(-4.3 to 0.2) |  |
| Oman | 113(102 to 125) | 8.7(7.9 to 9.5) | 353(315 to 396) | 8.7(8 to 9.5) | 0.6(-1.9 to 3.3) |  |
| Qatar | 38(34 to 43) | 10.7(9.8 to 11.6) | 267(236 to 301) | 9.9(9.1 to 10.8) | -7.3(-9.6 to -5) |  |
| Saudi Arabia | 1134(1031 to 1250) | 10.5(9.7 to 11.4) | 3219(2900 to 3578) | 10.2(9.4 to 11.1) | -2.8(-5 to -0.5) |  |
| Sudan | 1207(1105 to 1325) | 9(8.2 to 9.8) | 2661(2439 to 2916) | 9.7(8.8 to 10.5) | 7.2(4.9 to 9.8) |  |
| Syria | 776(709 to 854) | 9.7(8.9 to 10.6) | 1483(1352 to 1624) | 9.5(8.7 to 10.3) | -1.7(-4.1 to 0.7) |  |
| Tunisia | 631(576 to 693) | 9.8(8.9 to 10.7) | 1243(1136 to 1359) | 10(9.2 to 10.9) | 2.6(0.5 to 5.1) |  |
| Turkey | 4171(3799 to 4562) | 9.2(8.4 to 10) | 8751(8051 to 9501) | 10(9.2 to 10.8) | 7.9(5.1 to 11) |  |
| United Arab Emirates | 138(123 to 155) | 9.5(8.6 to 10.3) | 1047(909 to 1191) | 9.6(8.8 to 10.4) | 1.6(-0.8 to 3.9) |  |
| Yemen | 749(682 to 825) | 9.3(8.6 to 10.2) | 2041(1860 to 2240) | 10(9.2 to 10.9) | 7.1(4.9 to 9.4) |  |
| South Asia | 77641(70782 to 85443) | 8.8(8.1 to 9.7) | 180789(165583 to 197571) | 10.9(10 to 11.8) | 23.4(21.8 to 25) |  |
| Bangladesh | 6732(6107 to 7461) | 8.5(7.8 to 9.3) | 13413(12258 to 14659) | 9.1(8.4 to 10) | 7(4.6 to 9.6) |  |
| Bhutan | 40(37 to 45) | 9.8(9 to 10.6) | 86(79 to 96) | 9.8(9 to 10.7) | 0.1(-2.1 to 2.3) |  |
| India | 61627(56187 to 67925) | 8.7(8 to 9.5) | 147193(134867 to 160869) | 11.2(10.3 to 12.2) | 28.7(26.9 to 30.6) |  |
| Nepal | 1320(1205 to 1452) | 9.1(8.4 to 9.9) | 2530(2318 to 2776) | 9.6(8.8 to 10.4) | 4.9(2.8 to 7.4) |  |
| Pakistan | 7922(7252 to 8686) | 9.9(9.1 to 10.8) | 17566(16080 to 19238) | 10.4(9.6 to 11.2) | 4.7(2.4 to 7.2) |  |
| Southern Sub-Saharan Africa | 1198(1087 to 1317) | 3(2.8 to 3.3) | 2054(1859 to 2253) | 3(2.7 to 3.2) | -1.2(-2.9 to 0.5) |  |
| Botswana | 28(25 to 31) | 3.2(2.9 to 3.4) | 58(52 to 64) | 3(2.7 to 3.2) | -6.1(-8.8 to -3.6) |  |
| Lesotho | 43(39 to 47) | 3.3(3 to 3.6) | 52(47 to 58) | 3.3(3 to 3.6) | 1.1(-1.4 to 3.7) |  |
| Namibia | 31(28 to 34) | 3.1(2.9 to 3.4) | 60(54 to 66) | 3.2(2.9 to 3.5) | 1.2(-1.5 to 3.8) |  |
| South Africa | 893(812 to 980) | 3(2.8 to 3.3) | 1526(1382 to 1671) | 2.9(2.7 to 3.2) | -3.4(-5.1 to -1.8) |  |
| Swaziland | 15(14 to 17) | 3.1(2.8 to 3.3) | 26(24 to 29) | 3.1(2.8 to 3.4) | 1.7(-1.1 to 4.5) |  |
| Zimbabwe | 188(169 to 208) | 2.8(2.6 to 3.1) | 331(297 to 366) | 3.2(2.9 to 3.5) | 12.2(8.7 to 15.6) |  |
| Western Sub-Saharan Africa | 5639(5153 to 6157) | 4.5(4.1 to 4.8) | 13537(12306 to 14940) | 4.8(4.4 to 5.2) | 7.5(5 to 10.1) |  |
| Benin | 128(116 to 141) | 4.3(4 to 4.6) | 356(324 to 392) | 4.8(4.3 to 5.2) | 11(8.1 to 14.1) |  |
| Burkina Faso | 257(236 to 279) | 4.3(4 to 4.7) | 658(595 to 729) | 4.9(4.4 to 5.3) | 12(8 to 16.1) |  |
| Cameroon | 325(299 to 353) | 5(4.6 to 5.4) | 914(824 to 1012) | 5(4.6 to 5.5) | 0.5(-3 to 4.1) |  |
| Cape Verde | 9(9 to 10) | 3.8(3.5 to 4.1) | 22(20 to 24) | 4.3(4 to 4.7) | 14.9(11.6 to 18.3) |  |
| Chad | 175(161 to 191) | 4.5(4.2 to 4.9) | 420(383 to 462) | 4.8(4.4 to 5.2) | 5.5(2.7 to 8.3) |  |
| Cote d'Ivoire | 325(295 to 356) | 4.4(4 to 4.7) | 845(764 to 933) | 4.9(4.5 to 5.4) | 12.9(9.9 to 16.4) |  |
| The Gambia | 28(26 to 31) | 4.7(4.4 to 5.1) | 68(61 to 74) | 4.7(4.3 to 5.1) | -0.1(-3 to 2.6) |  |
| Ghana | 433(395 to 471) | 4.5(4.1 to 4.8) | 1040(938 to 1150) | 4.6(4.2 to 5) | 3.4(-0.3 to 7.2) |  |
| Guinea | 180(165 to 196) | 4.2(3.9 to 4.6) | 348(316 to 383) | 4.5(4.1 to 4.9) | 5.9(3.1 to 9.1) |  |
| Guinea-Bissau | 30(27 to 33) | 5(4.6 to 5.4) | 58(52 to 64) | 4.9(4.5 to 5.3) | -1.4(-4.2 to 1.8) |  |
| Liberia | 56(51 to 62) | 4(3.7 to 4.4) | 145(130 to 161) | 4.5(4.1 to 4.9) | 11.7(8.8 to 14.7) |  |
| Mali | 231(210 to 253) | 4.1(3.8 to 4.5) | 596(541 to 655) | 4.8(4.4 to 5.2) | 16.8(13.8 to 19.7) |  |
| Mauritania | 62(57 to 68) | 4.4(4.1 to 4.8) | 123(112 to 136) | 4.5(4.1 to 4.9) | 1.5(-1.4 to 4.4) |  |
| Niger | 216(197 to 236) | 4.6(4.2 to 4.9) | 594(538 to 656) | 5(4.5 to 5.4) | 8.7(5.5 to 12.4) |  |
| Nigeria | 2767(2525 to 3027) | 4.5(4.1 to 4.9) | 6334(5739 to 7016) | 4.8(4.4 to 5.2) | 6.5(3.3 to 9.6) |  |
| Sao Tome and Principe | 4(3 to 4) | 4.3(4 to 4.7) | 6(6 to 7) | 4.2(3.8 to 4.6) | -2.8(-5.7 to 0.4) |  |
| Senegal | 217(199 to 236) | 4.6(4.2 to 4.9) | 490(446 to 538) | 4.8(4.4 to 5.3) | 5.7(2.1 to 9.2) |  |
| Sierra Leone | 105(95 to 114) | 4(3.7 to 4.3) | 260(234 to 287) | 4.9(4.5 to 5.4) | 23.8(20.4 to 27.7) |  |
| Togo | 92(84 to 101) | 4.2(3.9 to 4.6) | 259(234 to 286) | 4.8(4.4 to 5.2) | 13.9(10.6 to 17.5) |  |
| Eastern Sub-Saharan Africa | 3511(3160 to 3897) | 2.9(2.6 to 3.1) | 7974(7155 to 8856) | 3(2.8 to 3.3) | 5.9(4.1 to 8) |  |
| Burundi | 100(89 to 111) | 2.9(2.6 to 3.1) | 219(196 to 244) | 3.1(2.8 to 3.4) | 8.9(5.6 to 12.2) |  |
| Comoros | 9(8 to 10) | 3(2.8 to 3.3) | 17(16 to 19) | 3(2.7 to 3.3) | -0.9(-3.5 to 1.8) |  |
| Djibouti | 9(8 to 10) | 2.8(2.6 to 3) | 25(22 to 28) | 2.8(2.5 to 3.1) | 0.3(-2.5 to 3.2) |  |
| Eritrea | 52(47 to 58) | 3(2.7 to 3.2) | 123(111 to 137) | 3.1(2.8 to 3.4) | 4.6(2 to 7.3) |  |
| Ethiopia | 922(827 to 1028) | 2.8(2.5 to 3) | 2032(1819 to 2267) | 2.9(2.7 to 3.2) | 5(2.9 to 7.4) |  |
| Kenya | 466(420 to 518) | 3.3(3 to 3.5) | 1171(1056 to 1295) | 3.4(3.1 to 3.8) | 5.5(3.6 to 7.4) |  |
| Madagascar | 199(178 to 222) | 2.5(2.3 to 2.7) | 493(439 to 548) | 2.8(2.5 to 3.1) | 13(10.2 to 16.1) |  |
| Malawi | 178(161 to 197) | 2.8(2.6 to 3.1) | 363(326 to 403) | 3.1(2.8 to 3.4) | 10.3(7.3 to 13.3) |  |
| Mozambique | 286(259 to 316) | 3(2.8 to 3.3) | 606(544 to 676) | 3.2(2.9 to 3.5) | 5.6(2.4 to 9) |  |
| Rwanda | 147(132 to 162) | 3.2(3 to 3.5) | 278(249 to 309) | 3.2(2.9 to 3.5) | -2.3(-5.1 to 0.4) |  |
| Somalia | 124(111 to 137) | 2.7(2.4 to 2.9) | 316(282 to 352) | 2.9(2.7 to 3.2) | 9.3(6.3 to 12.4) |  |
| South Sudan | 107(96 to 119) | 2.8(2.5 to 3) | 184(165 to 204) | 2.9(2.6 to 3.2) | 3.8(1.1 to 6.6) |  |
| Tanzania | 458(412 to 512) | 2.7(2.5 to 3) | 1089(977 to 1207) | 2.9(2.7 to 3.2) | 8.1(5.2 to 11.4) |  |
| Uganda | 303(273 to 339) | 2.9(2.6 to 3.1) | 718(645 to 799) | 3(2.7 to 3.3) | 4.5(2 to 7.1) |  |
| Zambia | 149(134 to 166) | 3.1(2.9 to 3.4) | 334(299 to 371) | 2.9(2.7 to 3.2) | -5.6(-8.5 to -3) |  |
| Central Sub-Saharan Africa | 1085(981 to 1198) | 3(2.8 to 3.3) | 2556(2303 to 2831) | 3.1(2.8 to 3.4) | 2.8(0.5 to 5.1) |  |
| Angola | 198(178 to 220) | 3(2.7 to 3.3) | 560(503 to 620) | 3.1(2.8 to 3.4) | 3.3(1 to 5.7) |  |
| Central African Republic | 61(55 to 67) | 3.4(3.1 to 3.7) | 110(99 to 121) | 3.4(3.1 to 3.7) | -0.6(-3.5 to 2) |  |
| Congo | 49(45 to 54) | 3.1(2.8 to 3.3) | 116(104 to 128) | 3.1(2.8 to 3.4) | 1.7(-1 to 4.5) |  |
| Democratic Republic of the Congo | 746(674 to 826) | 3(2.8 to 3.3) | 1701(1534 to 1887) | 3.1(2.8 to 3.4) | 3.3(0.5 to 6.1) |  |
| Equatorial Guinea | 9(9 to 10) | 3.4(3.1 to 3.7) | 28(25 to 31) | 3.2(2.9 to 3.5) | -5(-7.1 to -2.8) |  |
| Gabon | 21(19 to 23) | 3(2.7 to 3.2) | 41(37 to 45) | 3(2.7 to 3.3) | 0.3(-2.3 to 3.1) |  |
|  |  |  |  |  |  |  |

| **Table S6: YLDs of pancreatitis in 1990 and 2017 for both sexes and percentage change of age-standardised rates(ASR) by location** | | | | | |  |
| --- | --- | --- | --- | --- | --- | --- |
|  | **1990** | | **2017** | | **Percentage change in ASR from 1990 and 2017** |  |
|  | **Numbers (95% UI)** | **Rate (95% UI)** | **Numbers (95% UI)** | **Rate (95% UI)** |  |  |
| Global | 189382(99346 to 317452) | 4.2(2.2 to 6.9) | 364447(186273 to 612755) | 4.5(2.3 to 7.6) | 9.2(5.5 to 12.4) |  |
| High-income North America | 20984(12330 to 32704) | 6.5(3.8 to 10.3) | 31290(18049 to 49920) | 6.6(3.9 to 10.5) | 1.7(-4 to 6.7) |  |
| Canada | 1870(1117 to 2929) | 6(3.6 to 9.4) | 3361(1962 to 5409) | 6.6(3.9 to 10.6) | 10.2(-4.7 to 25.8) |  |
| Greenland | 2(1 to 4) | 4.8(2.9 to 7.3) | 4(2 to 6) | 5.4(3.2 to 8.6) | 13.5(-1.5 to 29.2) |  |
| United States | 19111(11116 to 29986) | 6.6(3.8 to 10.4) | 27925(16155 to 44442) | 6.6(3.9 to 10.5) | 0.8(-5.1 to 5.8) |  |
| Australasia | 805(462 to 1302) | 3.6(2 to 5.7) | 1606(850 to 2679) | 4.2(2.2 to 6.9) | 17.8(2.4 to 31.7) |  |
| Australia | 633(360 to 1012) | 3.3(1.9 to 5.3) | 1278(677 to 2130) | 3.9(2.1 to 6.5) | 17.9(-1 to 35) |  |
| New Zealand | 172(92 to 284) | 4.6(2.5 to 7.6) | 328(174 to 547) | 5.5(2.9 to 9.2) | 19.3(2.2 to 37.3) |  |
| High-income Asia Pacific | 17516(8769 to 30027) | 8.9(4.5 to 15.2) | 24456(12340 to 41094) | 9.1(4.7 to 15.5) | 3(-2.7 to 8.3) |  |
| Brunei | 13(7 to 23) | 6.7(3.3 to 11.6) | 33(16 to 57) | 7.6(3.8 to 13.3) | 14.1(-3.5 to 32.9) |  |
| Japan | 13891(6975 to 23569) | 9.1(4.6 to 15.5) | 17663(8961 to 29383) | 9.5(4.9 to 16) | 3.9(-1 to 9.1) |  |
| Singapore | 215(102 to 380) | 7(3.3 to 12.1) | 528(256 to 925) | 7.9(3.8 to 13.8) | 13.1(-3.4 to 33.7) |  |
| South Korea | 3397(1694 to 5902) | 8.3(4.1 to 14.4) | 6232(3036 to 10794) | 8.6(4.2 to 14.8) | 3.7(-11.8 to 20.8) |  |
| Western Europe | 25708(13374 to 43618) | 5.3(2.8 to 9) | 41460(21090 to 70249) | 6.6(3.3 to 11.3) | 23.8(16.1 to 31.4) |  |
| Andorra | 3(2 to 5) | 4.7(2.4 to 8) | 7(3 to 12) | 5.9(2.9 to 10) | 24.6(4.3 to 47.7) |  |
| Austria | 1071(534 to 1834) | 11(5.5 to 18.8) | 1248(614 to 2167) | 9.7(4.7 to 16.9) | -12(-24.9 to 2) |  |
| Belgium | 1235(598 to 2182) | 9.8(4.7 to 17.3) | 2333(1081 to 4099) | 14.9(6.9 to 26.3) | 52.9(28.9 to 77.3) |  |
| Cyprus | 19(11 to 31) | 2.3(1.3 to 3.7) | 47(25 to 78) | 2.8(1.5 to 4.7) | 21.6(3.7 to 39.3) |  |
| Denmark | 362(177 to 630) | 5.5(2.7 to 9.6) | 626(295 to 1089) | 7.7(3.7 to 13.3) | 40.6(17.2 to 68.7) |  |
| Finland | 555(273 to 964) | 8.8(4.4 to 15.3) | 973(469 to 1672) | 12.6(6.2 to 21.8) | 43.1(21.8 to 65.9) |  |
| France | 3268(1676 to 5540) | 4.7(2.3 to 7.9) | 5073(2507 to 8702) | 5.5(2.7 to 9.5) | 17.2(-3.7 to 40.7) |  |
| Germany | 5201(2715 to 8756) | 4.9(2.6 to 8.3) | 8454(4368 to 14335) | 6.7(3.4 to 11.4) | 35.2(13.3 to 60) |  |
| Greece | 577(280 to 967) | 4.4(2.1 to 7.4) | 935(457 to 1596) | 5.8(2.8 to 10) | 32.3(10 to 56.4) |  |
| Iceland | 8(5 to 14) | 3.1(1.7 to 5.1) | 19(10 to 32) | 4.5(2.3 to 7.6) | 46.4(19.1 to 72.9) |  |
| Ireland | 154(84 to 262) | 4.1(2.2 to 7) | 344(175 to 579) | 5.6(2.8 to 9.3) | 34.7(12.1 to 59.7) |  |
| Israel | 190(99 to 315) | 4(2.1 to 6.7) | 539(274 to 923) | 5.6(2.8 to 9.6) | 38.5(15.5 to 66.9) |  |
| Italy | 3181(1654 to 5441) | 4.3(2.2 to 7.3) | 4511(2326 to 7508) | 4.6(2.4 to 7.7) | 7.8(-10.4 to 30.9) |  |
| Luxembourg | 25(13 to 43) | 5.2(2.6 to 8.7) | 50(25 to 86) | 6.2(3.1 to 10.6) | 19.7(0.9 to 44.5) |  |
| Malta | 10(5 to 17) | 2.4(1.2 to 4) | 18(10 to 31) | 2.8(1.4 to 4.7) | 16.2(-3.2 to 38.2) |  |
| Netherlands | 629(294 to 1123) | 3.5(1.6 to 6.2) | 1085(522 to 1899) | 4.5(2.1 to 7.8) | 28.9(3.1 to 59.7) |  |
| Norway | 478(246 to 804) | 9.1(4.7 to 15.6) | 767(395 to 1291) | 10.6(5.5 to 17.9) | 16(7.8 to 24.6) |  |
| Portugal | 562(286 to 975) | 4.6(2.4 to 8.1) | 934(473 to 1609) | 5.7(2.9 to 9.9) | 22.1(1.3 to 46.4) |  |
| Spain | 2401(1192 to 4243) | 5.1(2.5 to 9) | 4227(2055 to 7503) | 6(2.8 to 10.7) | 18.9(-0.9 to 41.4) |  |
| Sweden | 535(280 to 882) | 4.7(2.5 to 7.8) | 783(407 to 1299) | 5.5(2.9 to 9.3) | 17.1(1.5 to 36.4) |  |
| Switzerland | 316(170 to 541) | 3.5(1.9 to 6) | 522(273 to 885) | 4.1(2.2 to 7) | 16.5(-3.6 to 39.7) |  |
| United Kingdom | 4900(2501 to 8238) | 6.8(3.5 to 11.4) | 7922(3957 to 13528) | 8.6(4.3 to 14.8) | 27.8(22.9 to 32.7) |  |
| Southern Latin America | 1289(717 to 2133) | 2.7(1.5 to 4.5) | 2090(1145 to 3364) | 2.8(1.5 to 4.5) | 3.3(-8.6 to 16.3) |  |
| Argentina | 891(488 to 1483) | 2.7(1.5 to 4.6) | 1367(757 to 2181) | 2.8(1.5 to 4.5) | 2.1(-14 to 20.1) |  |
| Chile | 316(179 to 519) | 2.7(1.5 to 4.4) | 608(337 to 999) | 2.8(1.6 to 4.6) | 4.2(-11.8 to 22.5) |  |
| Uruguay | 83(45 to 134) | 2.4(1.3 to 3.8) | 114(62 to 188) | 2.7(1.4 to 4.4) | 12.8(-4.4 to 30.9) |  |
| Eastern Europe | 21828(11119 to 37354) | 8.3(4.2 to 14.3) | 34940(17606 to 60267) | 12.3(6.1 to 21.2) | 47.5(40.3 to 54) |  |
| Belarus | 985(497 to 1669) | 8.2(4.2 to 13.8) | 1458(739 to 2533) | 11.1(5.6 to 19.3) | 35.4(16.9 to 56.2) |  |
| Estonia | 160(82 to 270) | 8.6(4.4 to 14.6) | 195(98 to 335) | 10.4(5.2 to 18) | 20.6(3 to 37.8) |  |
| Latvia | 287(144 to 486) | 8.8(4.4 to 15.1) | 318(161 to 544) | 11.3(5.7 to 19.8) | 28.6(11.1 to 50.2) |  |
| Lithuania | 352(175 to 587) | 8.2(4.1 to 13.7) | 511(258 to 849) | 12.5(6.2 to 21) | 51.7(31.9 to 72.8) |  |
| Moldova | 590(296 to 1023) | 12.8(6.4 to 22.2) | 535(272 to 916) | 10.9(5.6 to 18.8) | -14.5(-26.3 to -1.5) |  |
| Russian Federation | 14656(7463 to 24963) | 8.5(4.3 to 14.4) | 25450(12670 to 43726) | 13(6.4 to 22.4) | 52.8(45.8 to 59) |  |
| Ukraine | 4799(2456 to 8045) | 7.6(3.9 to 12.8) | 6474(3248 to 10948) | 10.6(5.3 to 17.9) | 39.8(22.2 to 59.6) |  |
| Central Europe | 15063(7572 to 25680) | 10.5(5.3 to 18) | 21277(10479 to 36432) | 12.5(6.1 to 21.4) | 19(11.5 to 26.6) |  |
| Albania | 189(99 to 315) | 7.2(3.7 to 12) | 348(174 to 594) | 9.7(4.9 to 16.5) | 34.8(16.2 to 56.5) |  |
| Bosnia and Herzegovina | 349(177 to 588) | 7.5(3.8 to 12.7) | 505(259 to 866) | 10.1(5.2 to 17.5) | 34.4(16.8 to 53.8) |  |
| Bulgaria | 860(433 to 1437) | 7.5(3.8 to 12.6) | 1125(565 to 1911) | 10.1(5 to 17.3) | 34.4(16.3 to 56) |  |
| Croatia | 542(274 to 901) | 8.9(4.6 to 14.7) | 573(290 to 962) | 8.6(4.4 to 14.5) | -3.2(-16.5 to 11.6) |  |
| Czech Republic | 1453(743 to 2518) | 11.5(5.9 to 19.9) | 2133(1071 to 3642) | 13.1(6.6 to 22.5) | 14.3(0.1 to 30.3) |  |
| Hungary | 1523(753 to 2652) | 11.4(5.6 to 19.9) | 1565(782 to 2670) | 10.5(5.2 to 17.8) | -8.2(-20.1 to 5.5) |  |
| Macedonia | 153(77 to 265) | 7.5(3.8 to 13) | 294(142 to 498) | 9.9(4.9 to 16.9) | 32(14.1 to 50.9) |  |
| Montenegro | 52(27 to 87) | 7.9(4.1 to 13.4) | 81(40 to 136) | 9.6(4.8 to 16) | 20.6(4.6 to 40.1) |  |
| Poland | 5362(2676 to 9232) | 12.3(6.1 to 21.1) | 8324(4121 to 14375) | 14.9(7.3 to 25.9) | 21.1(7.1 to 37.2) |  |
| Romania | 2577(1314 to 4404) | 9.6(4.9 to 16.5) | 3532(1736 to 6044) | 12.1(5.9 to 20.7) | 25.4(9.3 to 42.7) |  |
| Serbia | 943(470 to 1593) | 8.4(4.2 to 14.3) | 1217(595 to 2057) | 9.5(4.6 to 16) | 13(-2.3 to 29.6) |  |
| Slovakia | 832(421 to 1449) | 14.4(7.2 to 25) | 1285(635 to 2213) | 16.6(8.2 to 28.6) | 15.7(3.9 to 30.2) |  |
| Slovenia | 229(116 to 387) | 9.6(4.9 to 16.2) | 293(147 to 502) | 9(4.5 to 15.5) | -6.2(-18.4 to 8.5) |  |
| Central Asia | 3407(1637 to 6008) | 6.2(3 to 10.8) | 6067(2856 to 10616) | 7(3.3 to 12.2) | 13.1(3.7 to 23) |  |
| Armenia | 148(71 to 265) | 4.8(2.3 to 8.6) | 238(114 to 414) | 6.4(3.1 to 11.2) | 33.8(10.6 to 58.4) |  |
| Azerbaijan | 275(129 to 479) | 4.5(2.1 to 7.9) | 662(321 to 1190) | 6.2(3 to 11) | 36.4(12.9 to 64.8) |  |
| Georgia | 287(135 to 506) | 4.8(2.3 to 8.4) | 299(143 to 516) | 6.1(2.9 to 10.7) | 28.1(7.7 to 52.2) |  |
| Kazakhstan | 1457(700 to 2689) | 9.9(4.7 to 18.2) | 1816(840 to 3214) | 9.8(4.5 to 17.2) | -1.1(-15.1 to 16.5) |  |
| Kyrgyzstan | 169(80 to 297) | 4.8(2.3 to 8.6) | 328(158 to 572) | 5.9(2.8 to 10.4) | 22.9(1.5 to 49.8) |  |
| Mongolia | 94(45 to 166) | 6.8(3.3 to 12.1) | 228(107 to 412) | 7.6(3.6 to 13.4) | 11.1(-5 to 31.2) |  |
| Tajikistan | 172(81 to 300) | 4.7(2.2 to 8.2) | 451(206 to 784) | 6.3(2.9 to 10.8) | 32.7(10.7 to 58.6) |  |
| Turkmenistan | 117(57 to 209) | 4.5(2.2 to 8) | 284(134 to 509) | 6.1(2.9 to 11) | 36.5(12.9 to 63.1) |  |
| Uzbekistan | 688(325 to 1208) | 4.6(2.2 to 8.1) | 1761(817 to 3161) | 6.1(2.8 to 10.8) | 32.4(10.6 to 57.6) |  |
| Central Latin America | 4510(2357 to 7566) | 4(2.1 to 6.6) | 11472(5968 to 19250) | 4.7(2.4 to 7.9) | 17.9(11.9 to 23.7) |  |
| Colombia | 884(455 to 1510) | 3.8(1.9 to 6.6) | 2514(1291 to 4283) | 4.7(2.4 to 8) | 23.8(6.6 to 43.4) |  |
| Costa Rica | 107(57 to 180) | 4.9(2.6 to 8.2) | 277(141 to 474) | 5.6(2.9 to 9.6) | 14.1(-2.5 to 31.8) |  |
| El Salvador | 139(73 to 234) | 3.8(2 to 6.4) | 271(136 to 454) | 4.6(2.3 to 7.8) | 22.3(5 to 39.3) |  |
| Guatemala | 214(112 to 371) | 4.2(2.2 to 7.3) | 617(312 to 1054) | 4.8(2.4 to 8.1) | 13.5(-3.2 to 33.8) |  |
| Honduras | 109(57 to 183) | 3.8(2 to 6.3) | 345(181 to 584) | 4.9(2.5 to 8.3) | 29.2(9.4 to 50.9) |  |
| Mexico | 2425(1279 to 4047) | 4.1(2.2 to 6.9) | 5680(2986 to 9448) | 4.7(2.5 to 7.8) | 13.5(8.1 to 18.9) |  |
| Nicaragua | 84(44 to 141) | 3.7(1.9 to 6) | 237(122 to 400) | 4.5(2.3 to 7.6) | 22.9(5 to 42.7) |  |
| Panama | 68(36 to 114) | 3.7(2 to 6.2) | 185(93 to 308) | 4.6(2.3 to 7.7) | 25(7.6 to 42.4) |  |
| Venezuela | 479(251 to 800) | 3.7(1.9 to 6) | 1346(677 to 2308) | 4.6(2.3 to 7.8) | 24.7(6.3 to 44) |  |
| Andean Latin America | 898(478 to 1497) | 3.4(1.8 to 5.7) | 2370(1211 to 3990) | 4.2(2.1 to 7) | 22.5(9.6 to 35.4) |  |
| Bolivia | 129(68 to 217) | 3.1(1.6 to 5.2) | 400(199 to 693) | 4.2(2.1 to 7.2) | 35.9(14.6 to 59.3) |  |
| Ecuador | 282(144 to 479) | 4.2(2.1 to 7.1) | 629(328 to 1065) | 4.1(2.1 to 6.9) | -1.9(-15.7 to 14.7) |  |
| Peru | 487(259 to 826) | 3.2(1.7 to 5.4) | 1340(675 to 2293) | 4.2(2.1 to 7.2) | 33.4(11.8 to 57.7) |  |
| Caribbean | 1032(517 to 1723) | 3.6(1.8 to 6) | 2438(1192 to 4177) | 4.9(2.4 to 8.3) | 35.5(24.9 to 45.4) |  |
| Antigua and Barbuda | 2(1 to 3) | 3.6(1.8 to 6.3) | 5(2 to 8) | 4.8(2.3 to 8.1) | 32.3(14.4 to 52) |  |
| The Bahamas | 8(4 to 13) | 4.2(2 to 7.1) | 20(10 to 35) | 5.3(2.6 to 9) | 25.9(8.2 to 46) |  |
| Barbados | 11(5 to 18) | 3.7(1.9 to 6.4) | 21(10 to 36) | 4.9(2.4 to 8.4) | 30.7(12.1 to 50.4) |  |
| Belize | 4(2 to 6) | 3.1(1.5 to 5.3) | 13(7 to 23) | 4.4(2.1 to 7.6) | 39.8(18.8 to 61.5) |  |
| Bermuda | 3(1 to 5) | 4.2(2.1 to 7.1) | 6(3 to 10) | 5.2(2.5 to 8.8) | 23.4(5.3 to 42.2) |  |
| Cuba | 397(199 to 678) | 3.7(1.9 to 6.3) | 878(422 to 1519) | 5.2(2.6 to 9) | 40.8(21.2 to 61.4) |  |
| Dominica | 3(1 to 5) | 3.7(1.9 to 6.4) | 4(2 to 7) | 4.9(2.4 to 8.5) | 33.1(12.9 to 53.6) |  |
| Dominican Republic | 147(74 to 249) | 3(1.5 to 5.1) | 439(214 to 754) | 4.5(2.2 to 7.7) | 48.9(28.4 to 70.3) |  |
| Grenada | 2(1 to 4) | 3.2(1.7 to 5.5) | 6(3 to 10) | 4.3(2.1 to 7.4) | 31.9(10.7 to 52.4) |  |
| Guyana | 17(9 to 29) | 3.3(1.7 to 5.8) | 30(15 to 51) | 4.6(2.3 to 7.7) | 36.7(15 to 60.1) |  |
| Haiti | 113(57 to 190) | 2.8(1.4 to 4.7) | 337(164 to 573) | 4.1(2 to 6.9) | 46.2(26.6 to 67.3) |  |
| Jamaica | 63(32 to 109) | 3.3(1.6 to 5.7) | 131(65 to 227) | 4.4(2.2 to 7.7) | 35(15 to 55.3) |  |
| Puerto Rico | 175(86 to 293) | 4.7(2.3 to 8) | 334(162 to 577) | 5.5(2.7 to 9.5) | 16.2(1.1 to 32.4) |  |
| Saint Lucia | 3(2 to 5) | 3(1.5 to 5.3) | 9(4 to 15) | 4.4(2.1 to 7.4) | 43.4(22.9 to 65.1) |  |
| Saint Vincent and the Grenadines | 3(1 to 5) | 3.6(1.8 to 6.1) | 6(3 to 11) | 4.7(2.3 to 8.2) | 30.2(11.1 to 51.7) |  |
| Suriname | 10(5 to 17) | 3.5(1.7 to 5.9) | 28(14 to 49) | 4.8(2.4 to 8.3) | 37.8(17.2 to 60) |  |
| Trinidad and Tobago | 32(17 to 55) | 3.4(1.7 to 5.7) | 77(38 to 132) | 4.6(2.3 to 7.9) | 37(16.9 to 58.3) |  |
| Virgin Islands, U.S. | 3(2 to 6) | 3.6(1.8 to 6) | 8(4 to 13) | 4.9(2.4 to 8.5) | 38.8(18.3 to 60.8) |  |
| Tropical Latin America | 10648(5082 to 18654) | 9.6(4.6 to 17) | 20701(9974 to 35370) | 8.9(4.3 to 15.2) | -7.8(-12.4 to -2.8) |  |
| Brazil | 10480(4997 to 18346) | 9.7(4.7 to 17.1) | 20238(9764 to 34554) | 8.9(4.3 to 15.3) | -8.3(-13 to -3.3) |  |
| Paraguay | 168(84 to 293) | 6.4(3.1 to 11.1) | 464(218 to 796) | 8.1(3.8 to 14) | 27.5(10 to 46.8) |  |
| East Asia | 31096(17226 to 50397) | 3(1.6 to 4.8) | 73112(39225 to 120354) | 3.7(2 to 6.2) | 26.7(20.4 to 31.9) |  |
| China | 29821(16514 to 48373) | 3(1.6 to 4.8) | 69745(37445 to 114781) | 3.8(2 to 6.2) | 25.8(19.6 to 31.1) |  |
| North Korea | 396(223 to 648) | 2.2(1.2 to 3.6) | 956(517 to 1581) | 3.1(1.7 to 5.1) | 41.6(22.1 to 62.9) |  |
| Taiwan (Province of China) | 361(205 to 574) | 2(1.1 to 3.2) | 1233(640 to 2090) | 3.6(1.8 to 6) | 77.4(48.5 to 104.7) |  |
| Southeast Asia | 7413(3855 to 12433) | 2.2(1.2 to 3.7) | 18788(9616 to 31897) | 2.9(1.5 to 4.9) | 30(22.4 to 36.8) |  |
| Cambodia | 126(65 to 210) | 2(1 to 3.4) | 351(178 to 593) | 2.7(1.4 to 4.5) | 30.8(8.5 to 54.6) |  |
| Indonesia | 3245(1702 to 5456) | 2.5(1.3 to 4.2) | 7447(3831 to 12817) | 3.1(1.6 to 5.2) | 23.1(14.9 to 31.1) |  |
| Laos | 53(28 to 91) | 2(1 to 3.3) | 145(73 to 247) | 2.7(1.4 to 4.7) | 38.8(16.6 to 65.1) |  |
| Malaysia | 297(152 to 504) | 2.5(1.3 to 4.2) | 955(472 to 1597) | 3.4(1.7 to 5.7) | 36.8(15.1 to 60.9) |  |
| Maldives | 2(1 to 4) | 1.8(0.9 to 3.1) | 11(6 to 19) | 2.9(1.4 to 4.9) | 57.6(33 to 84.4) |  |
| Mauritius | 30(15 to 51) | 3.1(1.6 to 5.3) | 54(27 to 94) | 3.4(1.7 to 5.9) | 7.9(-12.6 to 32.3) |  |
| Myanmar | 526(272 to 893) | 1.8(0.9 to 3) | 1242(629 to 2157) | 2.5(1.3 to 4.3) | 39.4(18.9 to 59.2) |  |
| Philippines | 841(435 to 1448) | 2(1 to 3.5) | 2307(1186 to 3961) | 2.7(1.4 to 4.6) | 34.1(14.3 to 57.1) |  |
| Sri Lanka | 251(131 to 429) | 1.8(1 to 3.1) | 643(319 to 1088) | 2.7(1.3 to 4.5) | 44.8(26 to 65.3) |  |
| Seychelles | 2(1 to 3) | 2.5(1.3 to 4.2) | 4(2 to 6) | 3.2(1.6 to 5.5) | 28.4(5.4 to 50.7) |  |
| Thailand | 910(473 to 1556) | 2(1 to 3.4) | 2712(1380 to 4649) | 2.9(1.5 to 4.9) | 43.7(20.5 to 67.3) |  |
| Timor-Leste | 10(5 to 17) | 2.1(1.1 to 3.6) | 27(13 to 47) | 3(1.5 to 5.1) | 40.1(16.3 to 66.5) |  |
| Vietnam | 1111(576 to 1834) | 2.3(1.2 to 3.9) | 2865(1456 to 4975) | 2.9(1.5 to 5) | 24.2(2.4 to 47.9) |  |
| Oceania | 62(33 to 105) | 1.5(0.8 to 2.5) | 173(90 to 294) | 1.9(1 to 3.3) | 30.3(21.6 to 38.4) |  |
| American Samoa | 1(0 to 1) | 2.1(1.1 to 3.5) | 1(1 to 2) | 2.7(1.4 to 4.5) | 26.1(7.4 to 47) |  |
| Federated States of Micronesia | 1(1 to 2) | 1.8(1 to 3) | 2(1 to 3) | 2.2(1.1 to 3.8) | 22.1(7.4 to 38.9) |  |
| Fiji | 9(5 to 15) | 1.7(0.9 to 2.8) | 17(9 to 30) | 2.1(1.1 to 3.5) | 25.7(11.2 to 39.9) |  |
| Guam | 2(1 to 3) | 1.9(1 to 3.2) | 4(2 to 7) | 2.4(1.2 to 3.9) | 24.1(8.8 to 40) |  |
| Kiribati | 1(0 to 2) | 1.8(1 to 3) | 2(1 to 3) | 2.2(1.2 to 3.7) | 22.4(8.7 to 38.7) |  |
| Marshall Islands | 0(0 to 1) | 1.8(0.9 to 3) | 1(0 to 2) | 2.2(1.2 to 3.7) | 23.8(7.7 to 39.6) |  |
| Northern Mariana Islands | 1(0 to 1) | 2.1(1.1 to 3.5) | 1(1 to 2) | 2.6(1.3 to 4.3) | 24.6(8.4 to 42.2) |  |
| Papua New Guinea | 35(19 to 60) | 1.3(0.7 to 2.2) | 115(59 to 196) | 1.8(0.9 to 3) | 37.3(24.4 to 49.9) |  |
| Samoa | 2(1 to 3) | 1.9(1 to 3.1) | 4(2 to 6) | 2.3(1.2 to 3.9) | 24.1(6.4 to 41.1) |  |
| Solomon Islands | 3(2 to 5) | 1.5(0.8 to 2.5) | 9(4 to 15) | 1.9(1 to 3.3) | 32.3(19.5 to 44.5) |  |
| Tonga | 1(1 to 2) | 2.1(1.1 to 3.6) | 2(1 to 4) | 2.6(1.4 to 4.3) | 23.1(4.6 to 43.1) |  |
| Vanuatu | 2(1 to 3) | 2(1.1 to 3.3) | 5(3 to 8) | 2.3(1.2 to 4) | 16.8(2 to 33.2) |  |
| North Africa and Middle East | 5373(2710 to 9243) | 2.3(1.2 to 3.9) | 14629(7146 to 25120) | 2.8(1.4 to 4.8) | 22.8(15.6 to 29.4) |  |
| Afghanistan | 162(82 to 280) | 2.1(1.1 to 3.7) | 468(230 to 809) | 2.5(1.2 to 4.2) | 18.3(1.3 to 37.6) |  |
| Algeria | 406(199 to 700) | 2.4(1.2 to 4.2) | 1085(543 to 1881) | 2.8(1.4 to 4.9) | 17.6(-2 to 39.4) |  |
| Bahrain | 10(5 to 17) | 2.8(1.4 to 4.8) | 47(23 to 83) | 3.1(1.6 to 5.3) | 9.7(-8.4 to 30.4) |  |
| Egypt | 893(449 to 1550) | 2.3(1.1 to 3.9) | 2153(1045 to 3766) | 2.8(1.4 to 4.9) | 24.3(5.3 to 45.8) |  |
| Iran | 774(397 to 1334) | 2.1(1.1 to 3.5) | 2234(1105 to 3810) | 2.7(1.4 to 4.6) | 32.5(26.7 to 38.8) |  |
| Iraq | 244(121 to 418) | 2.2(1.1 to 3.8) | 821(401 to 1445) | 2.6(1.3 to 4.5) | 15.9(-1.8 to 36) |  |
| Jordan | 80(39 to 139) | 3.6(1.7 to 6.1) | 389(192 to 698) | 4.6(2.3 to 8.3) | 30.7(8.2 to 55.8) |  |
| Kuwait | 34(17 to 60) | 2.7(1.3 to 4.6) | 123(60 to 217) | 3(1.5 to 5.2) | 12.5(-7.4 to 33.1) |  |
| Lebanon | 72(37 to 125) | 2.5(1.3 to 4.3) | 217(105 to 372) | 3(1.5 to 5.2) | 20.7(1.9 to 41.8) |  |
| Libya | 68(34 to 115) | 2.5(1.3 to 4.3) | 181(89 to 316) | 3(1.4 to 5.2) | 17.7(-2.6 to 39.2) |  |
| Morocco | 431(213 to 742) | 2.3(1.1 to 4) | 982(465 to 1700) | 2.8(1.3 to 4.9) | 20.9(1.3 to 42.3) |  |
| Palestine | 31(16 to 53) | 2.6(1.3 to 4.5) | 98(49 to 170) | 2.9(1.4 to 5) | 10.6(-7.7 to 31.9) |  |
| Oman | 29(14 to 50) | 2.3(1.2 to 4) | 106(51 to 188) | 2.8(1.4 to 4.9) | 22.3(1.8 to 46) |  |
| Qatar | 9(5 to 16) | 2.7(1.4 to 4.7) | 74(35 to 129) | 3(1.4 to 5.3) | 10.7(-9.1 to 33.6) |  |
| Saudi Arabia | 266(134 to 453) | 2.6(1.3 to 4.5) | 905(439 to 1592) | 3.1(1.5 to 5.2) | 16.4(-3.2 to 37) |  |
| Sudan | 284(139 to 497) | 2.2(1 to 3.8) | 702(349 to 1215) | 2.7(1.3 to 4.7) | 23.1(4 to 45.2) |  |
| Syria | 187(93 to 318) | 2.4(1.2 to 4.2) | 441(212 to 762) | 2.9(1.4 to 4.9) | 17.1(-1.1 to 40.6) |  |
| Tunisia | 158(77 to 268) | 2.5(1.2 to 4.3) | 373(183 to 645) | 3(1.5 to 5.1) | 18.7(1.2 to 39.9) |  |
| Turkey | 1028(516 to 1764) | 2.3(1.2 to 4) | 2400(1197 to 4130) | 2.7(1.3 to 4.7) | 17(-1.4 to 39.6) |  |
| United Arab Emirates | 33(16 to 57) | 2.4(1.2 to 4) | 294(142 to 513) | 2.9(1.4 to 4.9) | 20.1(-1.4 to 44.4) |  |
| Yemen | 171(83 to 296) | 2.2(1 to 3.8) | 522(255 to 919) | 2.7(1.3 to 4.7) | 23.1(5.3 to 43.8) |  |
| South Asia | 17910(8886 to 31327) | 2.1(1 to 3.6) | 48453(23785 to 85613) | 2.9(1.4 to 5.1) | 41.4(35.8 to 47.3) |  |
| Bangladesh | 1523(758 to 2682) | 2(1 to 3.6) | 3648(1735 to 6399) | 2.5(1.2 to 4.4) | 23.4(5.6 to 42.2) |  |
| Bhutan | 9(4 to 16) | 2.3(1.1 to 4) | 23(11 to 40) | 2.7(1.3 to 4.7) | 15.2(-6.1 to 40.2) |  |
| India | 14322(7068 to 25050) | 2.1(1 to 3.6) | 39872(19667 to 70403) | 3(1.5 to 5.3) | 47.9(42.1 to 53.9) |  |
| Nepal | 303(149 to 520) | 2.1(1.1 to 3.7) | 640(313 to 1092) | 2.5(1.2 to 4.2) | 14.4(-4.2 to 35.9) |  |
| Pakistan | 1753(851 to 3056) | 2.3(1.1 to 3.9) | 4269(2014 to 7428) | 2.6(1.2 to 4.4) | 13.2(-7.1 to 38.2) |  |
| Southern Sub-Saharan Africa | 412(190 to 732) | 1.1(0.5 to 1.9) | 768(355 to 1375) | 1.1(0.5 to 2) | 5.2(2.4 to 7.8) |  |
| Botswana | 9(4 to 17) | 1.1(0.5 to 2) | 22(10 to 40) | 1.2(0.5 to 2) | 4.4(0.7 to 8.1) |  |
| Lesotho | 14(6 to 24) | 1.1(0.5 to 1.9) | 17(8 to 30) | 1.1(0.5 to 1.9) | 1.1(-2.6 to 4.7) |  |
| Namibia | 10(5 to 18) | 1.1(0.5 to 1.9) | 22(10 to 39) | 1.2(0.5 to 2.1) | 9.7(5.7 to 13.6) |  |
| South Africa | 309(143 to 549) | 1.1(0.5 to 1.9) | 588(272 to 1055) | 1.1(0.5 to 2) | 4.8(2 to 7.5) |  |
| Swaziland | 5(2 to 9) | 1(0.5 to 1.8) | 9(4 to 16) | 1.1(0.5 to 1.9) | 8.9(4.5 to 13) |  |
| Zimbabwe | 65(30 to 115) | 1(0.5 to 1.8) | 110(51 to 196) | 1.1(0.5 to 1.9) | 5.4(1.7 to 9.5) |  |
| Western Sub-Saharan Africa | 1914(888 to 3395) | 1.5(0.7 to 2.7) | 4652(2184 to 8343) | 1.7(0.8 to 3) | 9.2(1.9 to 18) |  |
| Benin | 42(19 to 75) | 1.5(0.7 to 2.6) | 122(56 to 219) | 1.7(0.8 to 3) | 15.2(2.2 to 31.5) |  |
| Burkina Faso | 81(37 to 144) | 1.4(0.6 to 2.4) | 215(97 to 388) | 1.6(0.7 to 2.9) | 18.6(5.1 to 36.3) |  |
| Cameroon | 103(48 to 184) | 1.6(0.7 to 2.8) | 299(136 to 536) | 1.7(0.7 to 2.9) | 5(-12.4 to 24.3) |  |
| Cape Verde | 4(2 to 7) | 1.5(0.7 to 2.8) | 9(4 to 16) | 1.8(0.8 to 3.3) | 17.7(-1 to 40.1) |  |
| Chad | 55(26 to 98) | 1.5(0.7 to 2.6) | 136(62 to 241) | 1.6(0.7 to 2.8) | 8.2(-2.9 to 21.6) |  |
| Cote d'Ivoire | 105(48 to 188) | 1.4(0.6 to 2.5) | 276(123 to 502) | 1.6(0.7 to 2.9) | 13.5(0.7 to 29.2) |  |
| The Gambia | 10(4 to 17) | 1.6(0.8 to 2.9) | 24(11 to 42) | 1.7(0.8 to 3) | 4(-15.1 to 26.5) |  |
| Ghana | 144(67 to 257) | 1.5(0.7 to 2.6) | 363(168 to 646) | 1.6(0.8 to 2.9) | 8.1(-5.5 to 25.1) |  |
| Guinea | 59(27 to 105) | 1.4(0.6 to 2.5) | 118(55 to 210) | 1.6(0.7 to 2.8) | 11.4(0.1 to 24.8) |  |
| Guinea-Bissau | 9(4 to 16) | 1.4(0.7 to 2.6) | 18(8 to 32) | 1.5(0.7 to 2.7) | 6.2(-5.9 to 20) |  |
| Liberia | 19(9 to 34) | 1.4(0.6 to 2.4) | 52(24 to 92) | 1.6(0.7 to 2.9) | 18.1(9.6 to 29.9) |  |
| Mali | 78(36 to 138) | 1.4(0.6 to 2.4) | 206(92 to 373) | 1.7(0.8 to 3.1) | 23.4(7.7 to 42.2) |  |
| Mauritania | 22(10 to 39) | 1.6(0.7 to 2.8) | 46(21 to 82) | 1.7(0.8 to 3.1) | 7.5(-9.8 to 27.6) |  |
| Niger | 71(33 to 127) | 1.5(0.7 to 2.7) | 198(92 to 359) | 1.7(0.8 to 3.1) | 12.1(-0.8 to 27.8) |  |
| Nigeria | 973(445 to 1735) | 1.6(0.7 to 2.8) | 2221(1024 to 3987) | 1.7(0.8 to 3) | 6.5(-6.2 to 21.7) |  |
| Sao Tome and Principe | 1(1 to 2) | 1.6(0.7 to 2.9) | 2(1 to 4) | 1.6(0.8 to 3) | 4.6(-11.5 to 24.1) |  |
| Senegal | 73(35 to 129) | 1.6(0.7 to 2.8) | 172(80 to 305) | 1.7(0.8 to 3) | 8.7(-9.3 to 27.8) |  |
| Sierra Leone | 34(16 to 61) | 1.3(0.6 to 2.4) | 86(40 to 156) | 1.7(0.8 to 2.9) | 24.1(8.8 to 42.8) |  |
| Togo | 30(14 to 54) | 1.4(0.6 to 2.5) | 89(40 to 155) | 1.7(0.8 to 2.9) | 18(2.6 to 35.6) |  |
| Eastern Sub-Saharan Africa | 1152(536 to 2058) | 1(0.4 to 1.7) | 2799(1291 to 5030) | 1.1(0.5 to 2) | 15.3(12.8 to 17.9) |  |
| Burundi | 31(14 to 55) | 0.9(0.4 to 1.6) | 72(33 to 129) | 1(0.5 to 1.9) | 16.7(12.3 to 20.8) |  |
| Comoros | 3(2 to 6) | 1.1(0.5 to 1.9) | 7(3 to 12) | 1.2(0.5 to 2.1) | 7.5(3.7 to 11.1) |  |
| Djibouti | 3(1 to 6) | 1.1(0.5 to 1.9) | 10(5 to 18) | 1.2(0.5 to 2.1) | 9.8(5.9 to 13.6) |  |
| Eritrea | 17(8 to 30) | 1(0.4 to 1.7) | 42(20 to 76) | 1.1(0.5 to 1.9) | 13.2(9.6 to 16.6) |  |
| Ethiopia | 290(136 to 517) | 0.9(0.4 to 1.6) | 709(331 to 1276) | 1.1(0.5 to 1.9) | 21.3(18.3 to 24) |  |
| Kenya | 156(73 to 279) | 1.2(0.5 to 2.1) | 414(192 to 745) | 1.3(0.6 to 2.3) | 8.8(6.4 to 11.2) |  |
| Madagascar | 73(34 to 130) | 0.9(0.4 to 1.7) | 188(87 to 336) | 1.1(0.5 to 2) | 16.9(13.5 to 20.1) |  |
| Malawi | 58(27 to 103) | 0.9(0.4 to 1.7) | 123(57 to 219) | 1.1(0.5 to 2) | 17.8(13.8 to 21.6) |  |
| Mozambique | 92(43 to 166) | 1(0.5 to 1.8) | 206(95 to 370) | 1.1(0.5 to 2) | 15.4(11 to 20.1) |  |
| Rwanda | 46(21 to 82) | 1(0.5 to 1.8) | 97(45 to 174) | 1.1(0.5 to 2) | 11.5(7.2 to 16.3) |  |
| Somalia | 43(20 to 77) | 0.9(0.4 to 1.7) | 111(51 to 198) | 1(0.5 to 1.8) | 12.5(9.1 to 16.2) |  |
| South Sudan | 38(18 to 68) | 1(0.5 to 1.8) | 68(31 to 122) | 1.1(0.5 to 1.9) | 7.7(4 to 11.2) |  |
| Tanzania | 156(72 to 278) | 1(0.4 to 1.7) | 391(180 to 700) | 1.1(0.5 to 2) | 16(12.3 to 20.2) |  |
| Uganda | 97(45 to 175) | 0.9(0.4 to 1.7) | 244(113 to 441) | 1.1(0.5 to 1.9) | 13.5(9.9 to 17) |  |
| Zambia | 47(22 to 84) | 1(0.5 to 1.8) | 114(53 to 204) | 1(0.5 to 1.8) | 3.2(-0.6 to 7) |  |
| Central Sub-Saharan Africa | 363(167 to 649) | 1(0.5 to 1.8) | 907(417 to 1622) | 1.1(0.5 to 2) | 9.9(7 to 12.7) |  |
| Angola | 67(31 to 120) | 1(0.5 to 1.8) | 198(92 to 354) | 1.1(0.5 to 2) | 10.6(7.4 to 14) |  |
| Central African Republic | 18(8 to 32) | 1(0.4 to 1.8) | 34(16 to 61) | 1(0.5 to 1.8) | 3(-0.9 to 6.7) |  |
| Congo | 16(7 to 28) | 1(0.5 to 1.7) | 42(19 to 75) | 1.1(0.5 to 2) | 11.6(7.8 to 15.2) |  |
| Democratic Republic of the Congo | 251(117 to 449) | 1(0.5 to 1.8) | 607(279 to 1087) | 1.1(0.5 to 2) | 10(6.6 to 13.3) |  |
| Equatorial Guinea | 3(1 to 5) | 1.1(0.5 to 1.9) | 10(5 to 18) | 1.2(0.6 to 2.2) | 14.8(10.5 to 18.6) |  |
| Gabon | 7(3 to 13) | 1(0.5 to 1.8) | 16(7 to 28) | 1.1(0.5 to 2) | 10.8(6.9 to 14.4) |  |
|  |  |  |  |  |  |  |
