# Supplementary material for: The global, regional, and national burden of pancreatitis in 195 countries and territories, 1990–2017: a systematic analysis for the Global Burden of Disease Study 2017
Source: BMC Med. 2020 Dec 10;18:388. doi: 10.1186/s12916-020-01859-5 (PMC7726906; doi:10.1186/s12916-020-01859-5)
Supplement: Supplementary file 2 — Additional file 2: Figure S1. The percentage change in age-standardized point prevalence of pancreatitis from 1990 to 2017 for 21 Global Burden of Disease regions by sex. Figure S2. Number of prevalent cases of pancreatitis from 1990 to 2017 for 21 Global Burden of Disease regions. Figure S3. The percentage change in age-standardized point incidence of pancreatitis from 1990 to 2017 for 21 Global Burden of Disease regions by sex. Figure S4. Number of incident cases of pancreatitis from 1990 to 2017 for 21 Global Burden of Disease regions. Figure S5. The age-standardized YLDs of pancreatitis in 2017 for 21 GBD regions, by sex. Figure S6. The percentage change in age-standardized point YLDs of pancreatitis from 1990 to 2017 for 21 Global Burden of Disease regions by sex. Figure S7. Age-standardized YLDs rates of pancreatitis per 100,000 population in 2017, by country and territory. Figure S8. Global cases and age-standardized rates of incidence of pancreatitis per 100,000 population by age and sex, 2017. Shading indicates the upper and lower limits of the 95% uncertainty intervals (95% UIs). Figure S9. Global cases and age-standardized rates of YLDs of pancreatitis per 100,000 population by age and sex, 2017. Shading indicates the upper and lower limits of the 95% uncertainty intervals (95% UIs). Figure S10. Age-standardized incidence rates for pancreatitis for 195 countries and territories by SDI,2017. Expected values based on Socio-demographic Index and disease rates in all locations are shown as the black line. SDI = Sociodemographic Index. Figure S11. Age-standardized prevalence rates for pancreatitis for 195 countries and territories by SDI,2017. Expected values based on Socio-demographic Index and disease rates in all locations are shown as the black line. SDI = Sociodemographic Index. [file 12916_2020_1859_MOESM2_ESM.pdf]

Additional file 2

The global, regional, and national burden of pancreatitis in 195 countries and territories, 1990–2017: a systematic analysis for the Global Burden of Disease Study 2017

Fig S1: The age-standardized YLDs of pancreatitis in 2017 for 21 GBD regions, by sex……………………………………………………………..........2

Fig S2: The percentage change in age-standardized point prevalence of pancreatitis from 1990 to 2017 for 21 Global Burden of Disease regions by sex…….......3

Fig S3: The percentage change in age-standardized point incidence of pancreatitis from 1990 to 2017 for 21 Global Burden of Disease regions by sex. …….........4

Fig S4: The percentage change in age-standardized point YLDs of pancreatitis from 1990 to 2017 for 21 Global Burden of Disease regions by sex……….......5

Fig S5: Number of prevalent cases of pancreatitis from 1990 to 2017 for 21 Global Burden of Disease regions……………………………………6

Fig S6: Number of incident cases of pancreatitis from 1990 to 2017 for 21 Global Burden of Disease regions. ……………………………………7

Fig S7: Age-standardized YLDs rates of pancreatitis per 100 000 population in 2017, by country and territory……………………………………..8

Fig S8: Global cases and age-standardized rates of incidence of pancreatitis per 100 000 population by age and sex, 2017…………………..9

Fig S9: Global cases and age-standardized rates of YLDs of pancreatitis per 100 000 population by age and sex, 2017……………………….10

Fig S10: Age-standardized incidence rates for pancreatitis for 195 countries and territories by SDI,2017. ………………………………………..11

Fig S11: Age-standardized prevalence rates for pancreatitis for 195 countries and territories by SDI,2017. ……………………………………..12

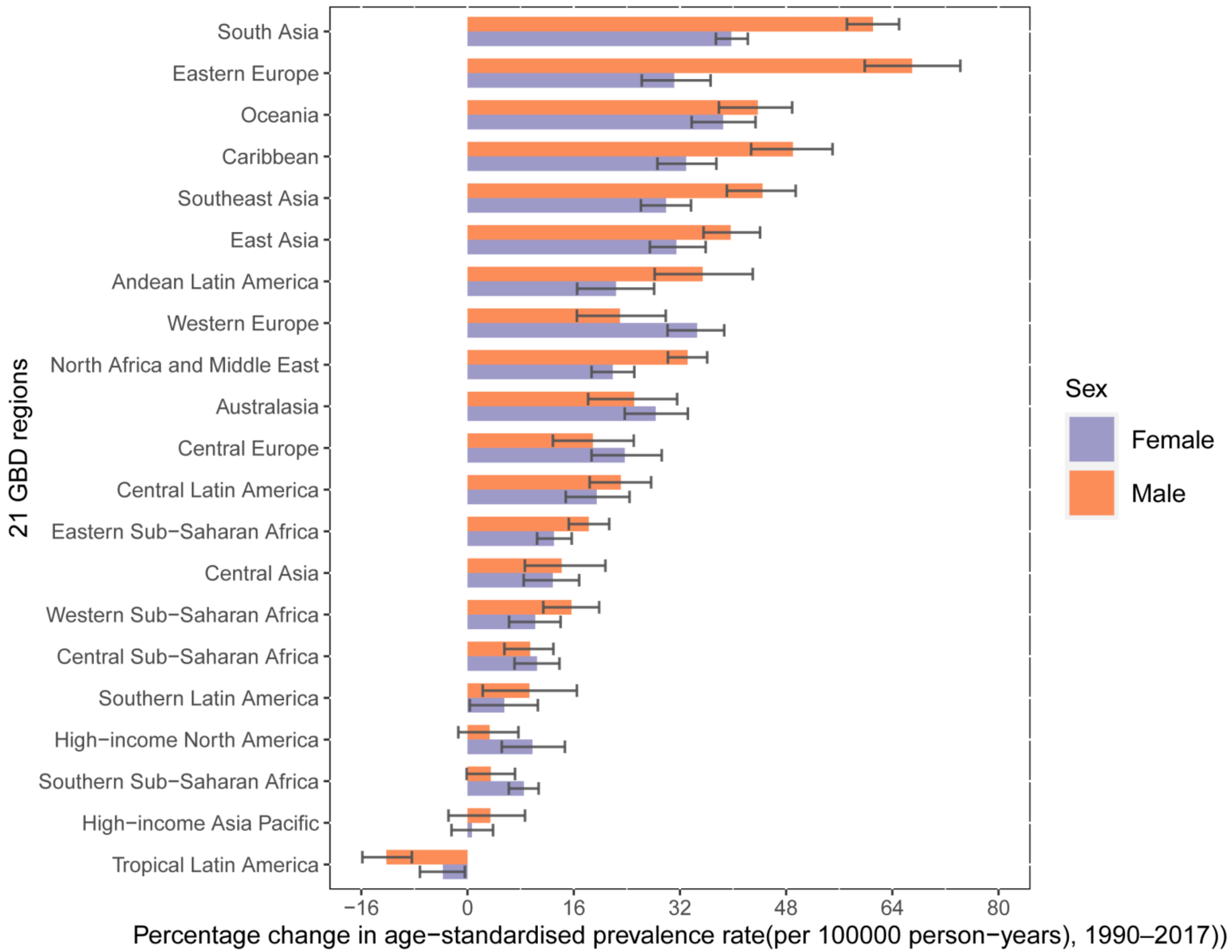

Fig S1: The percentage change in age-standardized point prevalence of pancreatitis from 1990 to 2017 for 21 Global Burden of Disease regions by sex.

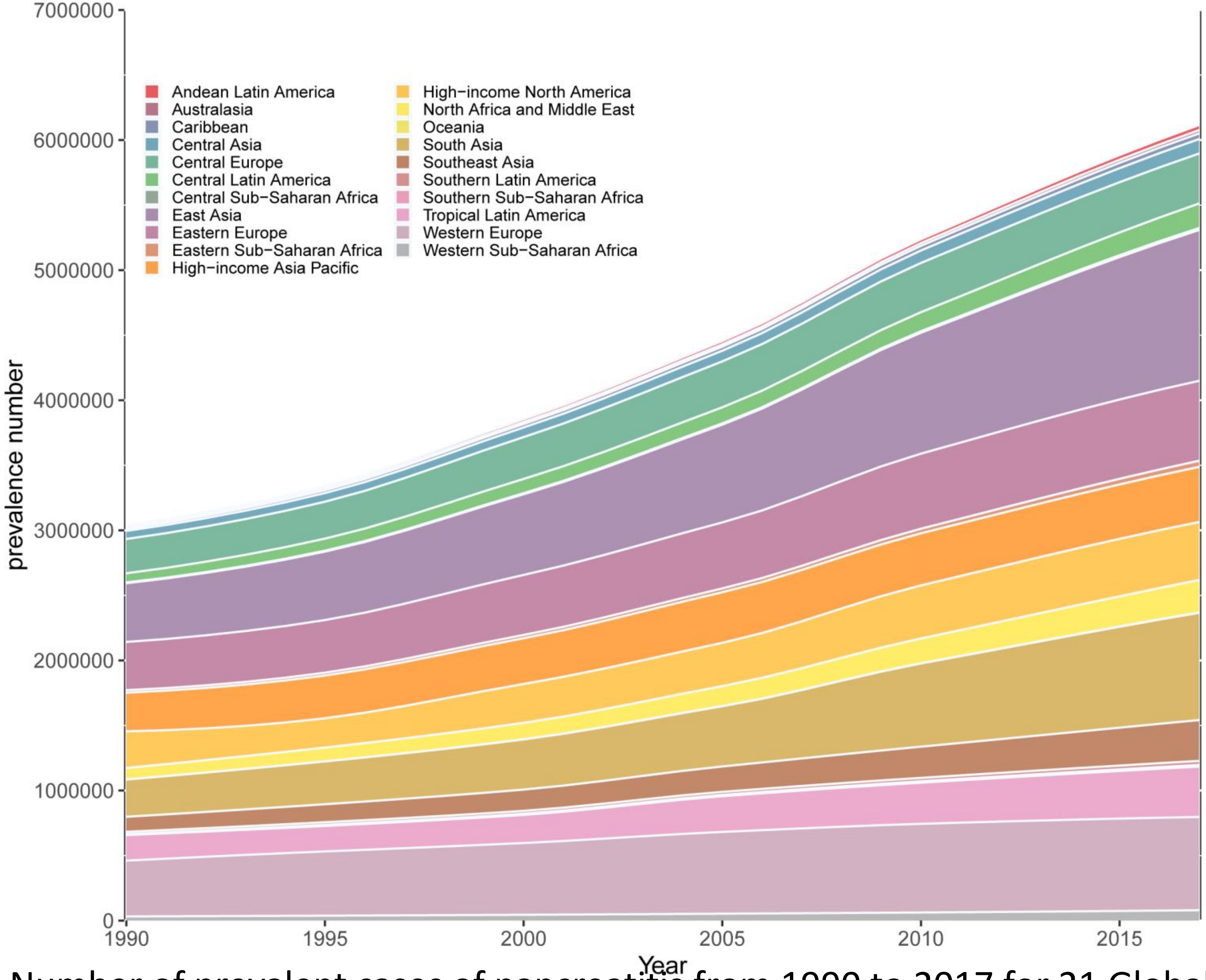

Fig S2: Number of prevalent cases of pancreatitis from 1990 to 2017 for 21 Global Burden of Disease regions

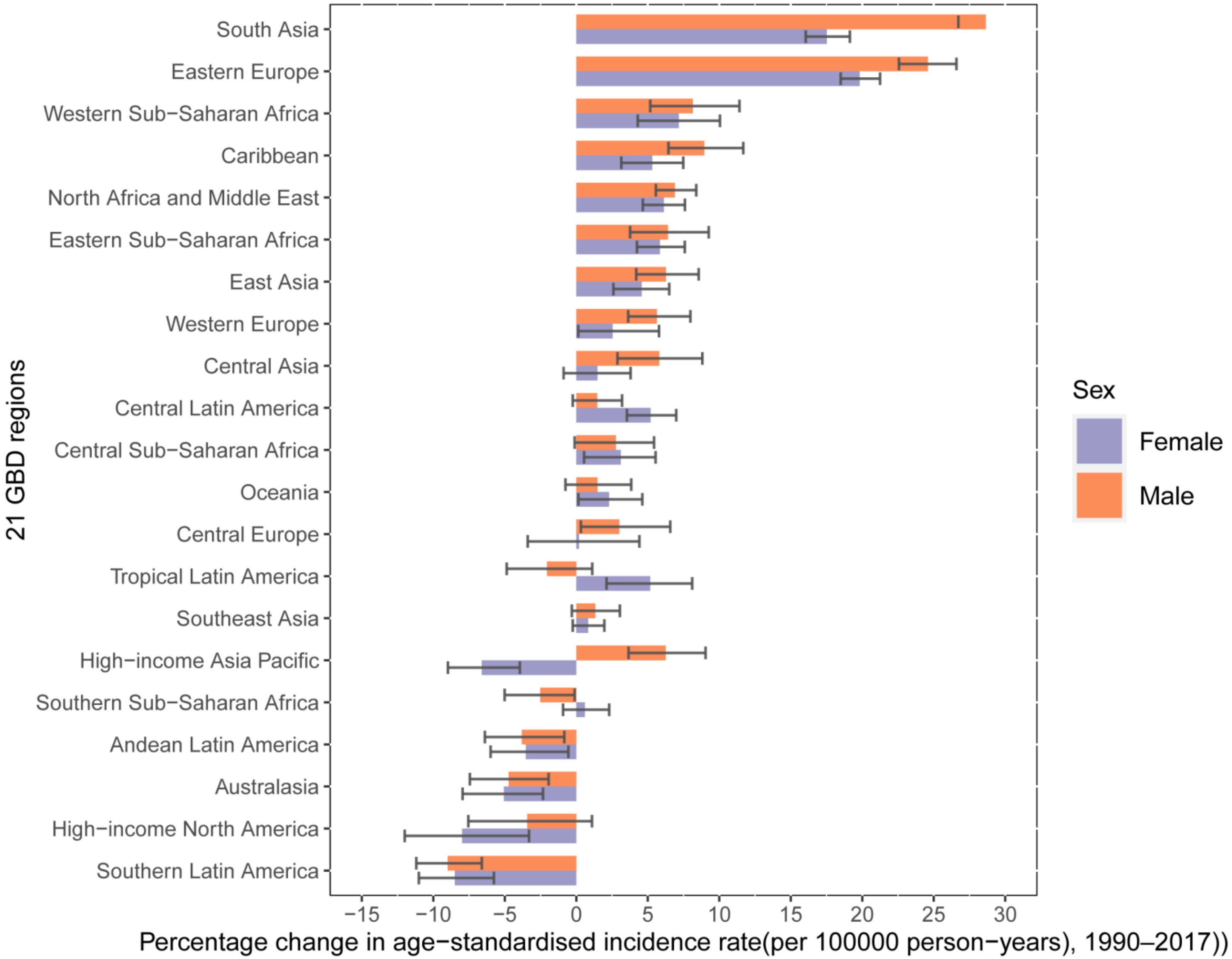

Fig S3: The percentage change in age-standardized point incidence of pancreatitis from 1990 to 2017 for 21 Global Burden of Disease regions by sex.

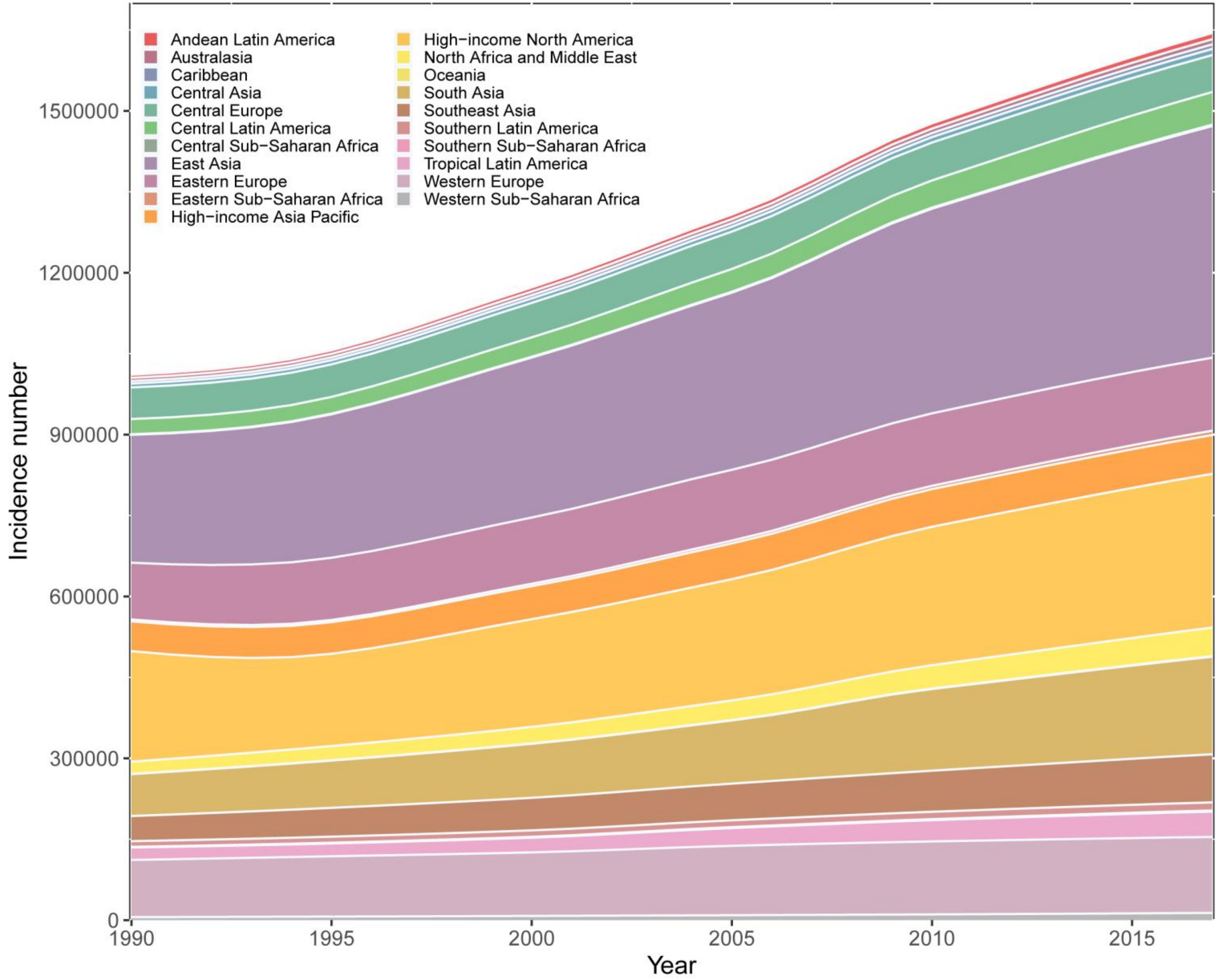

Fig S4: Number of incident cases of pancreatitis from 1990 to 2017 for 21 Global Burden of Disease regions.

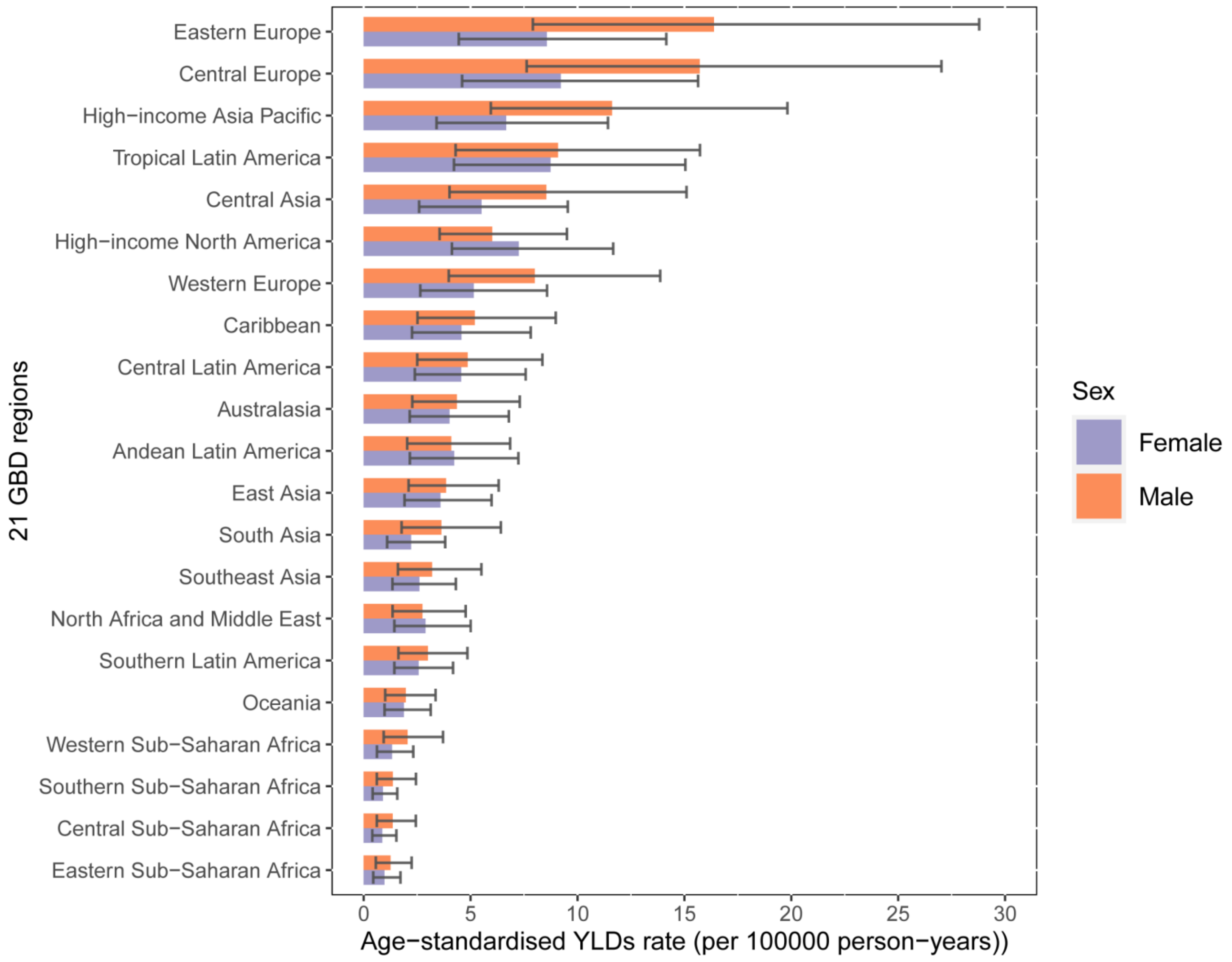

Fig S5: The age-standardized YLDs of pancreatitis in 2017 for 21 GBD regions, by sex.

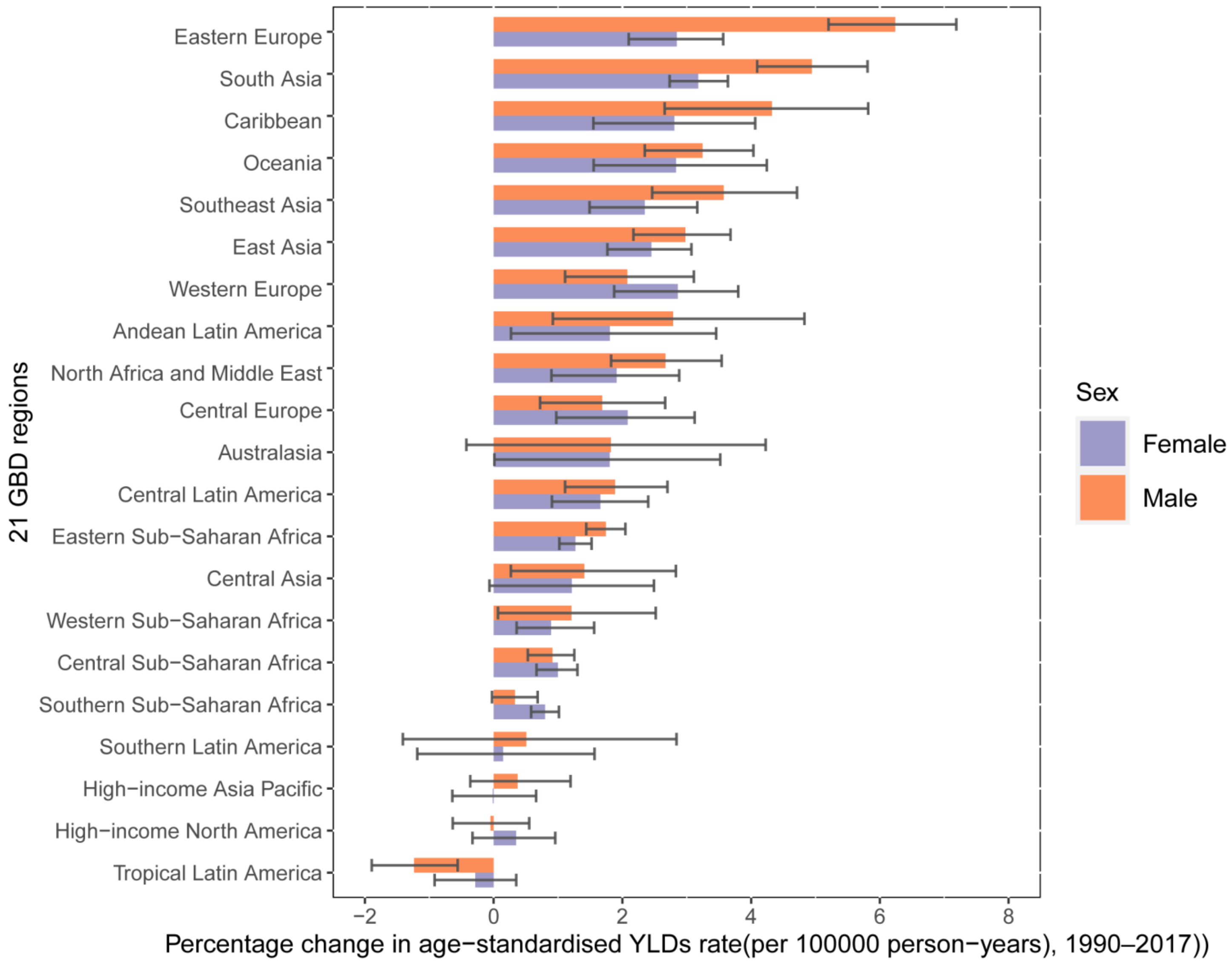

Fig S6: The percentage change in age-standardized point YLDs of pancreatitis from 1990 to 2017 for 21 Global Burden of Disease regions by sex.

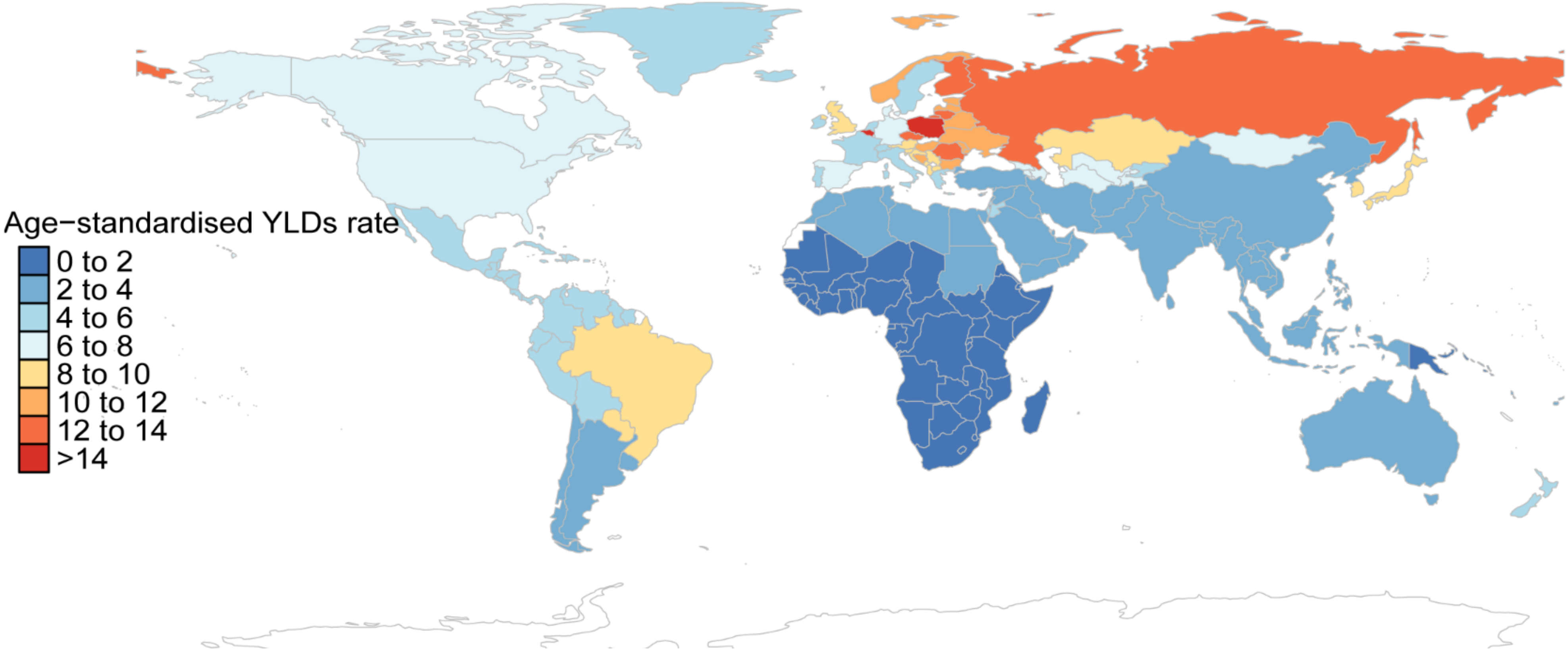

Fig S7: Age-standardized YLDs rates of pancreatitis per 100 000 population in 2017, by country and territory.

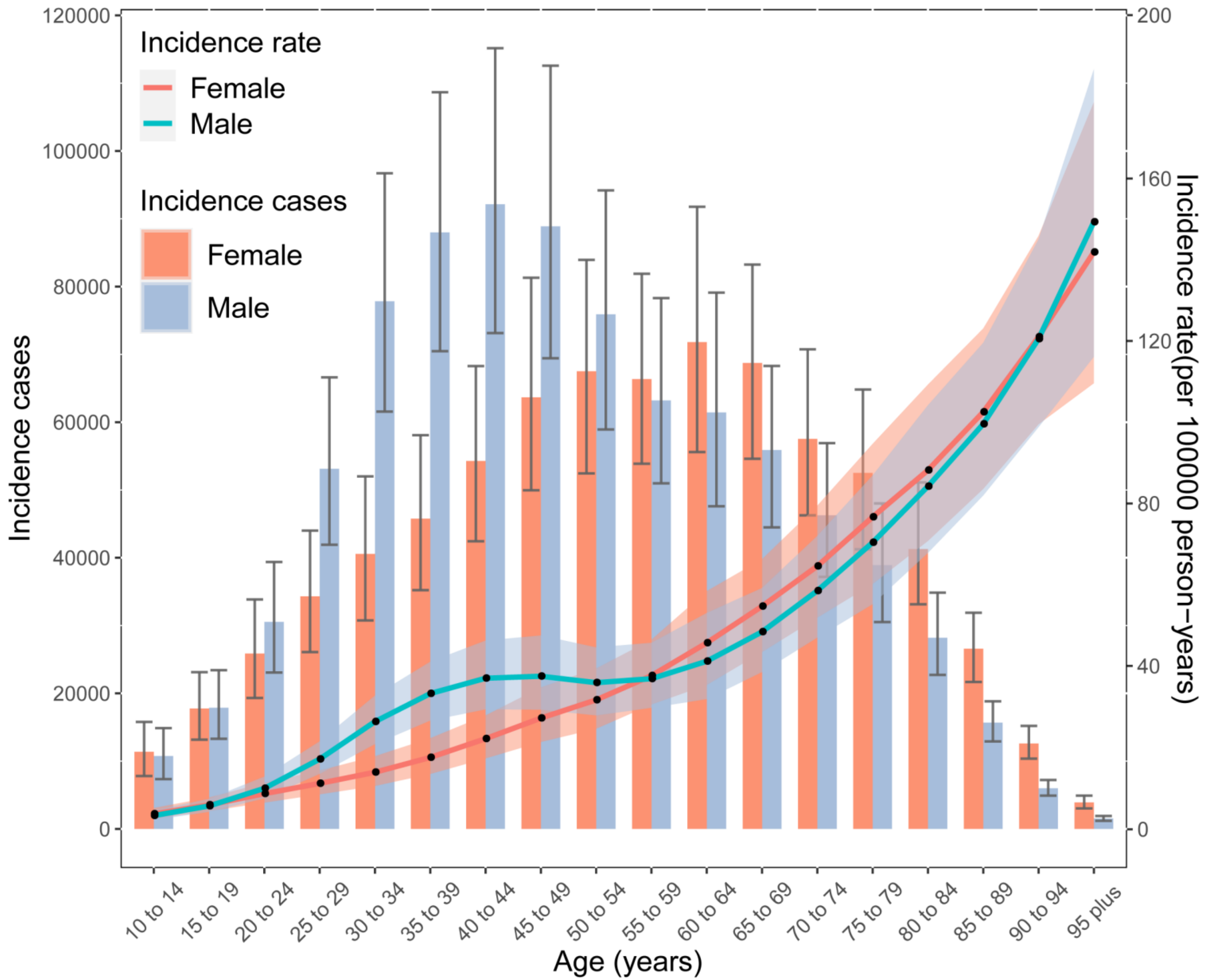

Fig S8: Global cases and age-standardized rates of incidence of pancreatitis per 100 000 population by age and sex, 2017

Shading indicates the upper and lower limits of the 95% uncertainty intervals (95% UIs).

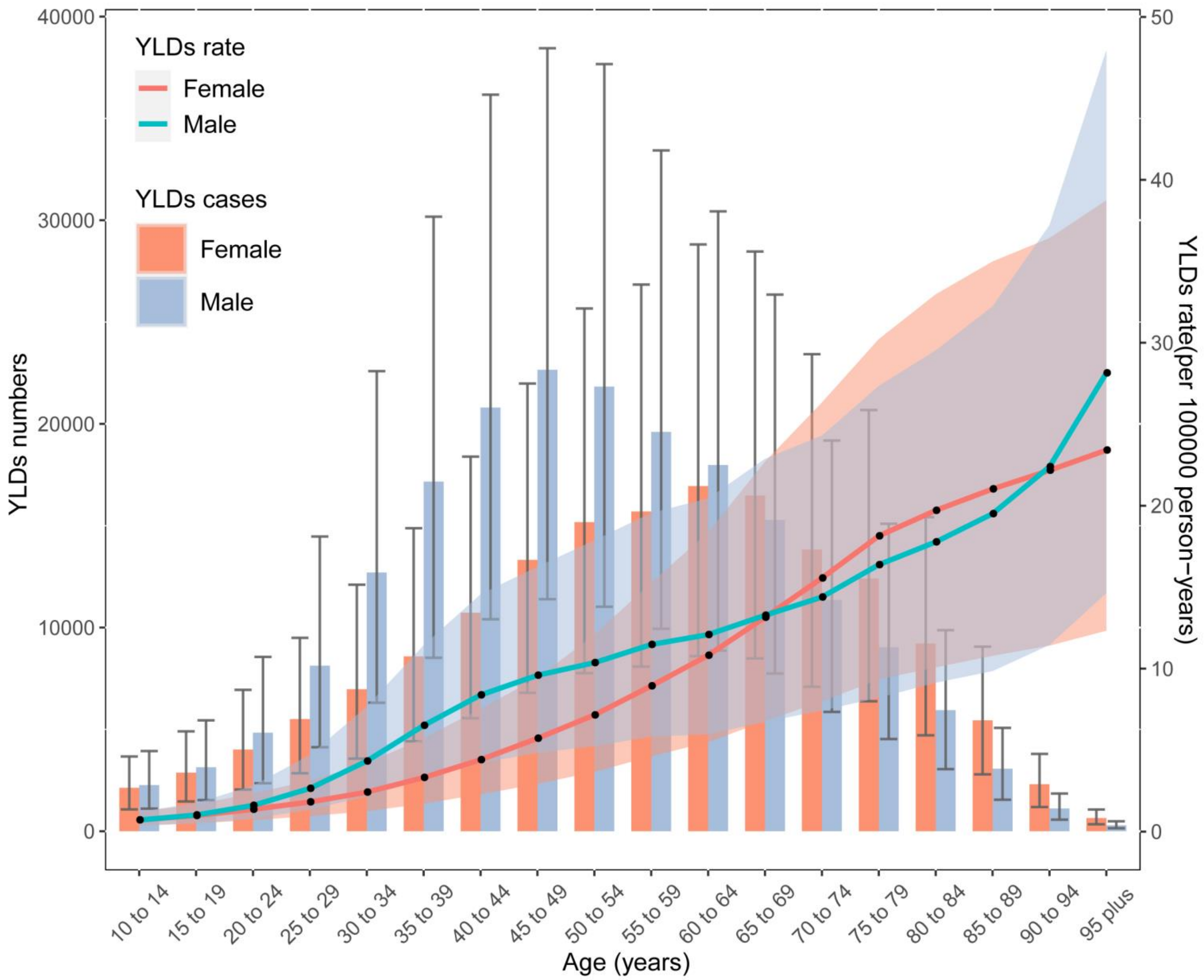

Fig S9: Global cases and age-standardized rates of YLDs of pancreatitis per 100 000 population by age and sex, 2017

Shading indicates the upper and lower limits of the 95% uncertainty intervals (95% UIs).

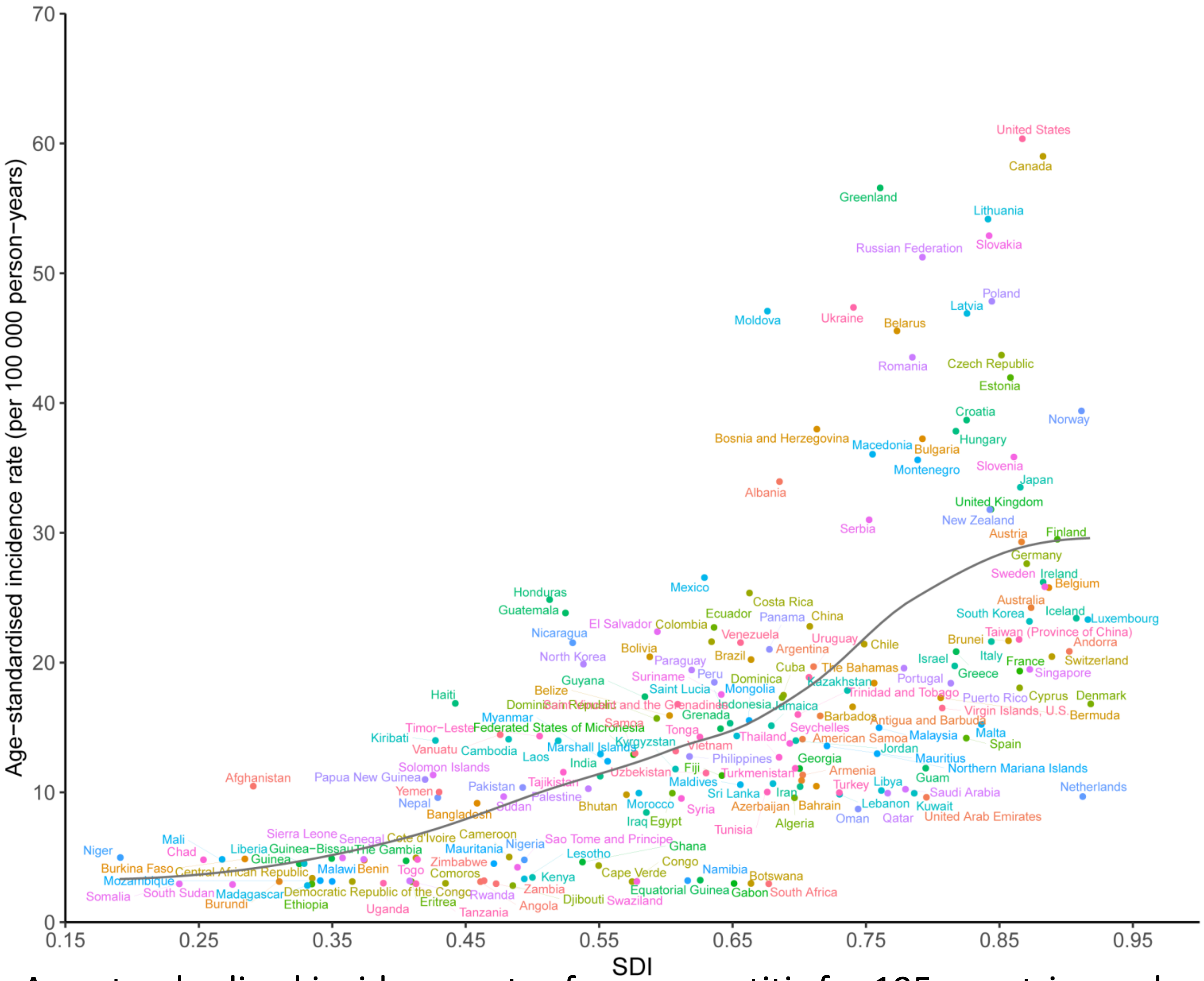

Fig S10: Age-standardized incidence rates for pancreatitis for 195 countries and territories by SDI,2017.

Expected values based on Socio-demographic Index and disease rates in all locations are shown as the black line.
SDI= Sociodemographic Index.

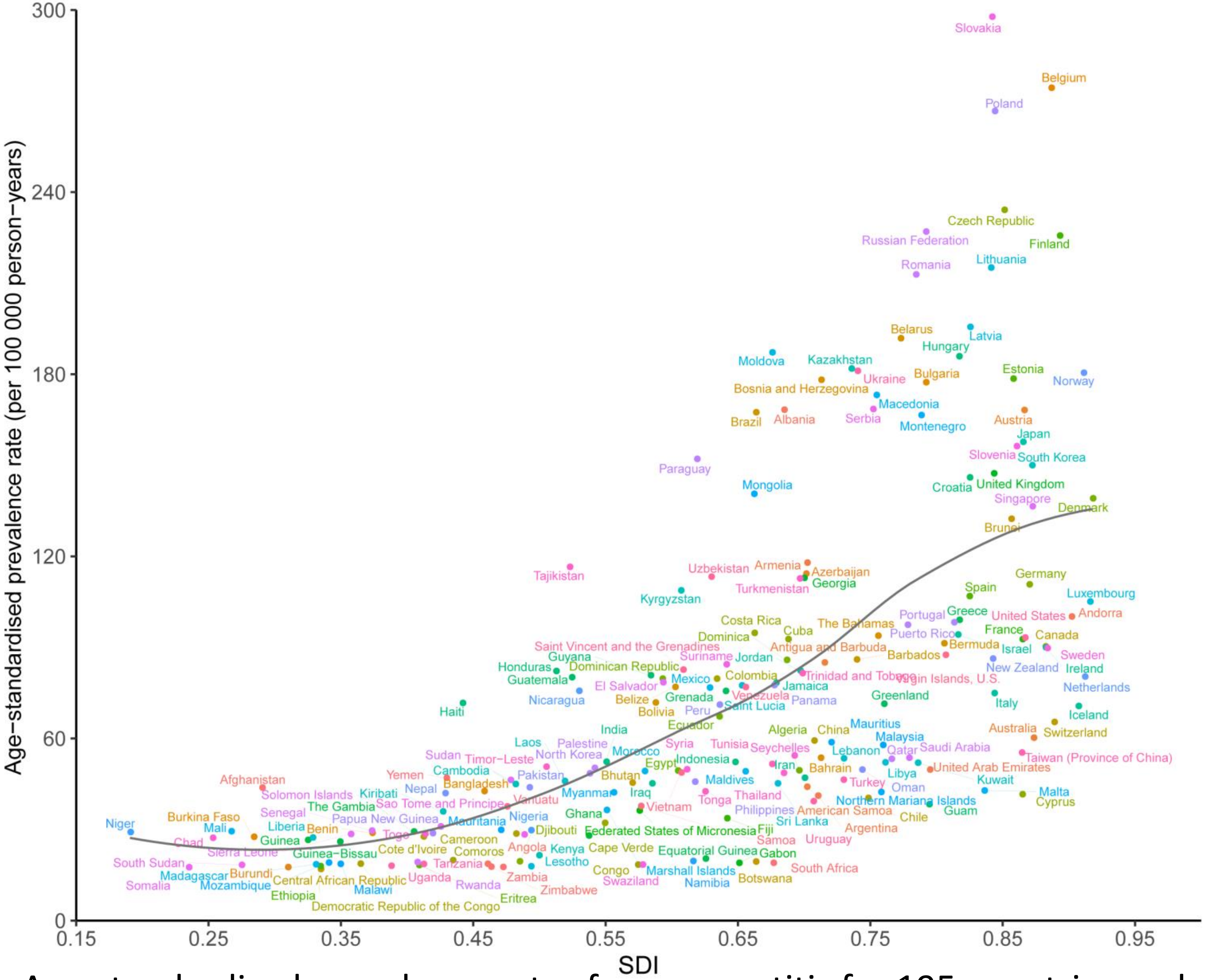

Fig S11: Age-standardized prevalence rates for pancreatitis for 195 countries and territories by SDI,2017.

Expected values based on Socio-demographic Index and disease rates in all locations are shown as the black line.
SDI= Sociodemographic Index.
